# Genomic remnants of ancestral methanogenesis and hydrogenotrophy in Archaea drive anaerobic carbon cycling

**DOI:** 10.1126/sciadv.abm9651

**Published:** 2022-11-04

**Authors:** Panagiotis S. Adam, George E. Kolyfetis, Till L. V. Bornemann, Constantinos E. Vorgias, Alexander J. Probst

**Affiliations:** ^1^ Environmental Microbiology and Biotechnology, Faculty of Chemistry, University of Duisburg-Essen, Universitätsstraße 5, 45141 Essen, Germany.; ^2^ Department of Biochemistry and Molecular Biology, Faculty of Biology, National and Kapodistrian University of Athens, Panepistimiopolis Zografou, 15784 Athens, Greece.; ^3^ Centre for Water and Environmental Research (ZWU), University of Duisburg-Essen, Universitätsstraße 5, 45141 Essen, Germany.; ^4^ Research Center One Health Ruhr, Research Alliance Ruhr, Environmental Metagenomics, University of Duisburg-Essen, Universitätsstraße 5, 45141 Essen, Germany.

## Abstract

Anaerobic methane metabolism is among the hallmarks of Archaea, originating very early in their evolution. Here, we show that the ancestor of methane metabolizers was an autotrophic CO_2_-reducing hydrogenotrophic methanogen that possessed the two main complexes, methyl-CoM reductase (Mcr) and tetrahydromethanopterin-CoM methyltransferase (Mtr), the anaplerotic hydrogenases Eha and Ehb, and a set of other genes collectively called “methanogenesis markers” but could not oxidize alkanes. Overturning recent inferences, we demonstrate that methyl-dependent hydrogenotrophic methanogenesis has emerged multiple times independently, either due to a loss of Mtr while Mcr is inherited vertically or from an ancient lateral acquisition of Mcr. Even if Mcr is lost, Mtr, Eha, Ehb, and the markers can persist, resulting in mixotrophic metabolisms centered around the Wood-Ljungdahl pathway. Through their methanogenesis remnants, Thorarchaeia and two newly reconstructed order-level lineages in Archaeoglobi and Bathyarchaeia act as metabolically versatile players in carbon cycling of anoxic environments across the globe.

## Teaser

Partial loss of ancestral H_2_/CO_2_ methanogenesis creates mixotrophic metabolisms in globally distributed anaerobic Archaea.

## INTRODUCTION

Many Archaea are capable of performing methanogenesis, producing methane under anaerobic conditions as part of their energy metabolism. Methanogenesis and its reversal as anaerobic methane oxidation (AMO) are exclusively encountered among Archaea (*1*). Under traditional taxonomic schemes [non–Genome Taxonomy Database (GTDB) (*2*) taxa are denoted with an asterisk; GTDB names correspond to r202], all known methanogens were members of the Euryarchaeota* and classified into two groups: Class I methanogens or Methanomada* (Methanopyri, Methanobacteria, and Methanococci) and Class II methanogens (Methanosarcinia and Methanomicrobia) (*1*). While the composition of Class I methanogens has remained constant over the years, several lineages with methane metabolism (methanogenesis and/or AMO) are now known to be related to the Class II methanogens. Collectively, they belong to Halobacteriota (*3*, *4*) and include the Methanocellia, c__Bog-38 [Methanoflorentales* (*5*); we include the taxonomic rank prefix for GTDB generic names], Methanonatronarchaeia (*6*), o__ANME-1 [Methanophagales* (*7*)], and Archaeoglobi (*8*–*10*). The distribution of methane metabolism currently extends to most major Euryarchaeota* clades and some lineages of Thermoproteota (traditionally, the TACK* superphylum) (*1*, *11*).

**Fig. 1.**
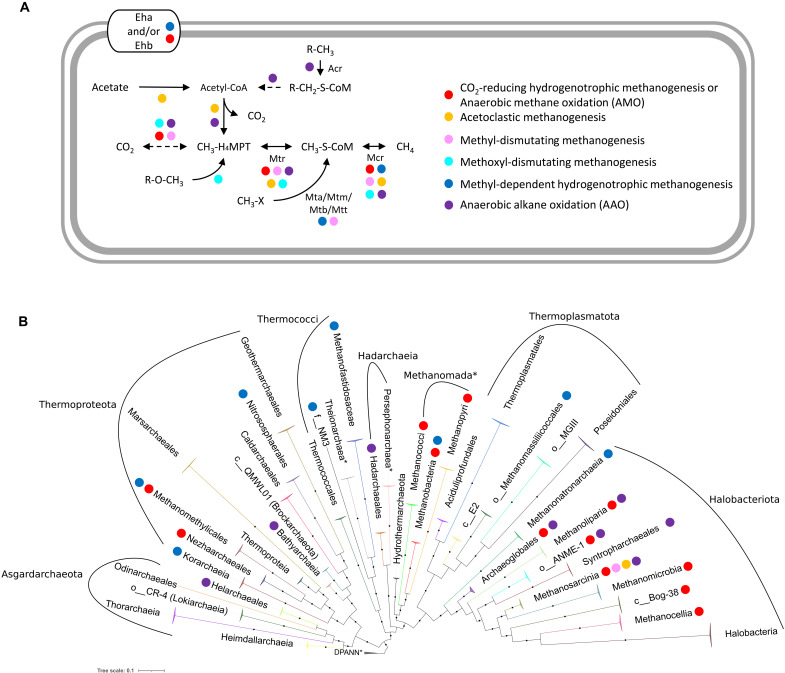
Possible configurations and taxonomic distribution of anaerobic methane and alkane metabolism in Archaea. (**A**) Diagram of individual metabolic pathways comprising the different types of methanogenesis, AMO, and anaerobic alkane oxidation (AAO). Colored dots correspond to CO_2_-reducing hydrogenotrophic methanogenesis or AMO (red), acetoclastic methanogenesis (orange), methyl-dismutating methanogenesis (pink), methoxyl-dismutating methanogenesis (turquoise), methyl-dependent hydrogenotrophic methanogenesis (blue), and AAO (purple). We have adopted the following simplifications: (i) The dashed line connecting CO_2_ with methyl-H_4_MPT corresponds to the WLP H_4_MPT methyl branch, disregarding small variations in the enzymes used, and the cases where formate is used as a substrate for methanogenesis; (ii) the dashed line from R-CH_2_-S-CoM to acetyl–coenzyme A (CoA) corresponds to β oxidation. The AAO pathways (long-chain alkanes, coupled to methanogenesis) are modeled after (*35*); (iii) for the conversion from acetate to acetyl-CoA, we do not discriminate whether it is performed in one (acetyl-CoA synthetase) or two (acetate kinase and phosphate transacetylase) reactions and include it only for acetoclastic methanogens even if the genes are present elsewhere; (iv) we overlook the H_4_MPT branch and Mtr in *Methanosphaera*, as they are not used in its methyl-dependent hydrogenotrophic methanogenesis. (**B**) ML phylogeny of Archaea, based on a supermatrix of 36 Phylosift markers (6021 amino acid positions) rooted at the DPANN*. Euryarchaeota* contain Thermococci, Hadarchaeia, Methanomada*, Thermoplasmatota, and Halobacteriota. Black circles indicate strongly supported branches (ultrafast bootstrap ≥ 95, aLRT SH-like ≥ 80), and other colored circles correspond to the metabolisms in (A). Any non-GTDB nomenclature is marked with an asterisk. For GTDB names without standard taxonomic suffixes, we use the taxonomic rank double underscore designation of GTDB. GTDB names and taxonomy correspond to r202. In phylogeny labels or elsewhere where NCBI names are used in conjunction with assembly accessions, we omit asterisks. Additional remarks on the phylogeny are given in Supplementary Results and Discussion.

Inferring methane metabolism in a metagenome-assembled genome (MAG) is tied to the presence of genes encoding methyl–coenzyme M (CoM) reductase (Mcr), the complex that catalyzes the reversible reduction of a CoM-attached methyl group to methane. The presence of the tetrahydromethanopterin-CoM methyltransferase (Mtr) complex genes in the same genome usually implies CO_2_-reducing hydrogenotrophic methanogenesis or AMO. In the reductive/methanogenic direction, Mtr transfers a methyl moiety from tetrahydromethanopterin (H_4_MPT) at the end of the Wood-Ljungdahl pathway (WLP) to CoM-SH while exporting sodium ions. AMO consists of the reverse reactions with the same enzymes. CO_2_-reducing hydrogenotrophic methanogens (Fig. 1, red circle) discovered through metagenomic studies are found in the Nezhaarchaeales (Nezhaarchaeota*) (*9*) and Methanomethylicales (Verstraetearchaeota*) (*12*).

The same enzymatic configuration with Mcr, Mtr, and the WLP (methyl or carbonyl branch) is found in acetoclastic (Fig. 1, orange circle) and methyl-dismutating (*1*) (Fig. 1, pink circle; formerly “methylotrophic”) methanogenesis, both of which have only been found in Methanosarcinia. In acetoclastic methanogenesis, acetate is converted to acetyl-coenzyme A (CoA). Then, through the WLP carbonyl branch, the carbonyl moiety is oxidized to CO_2_ and the methyl moiety is attached to H_4_MPT and ultimately reduced to methane through Mtr and Mcr. In methyl-dismutating methanogenesis, methyl groups from various substrates, such as methanol and methylamines, are transferred to CoM-SH. The oxidation of one moiety to CO_2_ via the WLP provides the necessary energy to reduce another three to methane. *Methermicoccus shengliensis* in Methanosarcinia_A was recently found to be capable of methoxyl-dismutating methanogenesis in which the methyl moiety is transferred to H_4_MPT instead of CoM-SH (*13*). There exist further variations in the carbon source (e.g., choline) and electron donor (e.g., iron or formate) in specific methanogens exist (*14*).

In recent years, there has been an abundance of MAGs coding for methyl-dependent hydrogenotrophic (Fig. 1, blue circle; formerly “hydrogen-dependent methylotrophic”) methanogenesis, first discovered in *Methanosphaera* (Methanobacteriales) and Methanomassiliicoccales. A methanol, methylamine, or methanethiol methyltransferase attaches a methyl group to CoM-SH, and then Mcr reduces it to methane. Hydrogen is the electron donor for this process, and Mtr is absent. Methyl-dependent hydrogenotrophic methanogenesis has been found in several lineages: Methanofastidiosaceae (Methanofastidiosa*) (*15*), f__NM3 (Nuwarchaeales*) (*11*, *16*), Methanomethylicales (*8*, *9*, *11*, *17*), Korarchaeia (Korarchaeota*) (*9*, *11*, *18*), and Nitrososphaeria (specifically the former Thaumarchaeota*, containing the current Nitrososphaerales and Conexivisphaerales) (*8*).

The phylogeny of Mcr is only partially congruent with published archaeal species trees (*1*). Other than the canonical Mcr used in methanogenesis and AMO, a divergent Mcr-like (or alkyl-CoM reductase, Acr) clade has been associated with anaerobic alkane oxidation (AAO) across Archaea (Fig. 1, purple circle). Acr has been found in Syntropharchaeales (*19*), Methanoliparia (*11*), Methanosarcinia (*11*, *20*, *21*), Bathyarchaeia (*11*, *22*), Helarchaeales (Helarchaeota*) (*23*), Hadarchaeia (Hadesarchaea*) (*8*, *9*), and Archaeoglobi (*9*, *24*). The possible pathway configurations of methane metabolism and AAO are presented in Fig. 1A, and an updated reference phylogeny of Archaea containing the aforementioned lineages is provided in Fig. 1B. We should note that the vast majority of MAGs, where the genomic capability for methanogenesis, AMO, or AAO has been found, correspond to uncultured Archaea, and thus, the phenotypic inferences are putative.

Several other genes that are tentatively related to methane metabolism exist. Some of them have been dubbed “methanogenesis markers” by virtue of their taxonomic distribution closely matching that of methanogenesis and AMO (*11*). However, they are rarely used in metabolic annotations. Many of them are domains of unknown function (DUFs) (*25*), mirroring the large number of DUFs among the auxiliary genes of the WLP (*26*). It has been proposed that the presence of these genes outside methane- or alkane-metabolizing lineages could indicate that they are metabolic remnants repurposed into other pathways (*11*, *27*, *28*). In the CO_2_-reducing hydrogenotrophic methanogens of Methanomada*, the first step of CO_2_ reduction to formylmethanofuran in methanogenesis and carbon fixation, respectively, is driven by two anaplerotic [NiFe] hydrogenase complexes, Eha (group 4h) (*29*) and Ehb (group 4i) (*30*, *31*).

While Mcr is never encountered outside of methane metabolism and AAO (for Acr), many archaeal and bacterial lineages possess MtrAH, the two methyltransferase subunits of Mtr. The role of these subunits in other types of metabolism is currently unclear, but it is hypothesized that they funnel methyl moieties into the WLP (*32*). Another long-standing debate concerns how ancient methane metabolism is among Archaea (*1*) and whether its original form was CO_2_-reducing hydrogenotrophic (*12*) or methyl-dependent hydrogenotrophic (*16*). The role of methanogenesis markers in methane/alkane metabolism and their origins are largely unstudied. In this study, we set out to address these questions, starting with the evolution and metabolic roles of methanogenesis markers. Through phylogenomic methods, we show that the earliest form of methane metabolism was CO_2_-reducing hydrogenotrophic methanogenesis mediated by Mcr, Mtr, Eha, and Ehb, and with many methanogenesis markers being present. The evolutionary rates of the membrane-bound complexes (Mtr, Eha, and Ehb) are in part dependent on whether specific residues are exposed or transmembrane. Analysis of subsurface metagenomes indicates that a variety of mixotrophic metabolisms can emerge in lineages that possess Mtr subunits and markers as remnants of methanogenesis after the loss of Mcr.

## RESULTS AND DISCUSSION

### The original methane metabolism was CO_2_-reducing hydrogenotrophic methanogenesis

Our first milestone was to assemble the largest possible set of proteins putatively related to methane metabolism. Among the methanogenesis marker sets found in the literature (*11*, *27*), many contain DUFs. Thus, to search for additional potential markers, we surveyed the taxonomic distribution of archaeal DUFs, looking for co-occurrences with methane metabolism. A DUF was defined as “archaeal” if at least half of its taxonomic distribution in UniProt (*33*) consisted of Archaea. Ultimately, we examined the distribution and phylogenies of (i) 155 such DUFs (data S1), along with (ii) the 38 markers of Borrel *et al.* (*11*) (data S2 and table S1), and (iii) “proteins that are specific for methanogens” and “proteins that are specific to certain subgroups of methanogens” from Gao and Gupta (*27*) (data S3). Before our study, no phylogenies were available for most of these proteins. We searched for homologs against a local database consisting of 1808 archaeal and 25118 bacterial genomes, plus another 14494 viral genomes for gene sets (i) and (iii) (data S1).

**Fig. 2.**
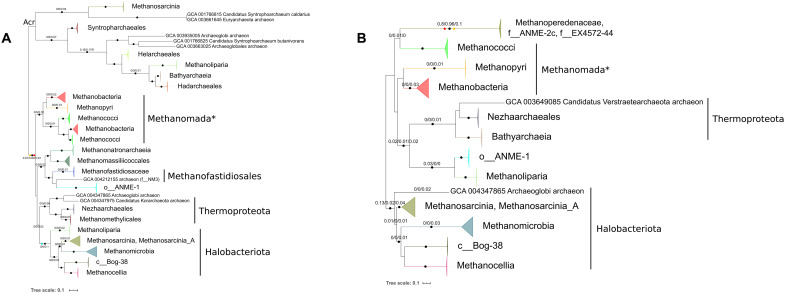
Evolution of the Mcr and Mtr complexes. Maximum likelihood (ML) phylogenies of (**A**) McrABG (1141 amino acid positions) and (**B**) MtrABCDEFG (1079 amino acid positions). Black circles indicate strongly supported branches (ultrafast bootstrap ≥ 95, aLRT SH-like ≥ 80), red circles correspond to the MAD root, orange circles correspond to MinVar, and blue circle corresponds to NONREV. Branch values correspond to rootstrap supports for MAD, MinVar, and NONREV, respectively. In Mtr, the NONREV root is within a collapsed clade. The NONREV rooting was spurious, and its rootstrap supports were low, particularly for Mtr, Eha, and Ehb, probably due to the small number of positions in the concatenations; it was mainly included to compare with the other rooting algorithms.

**Fig. 3.**
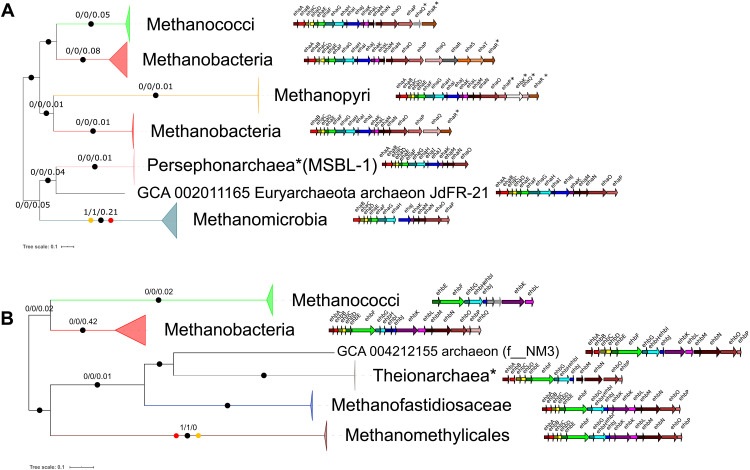
Evolution and comparative genomics of Eha and Ehb. ML phylogenies of (**A**) EhaBCDEFGHJLMNO (1828 amino acid positions) and (**B**) EhbABCDEFGHIJKLMNOP (2770 amino acid positions), along with the genomic organization of the hydrogenase clusters in a representative genome for each major clade. Black circles indicate strongly supported branches (ultrafast bootstrap ≥ 95 aLRT SH-like ≥ 80), red circles correspond to the MAD root, and orange circles correspond to MinVar. Branch values correspond to rootstrap supports for MAD, MinVar, and NONREV, respectively. For both Eha and Ehb, the NONREV root is within a collapsed clade. Subunits marked with asterisks are problematic in terms of their homology and/or nomenclature (see Supplementary Methods). The taxa used as illustrative cases for the cluster organization were as follows: *Methanothermobacter marburgensis* str. Marburg (GCA_000145295; Methanobacteria), *Methanocaldococcus jannaschii* DSM 2661 (GCA_000091665; Methanococci), *Methanopyrus kandleri* AV19 (GCA_000007185; Methanopyri), *Methanothermobacter tenebrarum* (GCA_003264935; Methanobacteria small clade in Eha), *Methanospirillum hungatei* JF-1 (GCA_000013445; Methanomicrobia), Euryarchaeota archaeon JdFR-21 (GCA_002011165; NRA7*/Mnemosynellales*), candidate division MSBL1 archaeon SCGC-AAA259E19 (GCA_001549095; MSBL1*/Persephonarchaea*), *Candidatus* Methanomethylicus mesodigestum (GCA_001717035; Methanomethylicales), Arc I group archaeon ADurb1013_Bin02101 (GCA_001587595; Methanofastidiosaceae), Theionarchaea archaeon DG-70-1 (GCA_001595815; Theionarchaea*), and archaeon (GCA_004212155; f__NM3).

These protein sets include the subunits of Mcr and Mtr and some of Eha and Ehb. To obtain complete protein sets for the latter two, we had to recover homologs of any remaining subunits retroactively by extrapolating from taxonomic distribution and synteny. To improve the phylogenetic signal, we tested for congruence among single-gene phylogenies and assembled concatenated alignments of McrABG (Fig. 2A and data S4), MtrABCDEFG (Fig. 2B and data S5), EhaBCDEFGHJLMNO (Fig. 3A and data S6 and S7), and EhbABCDEFGHIJKLMNOP (Fig. 3B and data S6 and S8). We determined outgroup-free roots of all phylogenies with both nonreversible models (NONREV) and the minimal ancestor deviation (MAD) and minimum variance (MinVar) methods and tested them with rootstraps (data S9).

In the Mcr phylogeny (Fig. 2A), in the canonical Mcr clade, we recover the strongly supported respective monophylies of Methanomada*, Halobacteriota, Thermoproteota, and Methanofastidiosales (Methanofastidiosaceae and f__NM3). The topology in each of these clades is in agreement with the species tree (Fig. 1B). Similarly, from the internal topologies in each clade’s constituent lineages (e.g., Methanopyri, Methanobacteria, and Methanococci for Methanomada*; the collapsed branches of Fig. 2A), we did not find evidence that said lineage acquired Mcr from an ancient transfer event (data S4). There were, of course, cases of transfers and/or homologous recombinations, such as the Methanobacteria clade in Methanococci. These patterns of vertical inheritance allow us to infer the presence of Mcr in the ancestors of Methanomada*, Halobacteriota, Thermoproteota, and Methanofastidiosales and, by extension, their common ancestor. There is a caveat that none of the relationships among these four major clades is strongly supported. Outgroup-free rooting turned out to be less informative than we had anticipated. The NONREV root (Fig. 2A, blue circle) at Halobacteriota is consistent with the root of Archaea proposed by Raymann *et al.* (*34*). Under the MAD (Fig. 2B, red circle) and MinVar (Fig. 2B, orange circle) root, after the split between Mcr and Acr, there is a split between Methanomada* and other archaeal groups. The resulting backwards branching pattern for Euryarchaeota* would require us to infer at least two ancient transfer events to be compatible with the species tree. The disagreement among rooting methods also affects the inferred origin of Acr, resulting in either a basal Mcr/Acr split or an origin of Acr within Euryarchaeota*. This ambiguous origin of Acr has been noted elsewhere (*9*).

A vertically inherited Mcr is found in Ca. Methylarchaeum tengchongensis* (JZ-2 bin_220), a methyl-dependent hydrogenotrophic methanogen in Thaumarchaeota* that was not part of our genome set for this study. In agreement with our results, the same study proposed that Mcr genes in Thermoproteota originated from vertical inheritance (*8*). A sequence from the Archaeoglobi member Ca. Methanomixophus hydrogenotrophicum* (*10*) (s__WYZ-LMO2 sp004347865) branches inside Thermoproteota and o__ANME-1 are found inside Methanofastidiosales. Both are typical cases of lateral acquisition of Mcr that have been observed before (*8*, *10*, *11*, *16*). For the methyl-dependent hydrogenotrophic methanogens in Methanonatronarchaeia and Methanomassiliicoccales, if Mcr were inherited vertically, we would expect their positions in the Mcr tree to agree with the species tree (Fig. 1B); that is inside and as sister to the Halobacteriota, respectively. Instead, they cluster together next to the Methanofastidiosales, probably as a result of an ancient lateral transfer from an unknown donor, as previously suggested as a possibility (*16*). Several previous publications (*1*, *8*, *9*, *11*, *12*) have inferred the presence of Mcr and, by extension, methane metabolism early in Archaea, probably even in their last common ancestor.

Following the same logic as for Mcr, in the Mtr phylogeny (Fig. 2B), we obtain strongly supported monophylies and agreement with the species tree for Halobacteriota and Thermoproteota. Thus, their respective ancestors possessed Mtr, and the genes were inherited mostly vertically, except for a transfer event of indeterminable direction between the ancestors of Methanomicrobia and c__Bog-38 plus Methanocellia. We did not recover the collective monophyly of Methanomada*, but Methanobacteria, Methanopyri, and Methanococci were individually strongly monophyletic. Even so, tracing Mtr to the common ancestor of Halobacteriota and Thermoproteota is enough to deduce that Mtr is as ancient as Mcr, as Methanomada* branched later (Fig. 1B). All Mtr complexes from Methanoliparia (*35*) and AMO lineages are outside the Halobacteriota clade. Thus, these Mtr complexes originated from ancient transfer events, one for the branch containing Methanoperedenaceae (ANME-2d*), f__ANME-2c, and f__EX4572-44, and one for o__ANME-1 and Methanoliparia. We disregard all the outgroup-free roots for Mtr, because they result in nonsensical scenarios. MAD (Fig. 2B, red circle) and MinVar (Fig. 2B, orange circle) would suggest that there was a split between some AMO Methanosarcinia and all other Archaea before the origin of Thermoproteota or Halobacteriota. Likewise, NONREV places the origin of Mtr in Methanobacteria (Fig. 2B, within collapsed clade).

Collectively, we can deduce that the common ancestor of Methanomada*, Halobacteriota, and Thermoproteota possessed both Mcr and Mtr and, by extension, was a CO_2_-reducing hydrogenotrophic methanogen. This effectively corresponds to the common ancestor of non-DPANN* Archaea that we will henceforth call “Last Methane-metabolizing Ancestor” (LMA). As opposed to the WLP that is also found in the DPANN* (in Altiarchaeota) (*26*, *36*), we cannot confidently trace Mcr and Mtr to the Last Archaeal Common Ancestor. While the LMA could have performed AMO through a simple reversal of CO_2_-reducing hydrogenotrophic methanogenesis, we consider it extremely unlikely. The reason is that all extant AMO Archaea have obtained at least one of the Mcr and Mtr complexes through horizontal gene transfers, so AMO probably emerged later multiple times independently, as proposed elsewhere (*11*). AAO coupled to methanogenesis also emerged late, because Methanoliparia have acquired their Mtr horizontally (Fig. 2B). Our inference about the original type of methane metabolism being CO_2_-reducing hydrogenotrophic methanogenesis has previously been considered as a possible scenario (*8*, *11*, *12*), albeit with less data. We do not consider acetoclastic, methoxyl-dismutating methanogenesis, and the use of other carbon sources as ancient, because they have very limited taxonomic distributions. For example, acetoclastic and methoxyl-dismutating methanogenesis are thought to be recent inventions in the Methanosarcinia and Methanosarcinia_A (*1*). For the same reason, we do not take into account alternative electron donors. Even the use of formate as an electron donor has a narrow taxonomic distribution (Methanomicrobia, a few Methanococci and Methanobacteria), and these methanogens can also gain electrons from hydrogen (*14*).

A recent study by Wang *et al.* (*9*) proposed that the LMA was a methyl-dependent hydrogenotrophic methanogen, with the sequences in Nezhaarchaeales having been transferred from o__ANME-1/Methanoliparia (*16*). The methodological approaches between the Wang *et al.* (*9*) study and ours are very different; we interpreted our phylogenies and reconstructed evolutionary events manually, whereas they used gene-species tree reconciliations. Nevertheless, we believe that the decisive factor was the genomic database used. The only Thermoproteota members with Mtr in the Wang *et al.* (*9*) phylogenies were the Nezhaarchaeales. In that study’s MtrA phylogeny, these Nezhaarchaeales branched within a strongly supported Halobacteriota clade that included the o__ANME-1 and Methanoliparia. They obtained a similar topology with Nezhaarchaeales within Halobacteriota for MtrE. We would have also interpreted these trees as indicating a transfer from Halobacteriota to Nezhaarcheales and absence of Mtr in the ancestor of Thermoproteota. Consequently, this would suggest an origin of Mtr at the base of Euryarchaeota* and that the LMA was a methyl-dependent hydrogenotrophic methanogen.

In our more extensive genomic sampling, we additionally find Mtr in Methanomethylicales (*12*) and Bathyarchaeia (*37*, *38*) (in Bathyarchaeota* subgroups 20&22). This expands its distribution in Thermoproteota in accordance with (*12*, *37*) and establishes that Mtr was present at the origin of Thermoproteota, similar to Mcr. The genomic sampling probably also affects the Mtr tree topologies, as o__ANME-1 and Methanoliparia are not sister to the other Halobacteriota as in the Wang *et al.* (*9*) MtrA tree, neither for the MtrABCDEFG concatenation (Fig. 2B) nor for the MtrA single gene tree (fig. S1). Nevertheless, a recent analysis (*37*) suggested that the Mtr in Bathyarchaeia was horizontally acquired. In the single-gene phylogenies of MtrA (fig. S1) and MtrH (fig. S2), we find canonical MtrAH subunits of Thorarchaeia (belonging to Asgardarchaeota) branching close to Thermoproteota [see also (*37*)], as would be expected from a vertically inherited Mtr. However, many of these MtrA and MtrH relationships were not strongly supported. The Thorarchaeia were not included in the concatenated datasets, because they did not possess at least two non-MtrH subunits (see Materials and Methods). The Wang *et al.* (*9*) study also proposed that the different corrinoid methyltransferases of methyl-dependent hydrogenotrophic and methyl-dismutating methanogens date to the common ancestor of Euryarchaeota* and Thermoproteota. We tested this by constructing phylogenies of the methyltransferase subunits MtaB (methanol), MtsB (methanethiol), MtmB, MtbB, and MttB (mono-, di-, and trimethylamine). MtaB and MtsB were probably present at the ancestor of Euryarchaeota* (figs. S3 and S4); MtsB is the sister clade of MtaC [fig. S4 and (*13*)]. The methylamine methyltransferase phylogenies include numerous intra- and interdomain transfers, and many branches are poorly supported. The furthest back we can trace any of these methyltransferases is the ancestor of Euryarchaeota* (figs. S5 to S7), so they were probably not found in the LMA. Many of the extant methyl-dependent hydrogenotrophic methanogens have acquired MtaB independently through lateral transfer events; for instance, Methanomethylicales and Korarchaeia received it from Methanobacteria. For the other Euryarchaeota* lineages, the situation is less clear, but f__NM3 and Methanomassiliicoccales possibly acquired MtaB from within the Halobacteriota. We can conclude that methyl-dependent hydrogenotrophic methanogenesis emerged multiple times independently through a combination of vertical inheritance and transfers, as previously hypothesized (*11*, *12*). The key event was either a loss of Mtr (Methanofastidiosales, Methanomethylicales, and Korarchaeia) or an acquisition of Mcr (Methanonatronarchaeia and Methanomassiliicoccales). When we take the methyltransferases into account, the earliest point of origin for methyl-dependent hydrogenotrophic and methyl-dismutating methanogenesis is the ancestor of Euryarchaeota*. Note that CO_2_-reducing hydrogenotrophic methanogenesis can potentially be gained through an Mcr transfer as well, such as in Ca. Methanomixophus hydrogenotrophicum*. Nonetheless, we cannot know in this case whether Mcr was transferred to a non-methanogen like the Bathyarchaeia or it was a homologous recombination.

The previously unstudied evolutionary histories of Eha and Ehb corroborate the hydrogenotrophic capabilities of the LMA. The Eha genes form a highly conserved genomic cluster, and they have evolved mainly vertically with some lineage-specific modifications involving gain/loss of subunits or use of different ferredoxins (Fig. 3A and Supplementary Results and Discussion). The exceptions are a possible ancient homologous recombination event affecting some Methanobacteria and a transfer between o__JdFR-21 and Persephonarchaea* (MSBL1*) (Fig. 3A). Determining the direction of this transfer depends on where we place the roots of the reference and Eha phylogenies. Under the classic root (Fig. 1B) that is supported by outgroup-free rooting, Eha dates close to the base of Euryarchaeota*, after the divergence of Thermococci, and the Persephonarchaea* transferred it to o__JdFR-21. However, in that case, the Eha tree does not recover the expected monophyly of Halobacteriota with Methanomada*, so additional ancient transfers need to be assumed. In general, the position of Persephonarchaea* in the archaeal tree is problematic (*7*). Under the root of the archaeal phylogeny from Raymann *et al.* (*34*) (Fig. 3A, as displayed), o__JdFR-21 is in the Halobacteriota and has transferred Eha to Persephonarchaea*, and Eha dates to the LMA, or to the ancestor of Euryarchaeota* assuming our reference phylogeny (Fig. 1B). Because of the metabolic association of Eha with methanogenesis through the WLP, we tested whether the evolution of methyl and carbonyl branch components supports one of the two transfer directions. The carbonyl branch methyltransferase module (CdhDE; figs. S8 and S9) recovers the Persephonarchaea* to o__JdFR-21 transfer, while o__JdFR-21 have inherited other genes (for CdhB, Mch, and Mtd; figs. S10 to S12) vertically.

The evolution of Ehb (Fig. 3B) is more complicated than Eha. Beyond lineage-specific modifications, such as the loss of EhbKL in Theionarchaea*, the signal among subunits is inconsistent, resulting in different topologies that are rarely strongly supported, often affecting the position of Methanococci (data S8). The Ehb genes form a highly conserved cluster, except for Methanococci where the genes encoding subunits EhbEFGHIJKL and sometimes EhbMO are colocalized and separate from the rest. Furthermore, EhbHI in Methanococci are fused similar to Methanofastidiosales and Methanomethylicales. This is probably the result of a massive homologous recombination event related to Methanofastidiosales (figs. S13 to S15 and data S10; see Supplementary Results and Discussion for a detailed description). The presence of Ehb in the ancestor of Methanofastidiosales and Methanomethylicales further supports that these lineages were originally CO_2_-reducing hydrogenotrophic methanogens: (i) The Theionarchaea* lost Mcr and Mtr retaining the WLP and (ii) Methanofastidiosaceae, f__NM3, and some Methanomethylicales lost Mtr and the WLP becoming methyl-dependent hydrogenotrophic methanogens. Outgroup-free rooting (data S9) placed the root at Methanomethylicales, corresponding to a split between Euryarchaeota* and Thermoproteota and Ehb having been present in the LMA. This has interesting implications, for example, that the ancestor of Thermococcales lost methanogenesis and the WLP entirely, save for some remnant cofactor biosynthesis genes (*26*). Since Eha and Ehb form sister clades among [NiFe] group 4 hydrogenases (fig. S16) (*31*), this split and, by extension, Eha date to the LMA as well. Given that they both provide electrons to the initial reduction of CO_2_ to formylmethanofuran, they most probably arose from a duplication and subsequent modifications separating carbon fixation from methanogenesis in the LMA’s lifestyle.

The basal split between the canonical Mcr and the Acr homologs used in AAO led us to investigate whether the LMA also had AAO capabilities. To test this, we reconstructed ancestral sequences of Mcr, Mtr, Eha, and Ehb subunits for various possible roots (data S11). To account for bias introduced by taxa with missing subunits, we also reconstructed the concatenation phylogenies and ancestral sequences only using taxa with all subunits of the respective complexes. Root placement does affect the reconstructed sequences and, by extension, their highest similarities, but in general, these consist of CO_2_-reducing hydrogenotrophic and, more rarely, methyl-dependent hydrogenotrophic and methyl-dismutating methanogens (Methanobacteria, Methanococci, and Methanomethylicales in Ehb, and some Methanosarcinia) but no alkane oxidizers. For McrA, we also performed homology modeling of the ancestral sequences. The ancestral McrA exhibits the sequence conservation (*11*) of canonical McrA. In addition, the overall geometry of the methyl-CoM binding cavity is similar to that of canonical McrA in structural alignments of ancestral and extant sequences (fig. S17). It has been proposed that substitution of the large aromatic residues by smaller residues in the cavity in Acr is related to accommodating longer chain alkanes (*11*). Thus, it is unlikely that the LMA had any capacity for alkanotrophy, even if the Acr homologs were a basal divergence. However, the structural basis of alkane activation by Acr seems to be more complicated (*39*), and further analyses will be necessary.

Many of the putative markers outside of Mcr, Mtr, Eha, and Ehb have very narrow taxonomic distributions, so they emerged after the LMA and were not involved in the original CO_2_-reducing hydrogenotrophic methanogenesis. Some turned out to have no relation to methane metabolism. Among the putative markers from Gao and Gupta (*27*), there are seven genes found exclusively in Methanopyri and Methanobacteria. At least two of them (MK0750 and MK0751), based on synteny, are probably involved in pseudomurein biosynthesis (fig. S18). Another case is the Hcg proteins in the biosynthetic pathway of the iron guanylylpyridinol cofactor of the Hmd hydrogenase in Methanomada*, Methanomicrobia, and Desulfurobacteriaceae (fig. S19 and data S6 and S12). In single-gene phylogenies of the Borrel *et al.* (*11*) markers, many branches are not strongly supported (figs. S20 to S49). Nevertheless, from recovering the monophyly of individual lineages, we can trace the origin of individual markers to the LMA (m4-m23, m25, m26, and m37) or putatively to different points within the Euryarchaeota*. Finding the origin of a marker within Euryarchaeota, at least at the common ancestor of Methanomada* and Halobacteriota (m24, m32-m36, and m38), technically corresponds to the LMA under the Raymann *et al.* root (*34*).

### Widespread remnants of CO_2_-reducing hydrogenotrophic methanogenesis components

Together, the LMA possessed complete Mcr, Mtr, Eha, and Ehb complexes, along with multiple methanogenesis markers. Each of these complexes was then lost multiple times independently over the evolution of Archaea at various phylogenetic depths. Since Mcr is the marker for methane metabolism in general and Mtr for CO_2_-reducing hydrogenotrophic methanogenesis (again, disregarding acetoclastic, methyl-dismutating, and methoxyl-dismutating), finding a vertically inherited Mtr or a methanogenesis marker in a non-methanogenic lineage indicates that said protein is a remnant of the methane metabolism of one of its ancestors. Beyond methanogenesis, a wide presence of genomic remnants is known for the WLP that has also been independently lost over archaeal clades (*26*, *36*). The loss of the H_4_MPT branch and its auxiliary genes is often incomplete and tends to leave behind biosynthetic genes of the H_4_MPT and methanofuran cofactors, but also genes of the main pathway, such as Mch in Halobacteria (*26*). The same situation has been proposed to apply to methanogenesis markers (*11*) being repurposed into other pathways.

The most blatant example of a remnant is the complete Mtr complex found in Bathyarchaeia (o__B25, traditionally Bathyarchaeota* subgroups 20 and 22). Similarly, Thorarchaeia possess canonical MtrAH without the other subunits that could act as a methyltransferase in their proposed mixotrophic lifestyle (*40*). Concerning hydrogenases, Eha is a remnant in o__JdFR-21 or Persephonarchaea* and Ehb in Theionarchaea*. Eight markers, regardless of whether they date to the LMA or not, are present in non–methane-metabolizing lineages (m15-m18, m25, m26, m32, and m37). The following cases constitute remnants: m16 in Theionarchaea* (fig. S32), m26 in Thorarchaeia (fig. S42), m26 and m37 in the o__B25 Bathyarchaeia with Mtr (figs. S42 and S48), m32 in Hydrothermarchaeota (fig. S43), m15-m17, m25, and m32 in o__JdFR-21 (figs. S31 to S33, S41, and S43), and perhaps m25 in Lokiarchaeia (fig. S41) and m37 in Persephonarchaea* (fig. S48).

The lineages with methanogenesis remnants listed above retain the WLP (*7*, *26*). Moreover, AMO, AAO, and acetoclastic, methyl-dismutating, and methoxyl-dismutating methanogenesis seem to be derived metabolic capabilities. Thus, these lineages have most likely originated from ancestors that were CO_2_-reducing hydrogenotrophic methanogens. The link between the WLP and Mcr through Mtr persists in progressive intermediate loss stages in Bathyarchaeia and Thorarchaeia, making this inference more robust. Other than m26, which is tied to Mtr, we could neither identify reasons for the conservation discrepancies among markers and among lineages nor confidently infer the repurposed function of uncharacterized remnant markers from genomic context or otherwise. For our observations on the evolution and functional annotation of the markers, see Supplementary Results and Discussion.

### Evolutionary rates of membrane-bound complexes

In the membrane-associated complexes Eha, Ehb, and Mtr, many subunits belong to distinct protein families that lack any readily detectable relationship with other families. These could have emerged de novo at the LMA. Unlike generic hydrogenase subunits, they are exclusively associated with these complexes. To investigate how such subunits could have become established, we calculated the site-specific evolutionary rates of Mcr, Mtr, Eha, and Ehb subunits as an alternative to *dN*/*dS*, following Sydykova and Wilke (*41*). For Eha and Ehb, but not Mcr and Mtr, we found significant differences within each complex (Kruskal-Wallis, *P* = [1 × 10^−3^ to 2.2 × 10^−2^] for Eha and [2.3 × 10^−5^ to 5.6 × 10^−3^] for Ehb, depending on the rate calculation method; not significant for Eha Bayesian rates: *P* = 7.6 × 10^−2^). However, these differences were hard to pinpoint, since there were no subunits with consistently significantly different rates (fig. S50 and data S13). The exception was significantly lower Bayesian rates in the catalytic hydrogenase subunits of Ehb: EhbN and less so EhbM [Dunn’s test and/or pairwise Mann-Whitney, *q* (false discovery corrected *P* value) < 0.05] (fig. S50, J to L). Apart from a few outliers, the rates in all subunits are below one, although our using trimmed alignments probably excludes some more fast-evolving positions.

**Fig. 4.**
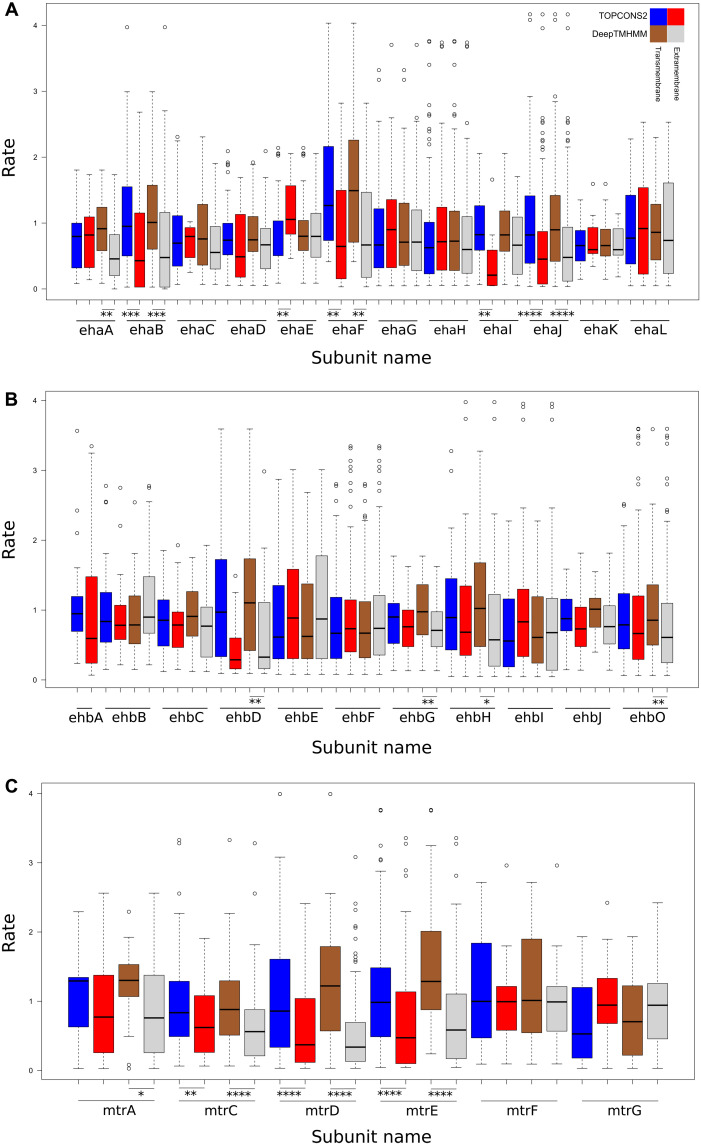
Site-specific rate comparison between membrane-bound and extramembrane residues of Eha, Ehb, and Mtr. Boxplots for site-specific empirical Bayesian rates calculated under Poisson+G16 for each predicted transmembrane subunit of (**A**) Eha, (**B**) Ehb, (**C**) Mtr, split between transmembrane and extramembrane residues as predicted by TOPCONS2 and DeepTMHMM. Asterisks denote statistical significance (*P < 5 × 10^−2^, **P < 1 × 10^−2^, ***P < 1 × 10^−3^, ****P < 1 × 10^−4^).

We then tested whether predicted transmembrane segments are more conserved compared to the extramembrane positions of the subunits. Our hypothesis was that the transmembrane regions would exhibit lower rates (*42*) due to being buried and/or in contact with other subunits and/or forming functional features [e.g., ion translocators in EhaHIJ (*29*), EhbF (*30*, *31*), MtrE, or MtrCDE (*43*)]. Nevertheless, there was no consistent significant rate difference between transmembrane and extramembrane residues for most subunits (Fig. 4). Where such a difference existed (Mann-Whitney, *P* = [6.2 × 10^−12^ to 3 × 10^−2^]), it was the extramembrane residues that had lower rates (exception: EhaE). Any correlations between a position’s predicted transmembrane probability and rate, even if significant, were weak to moderate (for the set of all subunits: Pearson *P* = [7.8 × 10^−12^ to 4.7 × 10^−2^], |r| ≤ 0.4; Spearman *P* = [2.3 × 10^−14^ to 4.4 × 10^−2^], |rho| ≤ 0.44; data S13), indicating that other structural features (solvent accessibility, flexibility, and packing) and/or functional conservation contribute to conservation in these complexes. Although they are not well documented (*42*), known examples of high conservation in extramembrane residues due to ion selectivity and translocation exist (*44*), which might also apply for Eha, Ehb, and Mtr.

### Subsurface lineages with methanogenesis remnants perform mixotrophic metabolisms

**Fig. 5.**
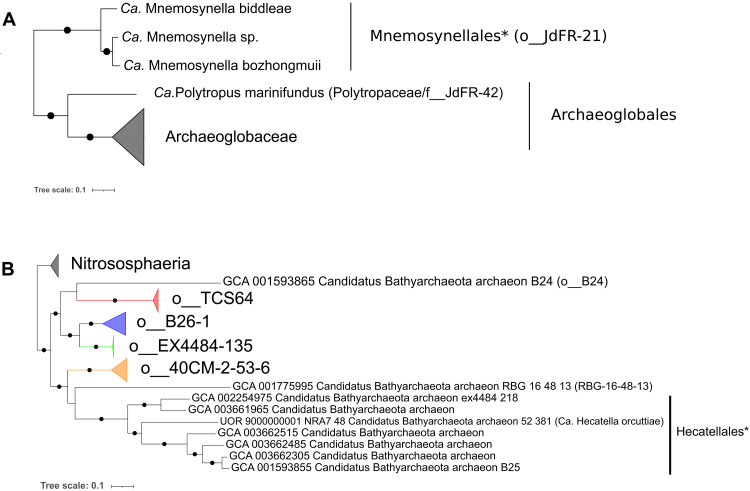
Phylogenetic positions of Mnemosynellales and Hecatellales. ML phylogenies of (**A**) Mnemosynellales* within Archaeoglobi (6021 amino acid positions) and (**B**) Hecatellales* in Bathyarchaeia rooted with Nitrososphaeria (7154 amino acid positions) based on the supermatrix of 36 Phylosift markers. Tree (A) is the expanded Archaeoglobi clade from Fig. 1B. Black circles indicate strongly supported branches (ultrafast bootstrap ≥ 95, aLRT SH-like ≥ 80).

**Fig. 6.**
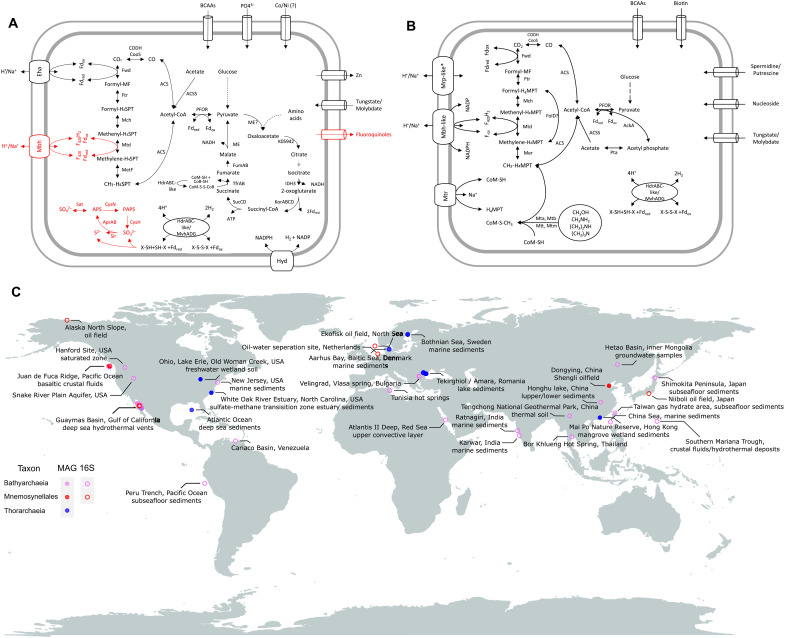
Metabolism and biogeography of Mnemosynellales and Hecatellales. Metabolic reconstructions for (**A**) Ca. Mnemosynella biddleae* and Ca. Mnemosynella bozhongmuii* and (**B**) Ca. Hecatella orcuttiae*. Systems marked in red are found exclusively in Ca. M. biddleae*, perhaps due to the higher quality and size of the genome. MF, methanofuran; H_4_MPT, tetrahydromethanopterin. (**C**) Biogeographic distribution of Mnemosynellales*, Hecatellales*, and Thorarchaeia with canonical MtrA. Coordinates/location and environment type were recovered from the respective whole-genome sequencing (WGS) project metadata in NCBI and 16*S* rRNA gene entries in SILVA.

The National Center for Biotechnology Information (NCBI) taxon “Euryarchaeota archaeon JdFR-21” possesses five remnant methanogenesis markers, more than any other nonmethane/alkane-metabolizing archaeon. JdFR-21 is a member of the NRA7* clade (o__JdFR-21) in Archaeoglobi and was recovered from subsurface fluid metagenomes of the Juan de Fuca Ridge (*45*), like the alkane oxidizer Ca. Polytropus marinifundus* (*24*) (f__JdFR-42). The JdFR metagenomes also contain JdFR-11, one of the Bathyarchaeia with canonical Mtr. With cursory BLAST (*46*) searches, we found two additional NRA7* MAGs (Archaeoglobi MAG-15 and Archaeoglobi MAG-16) from the Shengli oil field metagenomes (*47*) that were submitted to NCBI after we created our local genomic databases and were thus not included in other phylogenomic analyses. We downloaded the JdFR and Shengli metagenomic reads from the NCBI Sequence Read Archive (SRA), reassembled and rebinned them, and manually curated the genomes, improving upon their NCBI counterparts. The refined bins corresponding to JdFR-21, MAG-16, and JdFR-11 are near high-quality genomes as per the minimum information about a metagenome-assembled genome (MIMAG) standards (table S2) (*48*). We then determined their taxonomic placement trying to account for various sources of bias in the phylogenies (data S14). The two JdFR MAGs are the highest-quality representatives of their respective order-level lineages (Fig. 5 and table S2). The taxonomic delineation was corroborated by pairwise Average Nucleotide Identity (ANI) and Average Amino acid Identity (AAI) comparisons (figs. S52 and S53). Last, we performed an in-depth annotation of their metabolisms to determine how they use their methanogenesis remnants (Fig. 6, A and B, and data S15). We followed the same process for the JdFR Hydrothermarchaeota (JdFR-16, 17, and 18) and Geothermarchaeales genomes (JdFR-13 and 14) (fig. S51 and data S15) to improve the archaeal community context of JdFR-21 and JdFR-11 metabolism. For the species with mid- to high-quality draft genomes, we propose the following names: Ca. Mnemosynella biddleae* for JdFR-21, Ca. Mnemosynella bozhongmuii* for MAG-16 (order: Mnemosynellales*), Ca. Hecatella orcuttiae* (order: Hecatellales*) for JdFR-11, Ca. Pyrohabitans jungbluthii* for JdFR-16, Ca. Scotarchaeum otlingeri* for JdFR-13, and Ca. Geothermarchaeum rappei* for JdFR-14 (order: Geothermarchaeales). JdFR-18 has already been named Ca. Hydrothermarchaeum profundi in (*49*), and JdFR-17 falls under the genus Pyrohabitans*, but its genome quality is quite lower (fig. S54). Full genome statistics for all MAGs binned in this study are given in table S2. Together with our metabolic reconstructions and other ecological descriptions given below and in Supplementary Results and Discussion, we fulfil the criteria for naming these species and their corresponding higher-level taxa as per Murray *et al.* (*50*).

The carbon cycling capabilities of Mnemosynellales* (Fig. 6A) revolve around the WLP, but defining whether the pathway runs oxidatively or reductively is problematic. Mnemosynellales* can oxidize acetate and possess a Hyd-like hydrogenase for hydrogen evolution, but they also encode Eha that so far is only known to provide electrons to reductive reactions of the H_4_MPT branch. Mnemosynellales* genomes encode an Hdr/Mvh-like complex that could function on CoM-CoB or other heterodisulfides. The Ca. M. biddleae* genome also contains genes for an Mbh/Mrp-like hydrogenase and both assimilatory and dissimilatory sulfur metabolism, where Hdr/Mvh could perform thiosulfate or other heterodisulfide disproportionation. Thus, the WLP could run either reductively in hydrogenotrophic or sulfur-oxidizing carbon fixation, or oxidatively coupled to sulfate reduction as in many other Archaeoglobi. Mnemosynellales* appear capable of performing most tricarboxylic acid (TCA) cycle reactions other than the steps from malate to oxaloacetate and citrate to isocitrate. The succinate to fumarate conversion is predicted to be catalyzed by the CoM- and CoB-forming thiol:fumarate reductase that is syntenic to an Hdr-like (heterodisulfide reductase) complex and Eha. Such fumarate reductases have been proposed to function in some Asgardarchaeota lineages (*51*). The CoM-CoB heterodisulfidic bond could be regenerated by the Hdr-like complex, with a reductive WLP functioning as an electron sink. The underlying assumption here is a source of oxaloacetate, perhaps from amino acid fermentation or from pyruvate through the oxaloacetate-decarboxylating malate dehydrogenase, because pyruvate carboxylase was not found. Nonetheless, it is possible that the TCA reactions run in the opposite direction through reducing potential from hydrogen or sulfur species. Unfortunately, we were unable to predict the role of the five methanogenesis markers.

Hecatellales* include the B25 MAG that has been proposed to be an acetogen (*38*). Ca. H. orcuttiae* (Fig. 6B) seems to have the capacity for not only acetogenesis running the WLP reductively but also acetate assimilation and transferring methyl moieties from methanol and methylamines into an oxidative WLP through Mtr. The Bathyarchaeia member CR_14* (not in our datasets) branches within o__B26-1 and contains a complete canonical Mtr that has also been suggested to link methylated compounds to the WLP (*52*). The presence of Mtr outside Hecatellales* further consolidates our inference of ancestral CO_2_-reducing hydrogenotrophic methanogenesis in Bathyarchaeia. Membrane potential is probably generated by an Mbh/Mrp-like hydrogenase regulated by an additional Mrp antiporter that is syntenic to the formylmethanofuran dehydrogenase Fwd. Ca. H. orcuttiae* might perform hydrogen-dependent heterodisulfide disproportionation via an Hdr/Mvh-like complex, similar to Mnemosynellales*.

In terms of biogeography (Fig. 6C, fig. S55, table S3, and data S14), Ca. Mnemosynella* is the only known genus in its order and is found globally in oil fields. It includes a divergent geothermal clade found exclusively in the eastern Pacific, but the branch supports in the 16*S* phylogeny are inadequate to determine its origin (fig. S55A). Hecatellales* MAGs have only been recovered from geothermal environments in the eastern Pacific, but from their 16*S* ribosomal RNA (rRNA) gene sequences, we can deduce that they are present in many types of mainly high-temperature environments around the world, into which metagenome sequencing efforts should be expanded (fig. S55B). By contrast, the Thorarchaeia MAGs that use the canonical MtrA originate from a wide variety of anaerobic environments and localities. Because of the diversity of how methanogenesis remnants have been integrated in metabolism around the WLP, Mnemosynellales*, Hecatellales*, and Thorarchaeia can occupy multiple niches across diverse environments in the global carbon cycle.

To summarize, the ancestor of non-DPANN* Archaea (and perhaps all Archaea) was a carbon fixing CO_2_-reducing hydrogenotrophic methanogen but not an alkane oxidizer, not unlike the extant Methanomada*. Methyl-dependent hydrogenotrophic methanogenesis has arisen from CO_2_-reducing hydrogenotrophic methanogenesis multiple times in unrelated clades due to losses of Mtr linking methanogenesis with the WLP or acquisition of Mcr combined with vertical or horizontal inheritance of corrinoid methyltransferases. The loss of CO_2_-reducing hydrogenotrophic methanogenesis across archaeal lineages has been far from a straightforward process, leaving behind genomic remnants. Mtr and the hydrogenases Eha and Ehb are prime examples of such remnants. These large complexes are unconventional examples where exposed residues evolve slower than transmembrane buried ones, and the selective pressures acting on them warrant extensive study. The presence of methanogenesis remnants in various lineages has created intermediate metabolic states that are centered around the WLP and result mainly in various forms of mixotrophy. The lineages that possess them, such as Mnemosynellales*, Hecatellales*, and Thorarchaeia, thus occupy diverse niches in anaerobic carbon cycling.

## MATERIALS AND METHODS

### DUF distribution

We determined the taxonomic distribution of 4049 DUFs and uncharacterized protein families (UPFs) from Pfam release 32.0 with a custom script (distributions_uniprot.py; data S16) against a local copy of UniProt (release 2019_07). For families where no distribution was available, because of lack of cross-references to Pfam, we estimated the distribution from that family’s Pfam “Species” tab. Families with at least 50% Archaea in their distribution were retained for downstream analyses as archaeal DUFs.

### Homology searches

For initial homology searches, we used HMMER 3.2.1 (*53*) with a cutoff of 1 × 10^−5^ against local databases of 1808 archaeal and 25,118 bacterial genomes. The Hidden Markov Model (HMM) profiles were retrieved preferably from Pfam (*54*) or, if one could not be retrieved, from eggNOG’s arCOGs (*55*). For the 155 DUFs and genes from Gao and Gupta (*27*), we also searched against local databases of 1611 Eukaryotes and 14,494 viruses with the same parameters. The process for picking genomes for the local databases is explained in Supplementary Methods. Because of only getting hits of dubious quality, all eukaryotic sequences were ultimately removed. For searches that produced too many hits (as a rule of thumb >1000), we performed a new homology search using DIAMOND v0.9.24.125 (*56*) (blastp -e 1e-5 --more-sensitive -k 1000) with a single query sequence.

### Alignment and single-gene phylogenies

We aligned all datasets with MUSCLE v3.8 (*57*). Then, we manually curated the alignments to remove distant and/or poorly aligning homologs and fuse contiguous fragmented sequences with a custom script (fuse_sequences.py; data S16) and realigned them. Last, we trimmed the alignments with BMGE version 1.12 (*58*) (BLOSUM30).

We reconstructed all single-gene phylogenies in IQ-Tree 2 (*59*) (for the exact version of IQ-Tree, see the log files in Supplementary Data) under the model automatically selected by Modelfinder (*60*) (-m MFP). We calculated branch supports with 1000 ultrafast bootstrap (*61*) and 1000 aLRT SH-like (*62*) replicates, and the approximate Bayes test (*63*) (-bb 1000 -alrt 1000 -abayes). We visualized all phylogenies in iTOL v6 (*64*). The evolutionary history of individual genes (or concatenated gene sets; see below) was determined through a basic phylogenomic approach, where the gene trees were manually compared with the assumed vertical inheritance in the species/reference tree (Fig. 1B) to reconstruct duplication, loss, and transfer events.

### Mcr, Mtr, Eha, Ehb, and Hcg concatenation phylogenies

To increase the signal of Mcr, Mtr, Eha, Ehb, and Hcg sequences, we constructed a series of concatenated alignment phylogenies with taxa that possessed at least two proteins of the respective complex/pathway. Specifically, we concatenated McrABG [McrCD were among the 38 methanogenesis markers of Borrel *et al.* (*11*) but are generally not used in the literature], MtrABCDEFG (MtrH was problematic for reasons detailed above), and EhbABCDEFGHIJKLMNOP. For Eha, we included subunits EhaBCDEFGHJLMNO. In the Hcg genes, we noticed strongly supported incongruences already in the single-gene trees that were reflected in gene colocalization (fig. S19 and data S6 and S12), so we created two concatenations: HcgAEFG and HcgBC.

We inferred single-gene maximum likelihood (ML) phylogenies in IQ-Tree 2 with the trimmed alignments (as above) of the proteins in each concatenation under the model predicted by Modelfinder with 100 bootstrap replicates (-b 100). We collapsed nodes with support below 80% with TreeCollapseCL 4 (http://emmahodcroft.com/TreeCollapseCL.html). We tested these trees for congruence against the concatenation tree using the internode certainty test (*65*) in RaxML v8.2.11 (*66*). We removed any incongruent sequences from their respective subunits and repeated the process until no further incongruence could be detected. The only exception was the Methanococci+Methanobacteria clade of Mcr, where despite our best efforts we could not detect the source of incongruence and ultimately disregarded it, as we did not consider it to affect the overall topology. Ultimately, we only ended up removing the Methanopyri from McrA (2 sequences) and f__NM3 (1 sequence) from EhbN.

For Ehb, the position of Methanococci was inconsistent among subunits, and synteny in this lineage was far less conserved than other clades. However, there were no (strongly supported) incongruences in the single subunit phylogenies. Thus, to explore potential homologous recombination events, we constructed additional phylogenies for subsets of the Ehb subunits (EhbEFGHIKLMO and EhbEGHIKLM). Detailed explanations for the rationale behind the subunit choices for the concatenations of Eha, Ehb, and Hcg are given in Supplementary Methods.

For the final concatenated datasets, we ran phylogenies in IQ-Tree 2 under the same parameters as single-gene trees above and then used these as guide trees to infer phylogenies under the LG+C60+F+G model with the posterior mean site frequency (PMSF) approximation (*67*). Branch supports were calculated with 1000 ultrafast bootstrap and 1000 aLRT SH-like replicates, and the approximate Bayes test. For all synteny comparisons in the main manuscript figures, we used GeneSpy 1.1 (*68*).

### Outgroup-free rooting and rootstraps

For all the Mcr, Mtr, Eha, Ehb, and Hcg concatenations described above, we performed outgroup-free rooting with the MAD 2.2 (*69*) and MinVar v1.5 (*70*) methods on phylogenies under the LG+C60+F+G model and 100 bootstrap replicates (-b 100) (PMSF approximation as above). Rooted phylogenies were also inferred under the NONREV protein model (*59*) with 100 bootstrap replicates. The sets of rooted phylogenies and bootstrap trees were used to calculate rootstrap supports (*71*). We also rooted the single-gene methyltransferase (MtaB, MtsB, MtmB, MtbB, and MttB) phylogenies with MAD and MinVar.

### Gene and site concordance factors

We calculated gene and site concordance factors (gCF and sCF) (*72*) for Mcr, Mtr, Eha, and Ehb using the mixture model phylogenies as species trees, the subunit single-gene phylogenies (with incongruences resolved) as gene trees, and the concatenated alignments as input alignments. To isolate the effect of individual subunits on the signal, we also calculated sCF with the mixture model phylogenies as species trees but in a series of separate runs with each subunit as the input alignment.

### Ancestral sequence reconstructions

We reconstructed ancestral sequences via the empirical Bayesian method in IQ-Tree 2 (-asr) for all nodes and all concatenated subunits of Mcr, Mtr, Eha, and Ehb in two ways. First, we used the concatenation phylogenies constructed previously for each complex under the LG+C60+F+G model but substituted the concatenation of trimmed alignments with their untrimmed equivalents for the reconstruction. We parsed the ancestral sequence reconstruction (ASR) output with a custom script (ASR_parser.py; data S16) that separates the sequences of individual subunits and calculates the mean posterior probability for the reconstructed sequence of each node. These reconstructed sequences consist of the residue with the highest probability for each site. The mean posterior probabilities are gross underestimates, because IQ-Tree does not reconstruct indels, and thus, the probability for sites with many gaps ends up being very low. Our second approach to ASR was almost identical. However, this time, we reduced the datasets for each complex to only include taxa that had a complete complex to avoid including large gaps in the concatenation that could affect the reconstruction. If a subunit was missing in entire clades of the phylogeny, we either omitted that subunit (EhaL and EhbKLN) or these taxa in the case of Methanopyri in Mcr where we had only three subunits. We then inferred phylogenies with automatic model selection (-m MFP) and used them as guide trees for LG+C60+F+G phylogenies (PMSF approximation), reconstructing ancestral sequences in tandem.

We retroactively added indels to the reconstructed sequences by a consensus-like approach. For each subunit, the reconstructed sequences corresponding to potential LMA nodes from both approaches were added to their respective datasets of taxa with complete complexes. These were realigned and trimmed with Clipkit v0.1.2 (*73*) (-m gappy -g 0.5) to remove positions with at least 50% gaps. Because of their missing clades, EhaL and EhbKLN were omitted from indel inference. Last, we performed homology modeling and structural alignments of the ancestral McrA sequences (see Supplementary Methods).

### Site rate estimation and transmembrane segment prediction

We estimated empirical Bayesian and ML site-specific rates for all Mcr, Mtr, Eha, and Ehb subunits as above, from their trimmed alignments (before congruence testing) and respective single-gene phylogenies, after benchmarking the effect of model choice on such shorter alignments (see Supplementary Methods). We tested whether any subunits within each complex had significantly higher or lower rates through a Kruskal-Wallis test followed by Dunn’s test and a series of Mann-Whitney *U* tests for all subunit pairs of each complex (both with Benjamini-Hochberg correction).

We calculated the transmembrane per site probability for each subunit both numerically with the Python implementation (https://github.com/dansondergaard/tmhmm.py) of TMHMM2.0 (*74*) and on the Polyphobius server (*75*) and as a structural feature on the TOPCONS2 server (*76*) and with DeepTMHMM 0.0.31 (https://biolib.com/DTU/DeepTMHMM). The reason for this was to account for uncertainties due to differences among algorithms and the fact that we used *Methanothermobacter marburgensis* sequences from the trimmed alignments as input (i.e., a single sequence per subunit). For all subunits with predicted transmembrane segments in TOPCONS2, we calculated Spearman and Pearson correlations between empirical Bayesian rates under Poisson+G16 and the transmembrane helix probability from TMHMM2.0 and Polyphobius. We also ran the Mann-Whitney test to compare the populations of rates between positions that were predicted as transmembrane helices and those that were not (i.e., extramembrane) in TOPCONS2 and DeepTMHMM.

### Targeted reconstruction of genomes from the Juan de Fuca Ridge and Shengli metagenomes

We retrieved publicly available reads of metagenomes that contained the target organisms from division NRA7* (o__JdFR-21) and Bathyarchaeia (assembly accessions; JdFR-20: GCA_002011155, JdFR-21: GCA_002011165, JdFR-10: GCA_002009985, JdFR-11: GCA_002011035, MAG-15: GCA_014361185, and MAG-16: GCA_014361165) from SRA (JdFR: SRR3723048 and SRR3732688; Shengli: SRR11866725, SRR11866724, and SRR11866717). We then quality-filtered the reads, assembled them, binned the genomes, and curated them. For the full procedure, see Supplementary Methods.

### Mnemosynellales* and Bathyarchaeia taxonomy and phylogenomics

As per their GTDB (*2*) classification, the three Mnemosynella* species (Ca. M. biddleae*/JdFR-20,21, Ca. M. sp.*/MAG-15, and Ca. M. bozhongmuii*/MAG-16) and Ca. H. orcuttiae*/JdFR-10,11 are members of order-level lineages in Archaeoglobi and Bathyarchaeia, respectively. Because of their higher quality and inclusion in our local genomic databases after the dereplication, we refer to JdFR-21 and JdFR-11 throughout this text. We performed an in-depth exploration of the phylogenetic position of Mnemosynellales* in Archaea and Hecatellales* in Bathyarchaeia. For the detailed analyses used for the phylogenomic placement, taxonomic delineation with ANI and AAI, and determining the environmental and biogeographic (with 16*S* phylogenies) distribution of Mnemosynellales* and Hecatellales*, see Supplementary Methods.

### Metabolic reconstructions

The metabolic potentials of Ca. M. biddleae*, Ca. M. bozhongmuii*, Ca. H. orcuttiae*, Ca. Hydrothermarchaeum profundi, Ca. Pyrohabitans jungbluthii*, Ca. Scotarchaeum otlingeri*, and Ca. Geothermarchaeum rappei* were predicted with BlastKOALA version 2.2 (*77*) using their respective taxids from NCBI and searching against the species_prokaryotes database. Additional annotations were produced with HydDB (*78*), dbCAN2 V3.0.1 (*79*) (dbCAN meta server with all options enabled), and MEROPS release 12.4 (*80*) (searched locally with DIAMOND blastp; cutoff, 1 × 10^−5^).

### Statistical analyses

See Supplementary Methods.

## Supplementary Materials

### This PDF file includes:

Supplementary Text Figs. S1 to S55 Tables S1 to S3 References

## Notes

View/request a protocol for this paper from *Bio-protocol*.
