# Supplementary material for: Genomic remnants of ancestral methanogenesis and hydrogenotrophy in Archaea drive anaerobic carbon cycling
Source: Sci Adv. 2022 Nov 4;8(44):eabm9651. doi: 10.1126/sciadv.abm9651 (PMC9635834; doi:10.1126/sciadv.abm9651)
Supplement: Supplementary file 1 — Supplementary Text Figs. S1 to S55 Tables S1 to S3 References [file sciadv.abm9651_sm.pdf]

Science Advances
AAAS

Supplementary Materials for

Genomic remnants of ancestral methanogenesis and hydrogenotrophy in Archaea drive anaerobic carbon cycling

Panagiotis S. Adam *et al.*

Corresponding author: Panagiotis S. Adam, panagiotis.adam@uni-due.de

Sci. Adv. **8**, eabm9651 (2022)
DOI: 10.1126/sciadv.abm9651

This PDF file includes:

Supplementary Text
Figs. S1 to S55
Tables S1 to S3
References

Supplementary Text

Supplementary Methods

Genome selection for local databases

For Archaea and Bacteria, we used all entries on NCBI as of 2019.06.01 dereplicated at species level (99% rpS3 amino acid identity clustering(*81*) with CD-HIT version 4.8.1(*82*) followed by 85% Mash and 95% gANI in dRep version 2.2.3(*83*)). The eukaryotic and viral genomes were picked through NCBI's Taxonomy IDs: one genome per species ID, selecting in order of preference the first reference, representative, complete, scaffold, or contig completion level genome.

Detailed description of homology search methods for Eha, Ehb, Hcg

Since several of the proteins in the Eha, Ehb, and Hcg sets were either DUFs or part of the lists from Gao & Gupta(*27*), we could compare the single gene distributions and phylogenies to determine potential issues in the homology searches for the more problematic (poorly annotated, fast evolving etc.) proteins in each complex or pathway. HMM searches often produced >1000 hits for most subunits and thus a lot of computational power, time, and manual work would be required to isolate our homologs of interest. For that reason, we instead searched for homologs using DIAMOND blastp with two query sequences on the taxonomic extremes of each complex or pathway. For Eha, the queries were homologs from *Methanothermobacter marburgensis* (Methanobacteria) and *Methanolacinia petrolearia* (Methanomicrobia), for Ehb from *M. marburgensis* and *Ca.* Methanosuratus petracarbonis* (Methanosuratus petracarbonis_A, Methanomethylicales), and for Hcg from *M. marburgensis* and *Desulfurobacterium thermolithotrophum* (Desulfurobacteriaceae). *M. marburgensis* was used as a reference for picking the first query using information from (*28*). Other known homologs from the literature in *Methanococcus maripaludis, Methanocaldococcus jannaschii, Methanothermobacter thermoautotrophicus, Methanopyrus kandleri* were used as (additional) queries in some cases. When entire clades were missing partially or entirely among the homologs, the HMM profiles or DIAMOND set of queries were expanded by using all hits of the previous search round to create a new HMM profile or collection of queries and rerun the search. To clean up the datasets and retain our homologs of interest, we first aligned the initial pool of hits with MUSCLE(*57*). Then in Seaview(*84*) we manually inspected the alignments, constructed preliminary phylogenies with BioNJ(*85*) (Poisson or Observed distances) and/or PhyML(*62*) (default options, no topology optimization), and isolated (monophyletic) clades containing our homologs of interest. None of these initial phylogenies were retained, since they were made on a trial-and-error basis, but they can be reproduced through the datasets in the Supplementary Data files. We confirmed suspected gene losses by manually comparing the hits of the homology searches with the synteny in each taxon. Below we list for each gene whether we used the base queries for a DIAMOND search and if it was found in this study as a DUF, or in Gao & Gupta(*27*) with the corresponding Pfam or arCOG accession for the HMM profile. We also detail any deviations in the homology search methodology.

Eha

EhaA: (Gao & Gupta, 2007), PF17367, custom HMM from all the hits of the original search

EhaB: (Gao & Gupta, 2007), arCOG04828

EhaC: DUF2109

EhaD: DUF2108

EhaE: DUF2107

EhaF: DUF2106

EhaG: DUF2105

EhaH: PF10125, found from synteny

EhaI: (Gao & Gupta, 2007) contained only arCOG05034. From Uniprot cross-references for multiple sequences, we found HMM profiles for arCOG05034 and arCOG06464 and pooled the

hits together. Mnemosynellales*, Persephonarchaea*, and *M. kandleri* were not found through HMM searches, so we added their sequences manually from expected synteny and BLAST searches against NCBI. We pooled all these sequences for recursive DIAMOND searches, using the hits from the previous search as queries. We stopped after 3 rounds of searches, since the number of hits decreased afterwards.
EhaJ: base queries
EhaK: (Gao & Gupta, 2007) contained only arCOG08277. From Uniprot cross-refs, found HMM profiles for arCOG06676, arCOG08277, arCOG10247, arCOG60928, and proceeded as with EhaI. Only 1 round of DIAMOND searches was necessary, as we found no further hits afterwards.
EhaL: DUF2104, custom HMM from all the hits of the original search.
EhaM: DUF1959, custom HMM from all the hits of the original search.
EhaN: base queries
EhaO: base queries
EhaP & EhaQ: In *M. kandleri*, the gene called EhaP in Uniprot (AAM01675.1) is an unrelated ferredoxin, distantly related to RnfB homologs in Firmicutes* but also to its own EhaR* (AAM01674.1). The real EhaP homolog in *M. kandleri* is called EhaQ (AAM01677.1). However, when running BLASTp against NCBI non-redundant with the *M. marburgensis* EhaQ, the first *M. kandleri* hit at twilight zone homology is AAM01676.1 listed in Uniprot as EhbK (marked with an asterisk in Fig. 3A). Using *M. maripaludis* as the query, the first *M. kandleri* hit is the ferredoxin AAM01674.1. We did not find either of these sequences in our local DIAMOND searches, even if they could be considered as EhaQ-like. Many Methanococci also carry an EhaQ-like homolog that was not picked up in our homology searches but is part of the Eha cluster (AAB98510.1 in *M. jannaschii*, marked with an asterisk in Figure 3a).
EhaP: base queries, plus MSBL1 archaeon SCGC-AAA382A20
EhaQ: *M. marburgensis* and *M. maripaludis*
EhaR: The gene called EhaR in *M. marburgensis* (ADL58396.1) was annotated as a ribokinase and only picked hits in Methanobacteria. The one called EhaR in *M. maripaludis* based on Uniprot and synteny (ABO34430.1) and its closest homolog in *M. kandleri* (AAM01674.1) did have some hits in Methanobacteria. These included SCG85576.1 in *Methanobacterium congolense*, and when NCBI non-redundant was queried, ADL59095.1 (MvhB) in *M. marburgensis*. However, the syntenic polyferredoxin ADL58399.1, as well as SCG85576.1, and ADL59095.1, when used in a BLASTp search in the same way, pick up AAM01674.1 as their first hit in *M. kandleri*. The final query sequences for EhaR originated from *M. maripaludis* (ABO34430.1), *M. kandleri* (AAM01674.1), and *M. marburgensis* (ADL58399.1).
EhaS: base queries, DIAMOND with all hits of the first search.
EhaT: base queries, DIAMOND with all hits of the first search
<u>Ehb</u>
EhbA: base queries
EhbB: arCOG04878
EhbC: (Gao & Gupta, 2007), arCOG04877
EhbD: base queries
EhbE: base queries
EhbF: base queries
EhbG: (Gao & Gupta, 2007), arCOG05076
EhbH: base queries
EhbI: base queries
EhbJ: custom HMM profile from pooled hits of arCOG06490 and arCOG06683
EhbK: base queries, plus *M. maripaludis*
EhbL: base queries
EhbM: base queries

EhbN: base queries
EhbO: base queries
EhbP: (Gao & Gupta, 2007), PF10622
Hcg
HcgA: base queries
HcgB: DUF3236
HcgC: DUF1188
HcgD: absent in *D. thermolithotrophum,* queries were *M. marburgensis* and *M. kandleri*
HcgE: (Gao & Gupta, 2007), arCOG01677
HcgF: UPF0254
HcgG: (Gao & Gupta, 2007), PF10113

Subunit omissions from Eha, Ehb, and Hcg concatenations

As mentioned in the Methods section, certain subunits were omitted from the concatenations of Eha, Ehb, and Hcg. The specific reasons for each subunit are summarized below.
EhaA: Missing in Methanomicrobia; the phylogeny did not even recover the monophyly of the other clades (Persephonarchaea* and Methanopyri inside Methanobacteria at different branches, Mnemosynellales* inside Methanococci).
EhaI: Issues in the homology searches. Very short gene with phylogeny topology that made it problematic for use in a concatenation (Persephonarchaea* and Methanococci inside Methanobacteria).
EhaK: Issues in the homology searches. Very short gene with phylogeny topology that made it problematic for use in a concatenation (Methanopyri inside Methanobacteria, Methanococci as grades at the base of Methanomada*, Persephonarchaea* and Mnemosynellales* as grades at the base of Methanomicrobia).
EhaP: Ferredoxin with very low conservation among clades and poorly aligning. Issues with the annotations and homology searches (i.e., establishing orthology).
EhaQ: Ferredoxin with very limited distribution (Methanobacteria, Methanococci). Issues with the annotations and homology searches (i.e., establishing orthology).
EhaR: Limited distribution (Methanobacteria, Methanococci, Methanopyri). Issues with the annotations and homology searches (i.e., establishing orthology).
EhaS: Paralog of Ftr whose distribution does not match the other subunits(*26*). Only part of the Eha cluster in Methanobacteria.
EhaT: Very limited distribution (only Methanobacteria).
For EhaAIK their short length makes their omission probably inconsequential. In general, we did not wish to risk the overall concatenation signal by introducing multiple poorly conserved subunits or with dubious orthology. Similar to EhaA, EhaC also had a slightly problematic topology but was not as pronounced (Methanococci and Methanopyri respectively monophyletic but inside Methanobacteria).
EhbJ: Only omitted in the Methanococci cluster concatenations. Phylogeny topology that made it problematic for use in a concatenation (Methanofastidiosaceae and Theionarchaea* at two separate branches within Methanobacteria). A similar monophyly issue existed in EhbH (either Methanomethylicales or Methanococci+Methanofastidiosales within Methanobacteria, depending on the root position, but monophyletic among themselves) but it was less pronounced in our opinion, so that subunit was kept in the concatenations.
HcgD: Absent in Desulfurobacteriaceae; there was no monophyletic clade for the other taxa in the preliminary phylogenies.

McrA homology modeling

We performed all homology modeling on the Phyre2 server(*86*) with the intensive mode. For all visualization and structural alignments, we used Pymol v2.4(*87*) and its alignment plugin, aligning each homology model to the best template picked by Phyre2 (all to one, defaults). All RMSDs were <0.4 Å.

Site rate estimation benchmarks

To test the effect of model choice on Mcr, Mtr, Eha, and Ehb subunit site rates, we calculated Pearson and Spearman correlation coefficients between ML and empirical Bayesian rates under Poisson+G16, and between ML rates under Poisson and Poisson+G16. The Poisson (JC-like) model was selected based on the literature(*88*, *89*). The Spearman correlations were almost perfect (Spearman rho >0.99, except for EhaS=0.70 for Bayesian against ML and EhaS=0.68 for ML against ML, p≤2.2E-16) but dropped very often to moderate for Pearson correlations (Pearson r >0.42, p≤4.7E-4). The reason is that short alignments created unrealistic outlier values in ML rates when rate heterogeneity was included in the model.

Metagenome assembly and binning

For the JdFR and Shengli reads, we quality filtered them using BBDuk (https://sourceforge.net/projects/bbtools/) and Sickle version 1.33(*90*). We assembled the reads using metaSPADES v3.14.1(*91*). We assembled the JdFR metagenomes individually, while the Shengli metagenomes were co-assembled, as in the original publication(*47*). Then, we processed both the JdFR and Shengli metagenomes identically using the uBin helper scripts(*92*). We performed automated binning using ABAWACA v1.07(*93*) with 3000/5000 and 5000/10000 as minimum/maximum fragment scaffold size parameters, respectively. We performed additional automated binning with MaxBin2 v2.2.4(*94*) and both available marker sets encompassing 40 or 107 marker genes, respectively, were employed. We consolidated the resulting four sets of bins in DASTool version 1.1.0(*81*). We identified the target bins through each organism's rpS3 sequence in Genbank and then curated them in uBin v0.9.14 using GC, coverage, and taxonomy(*92*), supervised by 38 universal archaeal marker genes(*95*). Since they possessed at least one methanogenesis marker, we also recovered genomes of two Geothermarchaeales (JdFR-13: GCA_002011075, JdFR-14: GCA_002011085) and three Hydrothermarchaeota (JdFR-16: GCA_002010065, JdFR-17: GCA_002011115, JdFR-18/*Ca.* Hydrothermarchaeum profundi: GCA_002011125) in the same manner. We estimated genome quality with CheckM v1.1.3(*96*), based on which we manually picked one type genome for each species. All our bins were improvements on the ones already submitted to NCBI, except perhaps JdFR-13 that contained more contigs/scaffolds but a higher N50. We ran Prokka v1.14.0(*97*) (--kingdom Archaea --compliant) on all bins to predict Open Reading Frames. The Prokka output was then used to manually detect the presence of rRNA and tRNA genes.

Mnemosynellales* and Bathyarchaeia taxonomy and phylogenomics

For their phylogenomic placement we used 36 Phylosift(*98*) markers (DNGNGWU00035: porphobilinogen deaminase, was omitted, since it yielded too few hits at our default 1E-5 e-value cutoff). We performed the homology searches, alignments, and dataset curation as described above. We added sequences of the Mnemosynella* species to a set of 183 taxa covering the taxonomic range of Archaea and *Ca*. H. orcuttiae* to the Bathyarchaeia representative genomes in GTDB r95. The 183 archaeal taxa included genomes from Hydrothermarchaeota and Geothermarchaeales binned here substituting their NCBI counterparts. We downloaded the representative genomes for Bathyarchaeia and Archaeoglobi from NCBI as nucleotide contigs (.fna files) and determined open reading frames for all genomes with Prokka(*97*) as above, omitting JdFR-11 and JdFR-21. As an outgroup for the Bathyarchaeia phylogeny, we used the Nitrososphaeria from the set of 183 archaeal

genomes, except for c__QMWL01 (Brockarchaeota*)(*99*) whose position was unstable in this case.

In both cases, we used IQ-Tree 2 to reconstruct the following phylogenies:

1) Model automatically selected by Modelfinder (-m MFP)

2) LG+C60+F+G (PMSF approximation with (1) as the guide tree)

3) Two phylogenies for each supermatrix under the GHOST heterotachy model(*100*). For the Bathyarchaeia dataset in the first phylogeny the number of categories was determined in Modelfinder (-mset LG -mrate E,H) and in the second phylogeny the maximum number of categories was set to three (-mset LG -mrate E,H -cmax 3). This corresponds to the highest number of categories for which the number of positions in the supermatrix approached or was greater than 10x the number of free parameters to be estimated in the model. For the complete archaeal dataset, Modelfinder crashed upon reaching H4, so the respective datasets were set to H3 and H2.

4) GTR4 with SR4-recoded(*101*) data (-mset GTR -mfreq F,FQ)

5) SR4C60 as in (*40*) (PMSF approximation with (4) as the guide tree)

6) GTR6 with Dayhoff6-recoded(*101*) data (-mset GTR -mfreq F,FQ)

7) A series of phylogenies with progressively desaturated subsets of the original supermatrix, under the model automatically selected by Modelfinder (-m MFP). The empirical Bayesian site-specific rates were calculated from the supermatrix and phylogeny in (2) with fixed branch lengths (-blfix) under the Poisson+G16 model. Here we performed a more extensive internal benchmark (Data S14). We estimated both empirical Bayesian ("random effects") and ML ("fixed effects") rates for the supermatrices under the Poisson, Poisson+G16, LG, LG+G16 models. Then we calculated Pearson and Spearman correlations between (i) rate estimation methods with a given model, (ii) substitution matrices, (iii) with and without rate heterogeneity. All correlations were strong, but the Poisson model was more internally consistent (Pearson r and Spearman rho >0.99 for Poisson against Poisson models; Pearson r >0.76, Spearman rho >0.97 for LG against LG models; Pearson r >0.69 and Spearman rho >0.93 for Poisson against LG models; all correlations were significant: Pearson and Spearman p<2.2E-16). G16 was chosen to imitate the behavior of Rate4Site(*102*) and because +R16 in IQ-Tree does not function together with -blfix.

8) LG+C60+F+G for the progressive desaturation datasets (PMSF approximation with the respective phylogenies from (7) as guide trees)

For all runs, branch supports were calculated with 1000 ultrafast bootstrap and 1000 aLRT SH-like replicates, and the approximate Bayes test.

To corroborate the taxonomic level and affiliation of the NRA7*/Mnemosynellales* (o__JdFR-21) and the Bathyarchaeota* Subgroups 20&22/Hecatellales* (o__B25) clades, we calculated pairwise ANI and AAI values for all GTDB r95 representative genomes in Archaeoglobi and Bathyarchaeia. JdFR-21 and JdFR-11 were substituted with the MAGs binned here. ANI values were calculated with orthoANI(*103*) and AAI with CompareM (https://github.com/dparks1134/CompareM). We repeated the same analyses for Hydrothermarchaeota and Geothermarchaeales, only with the genomes in GTDB r202 that had been released in the meantime.

To assess the biogeographic and environmental distribution of Mnemosynellales* and Hecatellales*, we constructed their 16S phylogenies. For Mnemosynellales* we used all sequences in SILVA classified under JdFR-20* (SILVA, SSU r138.1). We picked Hecatellales* sequences from among Bathyarchaeia sequences (SILVA Ref NR, SSU r138.1; SILVA contained >50k sequences), aligned with MUSCLE and through a preliminary BioNJ phylogeny(*85*). We used the

16S sequences from *Ca.* Polytropus marifundus* (GCA_002010305; s__JdFR-42 sp002010305) and RBG-16-48-13 (GCA_001775995; s__RBG-16-48-13 sp001775995) as outgroups for Mnemosynellales* and Hecatellales*, respectively. We aligned the final datasets with MAFFT L-INS-i v7.475(*104*), curated them manually, trimmed with BMGE (PAM100), and reconstructed an ML phylogeny with IQ-Tree 2 as above.

Statistical analyses

We performed all statistical tests in base R(*105*), except Dunn's test for which we used the dunn.test package(*106*). The functions are included in Data S16 (stats_analyses.txt). We visualized results using base R or ggplot2(*107*).

Supplementary Results & Discussion

Remarks on the reference phylogeny of Archaea

In most phylogenies while exploring the position of Hecatellales* (see Methods), we obtained a strongly supported monophyletic clade of Hydrothermarchaeota with Methanomada*. This is unlike GTDB that places Methanomada* with Thermococci in the phylum Methanobacteriota. Instead, we propose the name Phlegethonia* for this superclass or superphylum that includes multiple thermophiles, following the Underworld river naming trend of Stygia* (roughly corresponding to Hadarchaeota) and Acherontia* (Thermococci)(*7*). Based on their position, throughout the text, we considered Theionarchea* as members of Methanofastidiosales, together with Methanofastidiosaceae and f__NM3. The lineage Persephonarchaea* gets classified as Hadarchaeales when running GTDB-Tk(*108*) for GCA_003555245 but for convenience, due to their different metabolism and them not being integrated in GTDB, we treat them as separate here. Even though we assigned a circle to Nitrososphaerales due to the original classification of *Ca.* Methylarchaeum tengchongensis*(*8*), it is possibly a member of the Conexivisphaerales(*109*) that were not in our reference phylogeny dataset.

Functional annotation and evolution of the methanogenesis markers

m4-m9: A cluster of six genes that are co-localized in many genomes and are predicted to be co-transcribed in *Methanolobus psychrophilus* R15(*110*) but do not co-purify with Mcr(*111*). Some of them are absent from various alkane oxidizers but it is impossible to determine whether this is a result of genome incompleteness or variation in the processes controlled by these genes. There exist very few cases of putative remnants among them, for example in the hot spring Thaumarchaeota* SAG E04 (GCA_000405745) that possesses genes of the WLP(*112*). M9 is a homolog of McrC.
m10: AtwA (also called McrA2) is an ATP-binding protein(*113*) that is necessary for Mcr activation(*111*). We identify two homologs that we call here canonical AtwA and AtwA-like, assuming that the canonical AtwA has a wider taxonomic distribution. The presence of multiple homologs (AtwA1, AtwA2) can be found in the annotations of publicly available sequences but these do not correspond to the two clades we recovered i.e., there are both so-called AtwA1 and AtwA2 sequences within both canonical AtwA and AtwA-like. AtwA-like is absent in most Halobacteriota methane metabolizers. This distribution does not seem to be linked to the presence of multiple Mcr homologs in Methanomada* members, as the duplication of the *atwA* gene most probably occurred at the LMA, while the aforementioned Mcr complexes arose from more recent duplications (Fig. 2A). The topology of AtwA-like with Methanosarcinia and Methanosarcinia_A at the base and non-monophyletic Methanomada* might have been affected by long branch attraction. However, the canonical AtwA side recovers the monophyly of most lineages as expected from McrABG (Fig. 2A) and corroborates the poorly supported NONREV root at Halobacteriota. This in turn supports that anaerobic alkane oxidation (AAO) is a more recent invention but Acr complexes still require AtwA.

m11: McrC probably participates in Mcr activation, since it is part of a reductase complex capable of reducing coenzyme F_{430} to the Ni^{1+} form(*111*) and corresponds to part of the McrA3 component(*113*). Other than McrC and m9, we identify a third distant homolog only present in Methanobacteria (Data S2; Fig. S26 only shows the canonical McrC).
m12-m14: McrD (m12) has been posited to be a chaperone delivering F_{430} to apo-Mcr after the final step of its biosynthesis, catalyzed by CfbE(*114*). However, we think that this association might not be universal, since McrD is present in essentially all methane metabolizers and alkane oxidizers, except for Methanophagales. CfbD (m13) and CfbE (m14) have a different distribution, with one or both being absent in many AAO and even some methanogens. CfbE is encoded by one o__ANME-1 MAG (GCA_004212135, hot spring). These discrepancies indicate that several Archaea might contain F_{430} biosynthesis variants or even a modified cofactor, such as the methylthio modified F_{430} found in the Mcr in marine o__ANME-1(*115*, *116*).
m15: MtxX is either a phosphotransacetylase or methyltransferase, part of the MtxXAH operon in *M. barkeri*(*117*, *118*). It is impossible to determine its function from its distribution and phylogeny, since it does not consistently coexist with some other methanogenesis or WLP subsystem but the association with Mtr is probably a rarity.
m16: M16 has sequence similarity to the AIR synthase but its function is unknown. The *M. maripaludis* homolog (MMP0291) is annotated as "hydrogenase expression/formation protein related" and in proximity to some *hyp* genes(*31*).
m17: DUF1743 includes a series of homologs that contain one of the domains of TiaS, the $tRNA^{Ile}$-agmatinylcytidine synthetase (that homolog was removed from the final phylogeny shown in Fig. S33) and is very ancient among Archaea, independent of methane metabolism. It is predicted to function as an ATPase(*119*) but its exact function in methane metabolism or as a remnant remains indeterminate.
m18-m19: No information in the literature, function cannot be predicted.
m20: M20 contains homologs of YcaO that functions as a glycine thioamidase for the post-translation modification of an Mcr glycine (Gly465 in *M. acetivorans*) into thioglycine(*120*). The topology of our phylogeny is roughly in agreement with the one published in (*120*). This modification is absent in alkane oxidizers.
m21: Rossmann fold protein with a cysteinesulfonic acid modification (PDB ID: 2R47). No further information can be determined.
m22: No useful information.
m23: Annotated as P-type ATPase or HAD hydrolase. Absent in Methanopyri.
m24: No useful information.
m25: Sparse distribution: absent in Methanopyri and many Halobacteriota lineages but widely present in c__CR-4. Related to dihydrouridine synthase (PF0127). It is co-localized with formylmethanofuran--tetrahydromethanopterin formyltransferase/F_{420}-dependent NADP oxidoreductase in c__CR-4 and $F_{420}H_2$ oxidase in Mnemosynella*, thus a role in energy/hydrogenase-type reactions involving F_{420} is possible.
m26: Based on its distribution, phylogeny, and synteny, m26 is associated with Mtr, even including the Thorarchaeia clade of the canonical MtrA. Its distribution also includes Persephonarchaea sequences (Data S2) at twilight zone homology that aligned poorly and ended up on a long branch, thus we removed them. The presence of a divergent m26 in Persephonarchaea* suggests that they are former methane metabolizers (CO_2-reducing hydrogenotrophic or AMO) that have already lost all traces of Mtr and are currently also losing its related marker.
m32: UPF0285 is not found outside of Euryarchaeota*. Its function cannot be deduced. We recover a strong monophyly of Hydrothermarchaeota (where it is syntenic with Ftr) with Methanomada*. This agreement with the species tree (Fig. 1B) and synteny supports the case for Hydrothermarchaeota also being former CO_2-reducing hydrogenotrophic methanogens. Absent in

Methanopyri; the *M. kandleri* gene annotated as UPF0285 (MK0078) is a member of the unrelated DUF1464.
m33: MamA is a B_{12}-dependent radical SAM methyltransferase that catalyzes the methylation of Arg285 (*M. acetivorans*)(*121–123*). Its distribution is limited to Methanomada* and a subset of Halobacteriota. Together with the distribution and phylogeny of McmA(*124*) and our phylogeny of m20, it suggests that the *M. acetivorans* pattern of Mcr posttranslational modifications is not universal among methane metabolizers and entirely absent in alkane oxidizers without hampering the complex's functionality. There exists evidence for this from the structure of Mcr from *Methermicoccus shengliensis* (Methanosarcinia_A)(*125*).
m34: Limited to Methanomada* and a subset of Halobacteriota. Its function cannot be deduced.
m35: Limited to Methanomada* and a subset of Halobacteriota. Its function cannot be deduced. There exists one solved structure (PDB ID: 1KJN) of the *M. thermautotrophicus* homolog that is a selenomethionine-containing homodimer.
m36: Sparse distribution in Methanomada* and a subset of Halobacteriota. The phylogeny is very poorly resolved.
m37: Its distribution includes Persephonarchaea* where it is syntenic with Fwd and Ftr. The synteny could be related to Persephonarchaea* being former methane metabolizers (see below about Eha). We cannot infer anything about its function.
m38: Limited to Methanomada*, Methanosarcinia, and Methanomicrobia. Its function cannot be deduced.
Based on their distributions and phylogenies, some of the 38 methanogenesis markers should probably not be considered as such, instead being "demoted" to partial markers like the various Eha and Ehb subunits.
A few markers (m15, m17, m18, m19, m21, m24, m36) have undergone interdomain horizontal transfers from Archaea to Bacteria, suggesting that they might be incorporated into other metabolic pathways without them necessarily being remnants of methane metabolism.
M15, m16, and m17 each contain a strongly monophyletic Archaeoglobaceae cluster (including *Ca.* Methanomixophus hydrogenotrophicum*) with *Ca.* Methanodesulfokores washburnensis (GCA_004347975, Korarchaeia) inside it. This transfer direction is opposite to what we observe for Mcr but is consistent with our inference that other methyl-dependent hydrogenotrophic methanogenesis components have been acquired horizontally in Methanodesulfokores.

Evolution of the Hcg proteins

We observe that in Methanococci the genomic location of the *hcg* genes is not stable. *hcg*BC are clustered together in *M. jannaschii* and distant from *hmd* but *hcgAG* and *hmd* are syntenic (Fig. S19), corroborating a previous synteny analysis in *M. maripaludis*(*126*). This is reflected in the evolutionary histories of the single gene phylogenies and concatenations where HcgBC and HcgAEFG give different positions for Methanococci that are further supported by the gene and site concordance factors for the two concatenations (Data S10). For HcgBC, there exists a monophyletic clade of Methanobacteria and Methanococci similar to Hmd and Hmd-like. However, in HcgAEFG, Methanobacteria are monophyletic with Methanopyri, similar to the archaeal species tree (Fig. 1B), albeit not strongly supported. Other than a lack of evolutionary pressure to maintain a tight cluster, these data imply the involvement of homologous recombination events in the evolution of the Hcg pathway and Hmd, barring other sources of bias in the phylogenies. Assuming Hmd and HcgBC represent the correct history, then the most parsimonious scenario involves an ancient lateral acquisition of HcgAEFG by Methanobacteria or by Methanococci. The latter case assumes a subsequent dissolution of the cluster in Methanococci and the lack of resolution in the HcgAEFG phylogeny not conflicting with the Methanopyri-Desulfurobacteriaceae monophyly in the HcgBC tree. Nevertheless, if we accept that the branching in Methanomada* should reflect the archaeal reference tree, it is HcgBC-Hmd that were transferred horizontally to the ancestral Methanobacteria

or Methanococci. In any case, an acquisition by the Desulfurobacteriaceae in a hydrothermal environment is a plausible assumption. Note that in all cases the Methanobacteria clade includes a recent transfer to Methanomicrobia as previously noted for Hmd/Hmd-like(*26*).

For the HcgBC concatenation, both MAD and MinVar root the tree at the strongly monophyletic group of Methanopyri-Desulfurobacteriaceae (with relatively high rootstrap support) while NONREV roots it at the strongly monophyletic group of Methanobacteria (with low rootstrap support). Neither root agrees with what we would expect from the reference tree i.e., a basal separation of Methanococci (Fig. S19B, as displayed). Regarding the HcgAEFG concatenation, MAD and Minvar disagree in the root placement. MAD roots at Methanopyri and MinVar at Desulfurobacteriaceae. The rootstrap values strongly support the latter but both are roughly consistent with what we have observed previously for Hmd/Hmd-like(*26*). In any case, this biosynthetic pathway does not appear to predate the ancestor of Methanomada*. Finally, as mentioned in the Supplementary Methods, the Desulfurobacteriaceae do not seem to have a proper HcgD but we did not even recover a proper HcgD monophyletic clade in the preliminary phylogenies either. Thus, it is possible that various paralogs (perhaps with some promiscuity) catalyze the HcgD reaction in different taxa.

<u>Evolution of Eha</u>

Our main interest in the evolution of Eha was the direction of the transfer between Mnemosynellales* and Persephonarchaea*. With a root between Methanomada* and Halobacteriota (Fig. 3A, as displayed), the Persephonarchaea* are not in their expected position (Fig. 1B) and have acquired Eha laterally. This scenario is consistent with the high number of methanogenesis markers in Mnemosynellales* and the phylogenies of WLP genes from both the carbonyl and methyl branches (CdhB, Mch, Mtd; Figs. S10-12, Data S3). MAD and MinVar outgroup-free rooting placed the root between Methanomicrobia and the remaining clades reversing the inferred direction of the transfer (Fig. 3A, Data S9). We recover this scenario in the phylogenies of the WLP carbonyl methyltransferase module genes (CdhDE) (Figs. S8, S9, Data S3); Eha could have been part of the same transfer event. This inference also suggests that Persephonarchaea* could be former methanogens, given that they possess some markers but their position in these phylogenies is unresolved.

A second issue is the second (smaller) Methanobacteria clade. It contains genomes from a variety of genera and often genomes of partially undefined taxonomy (Data S7). There is no overlap of taxa between the two Methanobacteria clades, indicating that there were no gene duplications involved but rather ancient homologous recombination events. As with Hcg, we cannot define the original Methanobacteria clade and by extension a putative source of the transfer although following the species tree (Fig. 1B), the smaller Methanopyri-affiliated clade is the original. Determining the position of the Methanopyri themselves in reference phylogenies has been long plagued by artifacts(*127*, *128*) and it is likely that such issues carry over to the Hcg and Eha phylogenies. The issue is exemplified by the disagreements in the phylogenetic signal among subunits by proxy of their sCF (Fig. S15A, Data S10) where Node 232 (Methanopyri + small Methanobacteria) and Node 153 (Methanococci + large Methanobacteria) have the lowest values among deeper nodes in the tree. Other low sCF values for Node 151 (Mnemosynellales* + Persephonarchaea*) could be related to divergence in their sequences and poor signal. Within Methanobacteria, we observed the absence of Eha in *Methanosphaera*, despite it possessing the WLP(*26*) and Ehb (this study). Since *Methanosphaera* is a methyl-dependent hydrogenotrophic methanogen, it would appear that at least for Methanobacteria there exists a tight association between CO_2-reducing hydrogenotrophic methanogenesis and Eha that has not persisted after the change in substrate. Judging from its absence in other methanogens (e.g., Nezhaarchaeales, Methanomethylicales), Eha is not always indispensable for CO_2-reducing hydrogenotrophic methanogenesis. Depending on whether the Euryarchaeota* are monophyletic or not(*34*) and the

direction of the Mnemosynellales* - Persephonarchaea* transfer, the number of inferred losses of Eha among other Archaea varies widely.
Although the identity and homology of ferredoxin homologs is fraught with ambiguity, there seem to have been many modifications of the complex arrangement in Methanomada* involving the EhaPQR subunits that are MvhB-like ferredoxins. The Eha operon in many Methanobacteria includes an extra gene compared to what was characterized in *Methanothermobacter thermautotrophicus* in (*129*). Beyond EhaQRST homologs in Methanomada* clades, additional modifications can be traced to the origin of specific clades through subunit gains and losses. EhaP, while almost certainly present at the LMA, has been lost by Persephonarchaea*. EhaI was similarly lost at the origin of Methanomicrobia. EhaL is absent in Methanomicrobia, Persephonarchaea*, and Mnemosynellales*. Together with the uncertainty of the Eha phylogeny root, it is impossible to determine whether EhaL was lost by these lineages, or invented at the origin of Methanomada*, and by extension how ancient it is. Even more complicated is the case of EhaA that is missing from Methanomicrobia and the smaller Methanobacteria clade. The simplest scenario posits two individual loss events but the poorly resolved EhaA phylogeny (Data S7) is also compatible with a more complex history: EhaA emerged at the base of Methanomada* (or lost by Methanomicrobia, Persephonarchaea*, and Mnemosynellales* in one or more events) and then transferred twice separately to Persephonarchaea* and Mnemosynellales*. The patchy distribution of EhaAILP could mean that they are non-essential for the function of the hydrogenase. Alternatively, the missing subunits in Methanomicrobia, Persephonarchaea*, and Mnemosynellales* might be related to lack of coupling between CO_2-reducing hydrogenotrophic methanogenesis (with the exception of some Methanomicrobia) and Fwd through Eha.

<u>Evolution of Ehb</u>

As mentioned in the Results & Discussion section, the most extraordinary event in the evolution of Ehb is the homologous recombination in the Methanococci. Even though we suspected its existence from the synteny and single-gene phylogenies (Data S8), only rarely was the position of Methanococci strongly supported. To investigate the signal difference among subunits and its origin, we reconstructed phylogenies for three different Ehb concatenations: one with all subunits (Fig. 3B), one corresponding to the Methanococci syntenic cluster (EhbEFGHIKLMO, EhbJ was omitted due to its small size and inconsistent topology; Fig. S13), and one for the Methanococci cluster without subunits that recover the monophyly of Methanomada* (EhbEGHIKLM; Fig. S14). The Methanococci cluster originated from a massive homologous recombination event related to the Methanofastidiosales, perhaps followed by further transfers or fast evolution of certain subunits that ended up determining the phylogenetic signal of the concatenations.
We further tested the putative homologous recombination of Ehb in Methanococci through our variant application of sCF (see Methods). In the sCF heatmap for the concatenation of all Ehb subunits (Fig. S15C), the nodes with low sCF values in multiple subunits are 208 and 113. Node 208 simply reflects the uncertainty in the internal branching of Methanofastidiosales, although transfer events cannot be discounted. Node 113 corresponds to Methanomada*; here it is subunits EhbBEGH that have lower values. With the exception of EhbB, all of them are part of the Methanococci Ehb cluster. Most subunits of the cluster are also found together in the Euclidean clustering of the heatmap. This situation persists for the EhbEFGHIKLMO concatenation (Fig. S14C) and only partially resolves for EhbEGHIKLM (Fig. S14D) where Methanococci group with Methanofastidiosales, as for some subunits (especially EhbEG) their position remains inconsistent due to pervasive differences in the signal or further recombination, as described above. We observed the same behavior for gCF. For the complete Ehb concatenation the gCF for Methanofastidiosales is 81.25 and for Methanomada* 31.25 demonstrating the signal clash among subunits. For EhbEFGHIKLMO they drop to 77.78 and 22.22 respectively. Most of the signal for

a monophyletic Methanomada* has almost vanished apart from EhbFMO. Finally, in EhbEGHIKLM Methanococci and Methanofastidiosales are monophyletic at 71.43 gCF.

The reasons for Ehb being retained by methyl-dependent hydrogenotrophic methanogens in the absence of the WLP is not entirely clear. In the case of Methanofastidiosaceae and f__NM3, it seems like a last-ditch solution, since they lack any other hydrogenases(*11*). The Methanofastidiosales ancestor had already lost Eha despite our inference that it was an CO_2-reducing hydrogenotrophic methanogen, given the presence of the WLP in Theionarchaea*(*26*). In the case of Methanomethylicales, Ehb has persisted in both methyl-dependent hydrogenotrophic and CO_2-reducing hydrogenotrophic methanogens even though Eha had already been lost and is not found in other Thermoproteota methanogens either.

<u>Notes on the evolution of the WLP</u>

Owing to the higher number of available genomes since two recent in-depth analyses of the WLP(*26*, *36*), we made two noteworthy observations. In the carbonyl branch article(*36*), an additional set of carbonyl branch enzymes in Syntropharchaeales was ignored, since they had been deemed to be too divergent and forming long branches in preliminary phylogenies. In this study, we identified a close relative of that set in a basal alkanotrophic o__ANME-1(*130*). The sequences from Syntropharchaeales and o__ANME-1 form a monophyletic clade but their branching position is not consistent among subunits. In contrast, the main Syntropharchaeales carbonyl branch sequences are found consistently within Halobacteriota (Figs. S8-10). We hypothesize a participation of this carbonyl branch version in AAO but its exact function cannot be determined. The evolution and comparative genomics of the carbonyl branch will eventually need to be reexamined as more genomes become available. Concerning the H_4MPT branch, in the Mtd tree we find three Proteobacteria* sequences forming a monophyletic branch inside Thorarchaeia, a clear interdomain transfer and the first such case for Mtd (Fig. S12). As the clade contains multiple (and non-identical) sequences, we could discount the possibility of binning errors. These Proteobacteria* MAGs need to be studied in depth to determine the role of Mtd. Our preliminary hypothesis is that they are part of either a hybrid H_4MPT branch with Mtd instead of bMtd in methylotrophy, or that Mtd is substituting one of the functions of FolD in the H_4F branch.

<u>Descriptions of proposed taxa</u>

The type species for each genus or higher rank taxon are denoted in brackets. Proposed names are given next to the type MAG in Table S2. The ecology/habitats of the species are presented collectively below. Metabolic predictions are found below and in the main text. We have omitted the asterisks of non-GTDB taxa added elsewhere from these descriptions.

<u>Description of *Candidatus* Mnemosynella (gen. nov.)</u>

Mne.mo.sy.nel'la. N.L. fem. dim. n. *Mnemosynella*, little Mnemosyne, after the Greek mythology Titaness, goddess of memory, and mother of the Muses; a reference to the species in the genus containing multiple methanogenesis markers and "remembering" their methanogenic ancestry. Equivalent to GTDB g__JdFR-21.

<u>Description of *Candidatus* Mnemosynella biddleae (sp. nov., type)</u>

bidd'le.ae. N.L. gen. fem. n. *biddleae*, named after Jennifer Biddle, in honor of her contributions to microbial ecology. Equivalent to GTDB s__JdFR-21 sp002011165.

<u>Description of *Candidatus* Mnemosynella bozhongmuii (sp. nov.)</u>

bo.zhong.mu'i.i. N.L. gen. masc. n. bozhongmuii, named after Bo-Zhong Mu, in honor of his contributions to microbial ecology, including obtaining the original samples from the Shengli oil field used in this study. Equivalent to GTDB s__JdFR-21 sp014361165.

<u>Description of Mnemosynellaceae (fam. nov.)</u>

Mne.mo.sy.nel.la.ce’ae. N.L. fem. n. *Mnemosynella*, a Candidatus genus name; -*aceae*, ending to denote a family; N.L. fem. pl. n. *Mnemosynellaceae*, the *Mnemosynella* family. Equivalent to GTDB f__JdFR-21.

Description of Mnemosynellales (ord. nov.)

Mne.mo.sy.nel.la’les. N.L. fem. n. *Mnemosynella*, a Candidatus genus name; -*ales*, ending to denote a family; N.L. fem. pl. n. *Mnemosynellales*, the *Mnemosynella* order. Equivalent to GTDB o__JdFR-21.

Description of *Candidatus* Hecatella (gen. nov)

He.ca.tel’la. N.L. fem. dim. n. *Hecatella*, little Hecate, after the Greek mythology goddess of witchcraft and crossroads; a reference to their metabolism being at the “crossroads” of methanogenesis and the Wood-Ljungdahl pathway by means of its Mtr complex. Equivalent to GTDB g__JdFR-11.

Description of *Candidatus* Hecatella orcuttiae (sp. nov., type)

or.cut’ti.ae. N.L. gen. fem. n. *orcuttiae*, named after Beth Orcutt, in honor of her contributions to microbial ecology, including studies on the Juan de Fuca Ridge. Equivalent to GTDB s__JdFR-11 sp002011035.

Description of Hecatellaceae (fam. nov.)

He.ca.tel.la.ce’ae. N.L. fem. n. *Hecatella*, a Candidatus genus name; -*aceae*, ending to denote a family; N.L. fem. pl. n. *Hecatellaceae*, the *Hecatella* family. Equivalent to GTDB f__B25.

Description of Hecatellales (ord. nov.)

He.ca.tel.la’les. N.L. fem. n. *Hecatella*, a Candidatus genus name; -*ales*, ending to denote a family; N.L. fem. pl. n. *Hecatellales*, the *Hecatella* order. Equivalent to GTDB o__B25.

Description of *Candidatus* Geothermarchaeum (gen. nov)

Ge.o.therm.ar.chae’um. Gr. fem. n. *gê*, the earth; Gr. masc. adj. *thermos*, hot; N.L. neut. n. *archaeum*, ancient one, archaeon, from Gr. masc. adj. *archaîos*, ancient; N.L. neut. n. *Geothermarchaeum*, an archaeon from hot earth. Equivalent to GTDB g__JdFR-14.

Description of *Candidatus* Geothermarchaeum rappei (sp. nov., type)

rap.pe’i. N.L. gen. masc. n. *rappei*, named after Michael Rappé, in honor of his contributions to microbial ecology, including obtaining the original samples from the Juan de Fuca Ridge used in this study. Equivalent to GTDB s__JdFR-14 sp002011085.

Description of *Candidatus* Scotarchaeum (gen. nov.)

Scot.ar.chae’um. Gr. masc. n. *skotos*, darkness; *archaeum*, ancient one, archaeon, from Gr. masc. adj. *archaîos*, ancient; N.L. neut. n. *Scotarchaeum*, an archaeon living in darkness. Equivalent to GTDB g__JdFR-13.

Description of *Candidatus* Scotarchaeum ottlingeri (sp. nov., type)

ott.lin’ge.ri. N.L. gen. masc. n. *ottlingeri*, named after Markus Ottlinger, German visual artist depicting hydrothermal settings and biofilms. Equivalent to GTDB s__JdFR-13 sp002011075.

Description of Geothermarchaeaceae (fam. nov.)

Ge.o.therm.ar.chae.a.ce’ae. N.L. neut. n. *Geothermarchaeum*, a Candidatus genus name; -*aceae*, ending to denote a family; N.L. fem. pl. n. *Geothermarchaeaceae*, the *Geothermarchaeum* family. Equivalent to GTDB f__JdFR-13.

Description of Geothermarchaeales (ord. nov.)

Ge.o.therm.ar.chae.a’les. N.L. fem. n. *Geothermarchaeum*, a Candidatus genus name; -*ales*, ending to denote a family; N.L. fem. pl. n. *Geothermarchaeales*, the *Geothermarchaeum* order. Equivalent to GTDB o__Geothermarchaeales.

Description of *Candidatus* Pyrohabitans (gen. nov.)

Py.ro.ha’bi.tans. Gr. neut. n. *pŷr*, fire; L. pres. part. *habitans*, inhabiting; N.L. masc. n. *Pyrohabitans*, an inhabitant of fire. Equivalent to GTDB g__JAADFX01.

Description of *Candidatus* Pyrohabitans jungbluthii (sp. nov., type)

jung.blu'thi.i. N.L. gen. masc. n. *jungbluthii*, named after Sean Jungbluth, in honor of his contributions to microbial ecology, including obtaining the original samples from the Juan de Fuca Ridge used in this study. Not present in GTDB (failed quality check).

Environment

1) *Ca.* Mnemosynella biddleae*, *Ca.* Hecatella orcuttiae*, *Ca.* Geothermarchaeum rappei*, *Ca.* Scotarchaeum ottlingeri*, *Ca.* Hydrothermarchaeum profundi*, *Ca.* Pyrohabitans jungbluthii*: Basalt-hosted (3.5 million year-old oceanic crust) anoxic crustal fluids, 65 °C, on the Juan de Fuca Ridge flank (Northeast Pacific Ocean)(*131*).

2) *Ca.* Mnemosynella bozhongmuii*: Production water from the Shengli oil field (Block Z3, 52 °C), China(*47*).

Additional metabolic predictions

Ca.* Scotarchaeum ottlingeri: *Ca.* S. ottlingeri* (Fig. S50A) possesses a complete WLP that could run reductively or oxidatively. The pathway can additionally be entered at methylene-H_4MPT, either through the condensation of formaldehyde by Fae/Hps, or through the Glycine Cleavage System (GCS). It codes for a modified TCA cycle that lacks pyruvate carboxylase but instead deploys a citrate synthase to produce citrate from acetyl-CoA and oxaloacetate. It has a cytoplasmic HdrABC/MvhADG whose substrate is regenerated by a syntenic membrane-bound HdrD/GcvH (the heterodisulfidic bond forming/breaking component of the GCS), in lieu of the HdrDE found in many methanogens with cytochromes. The main hydrogenase is a hybrid Fpo/Mch-like, with the Fpo-like component having sequence similarity to the Hfo hydrogenase described in Bathyarchaeia(*37*, *132*). The quinones shuttled by Fpo-like originate from either HdrD/GcvH or Sdh in the TCA cycle.

Ca.* Geothermarchaeum rappei: *Ca.* G. rappei* (Fig. S50B) encodes a TCA cycle that is very similar to *Ca.* S. ottlingeri, except the citrate to isocitrate isomerization for which it probably uses a putative AcnX-like (aconitase superfamily)(*133*). Strangely, it possesses both serine hydroxymethyltransferase and the GCS but no WLP, thus methylene-H_4MPT/H_4F is only exchanged with pyrimidine biosynthesis. Its main putative electron acceptor during heteotrophic growth could be Fe(III), *Ferroglobus placidus*(*134*). This is supported by the presence of genes coding for both ferric (Afu) and ferrous (Feo) iron transport(*135*). A membrane bound Vht hydrogenase could exchange quinones with an Fpo/Mch-like complex, much like that of Ca. S. ottlingeri. However, whether Vht produces hydrogen or Fe(II) or even operates in the opposite direction cannot be determined.

Ca.* Pyrohabitans jungbluthii: *Ca.* P. jungbluthii's* (Fig. S50C) metabolism revolves around the WLP, entered oxidatively from acetate, formaldehyde, or methylated compounds. While its main electron sink seems to be thiosulfate reduction, it also codes for a periplasmic nitrate reductase (Nap), with similarity to that described in *Thermosulfurimonas dismutans*(*136*). It is possible that electrons for this reaction are derived from reduced quinones produced by an Fpo/Ech hybrid hydrogenase. Furthermore, a [NiFe] group 2f hydrogenase(*137*) related to the Huc hydrogenases of group 2a, produces electrons from hydrogen but their acceptor cannot be determined.

Ca.* Hydrothermarchaeum profundi: The metabolism of *Ca.* H. profundi* (Fig. S50D) has been previously described in (*138*, *139*). It can enter the WLP oxidatively from acetate, methylamines, or formaldehyde, and is a sulfate reducer. A hybrid membrane protein comprising of an Fdo part and the small subunit of a Hys hydrogenase recycles formate producing electrons that get utilised by an Nxr oxidoreductase, highly similar to the one described in *Kuenenia stuttgartiensis*(*140*). Together with Nxr, an Fpo/Mch-like hydrogenase recycles menaquinones at the membrane.

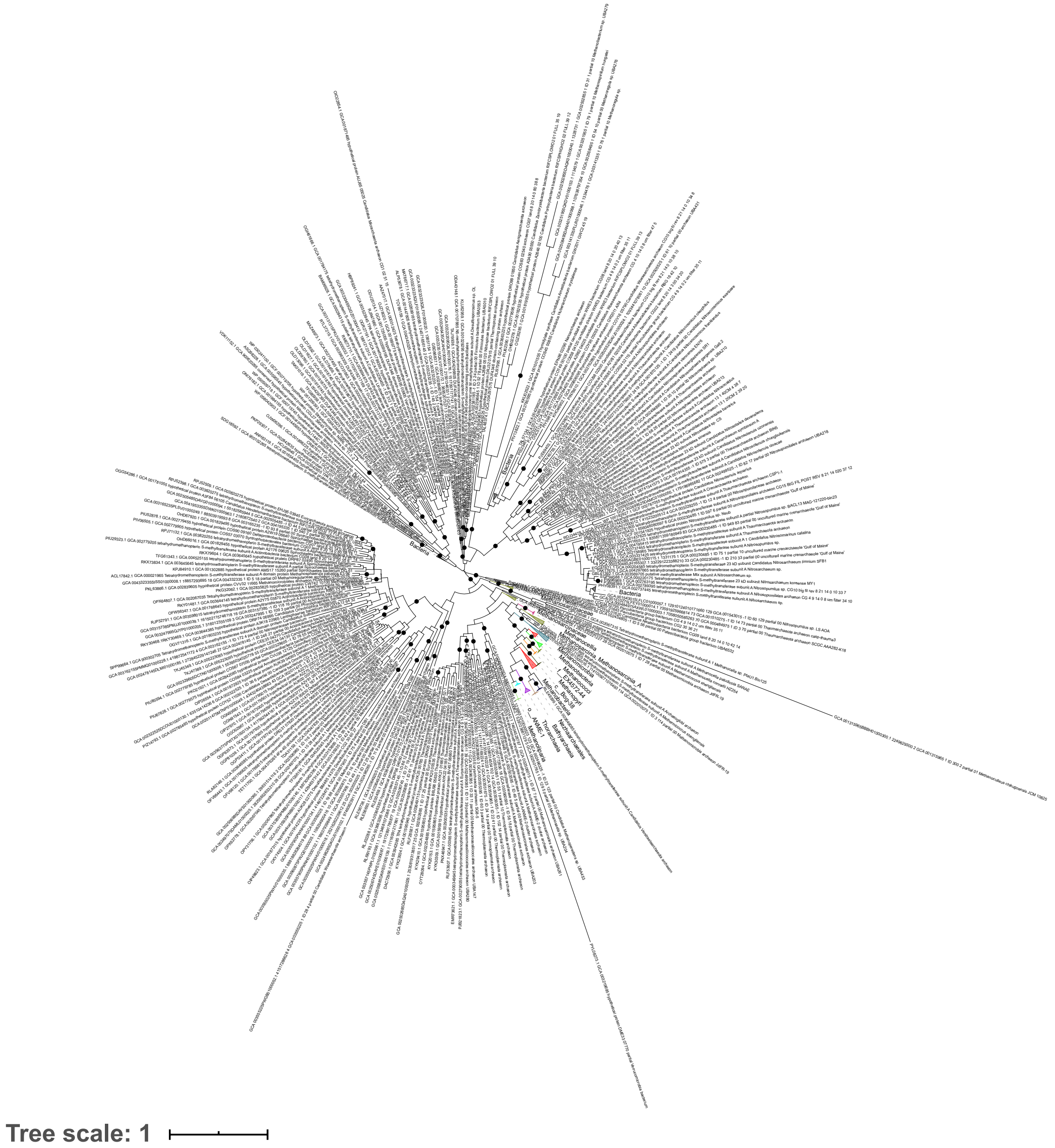

Fig. S1.
ML phylogeny of MtrA. Black circles indicate strongly supported branches (ultrafast bootstrap >=95, aLRT SH-like >=80).

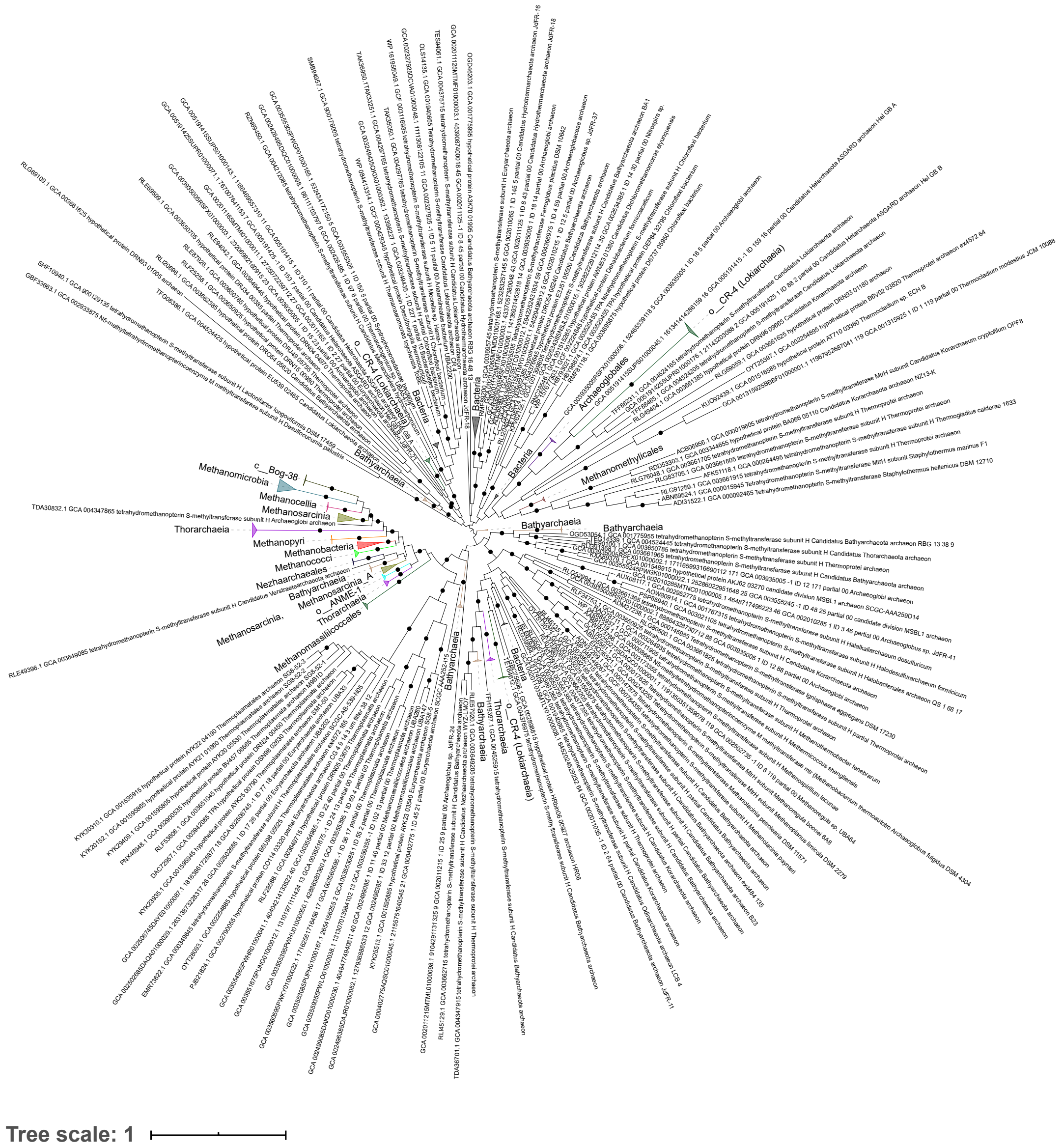

Fig. S2.
ML phylogeny of MtrH. Black circles indicate strongly supported branches (ultrafast bootstrap >=95, aLRT SH-like >=80).

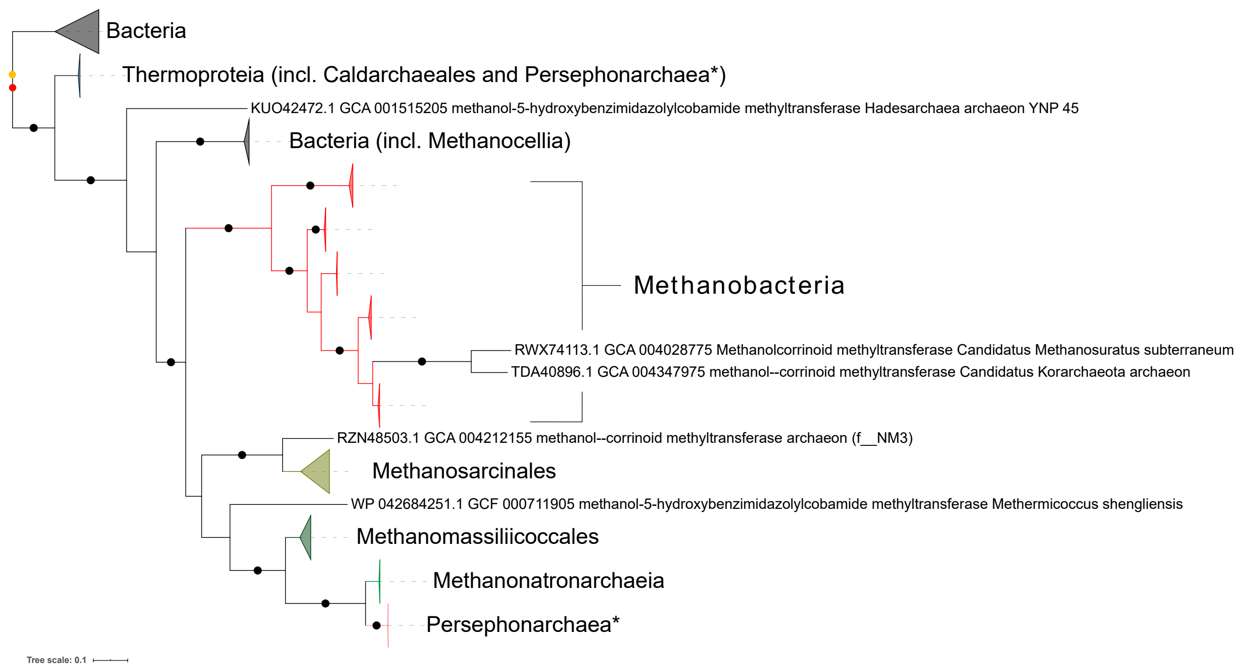

Fig. S3.

ML phylogeny of MtaB. Black circles indicate strongly supported branches (ultrafast bootstrap >=95, aLRT SH-like >=80), red circle corresponds to the MAD root, orange to MinVar.

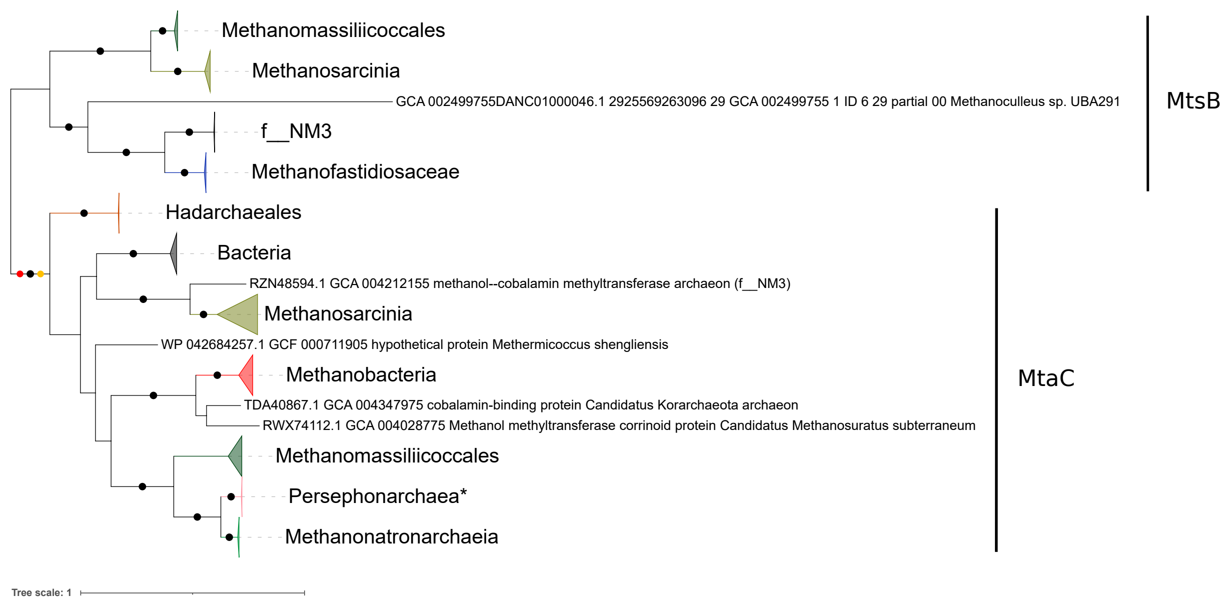

Fig. S4.
ML phylogeny of MtsB. Black circles indicate strongly supported branches (ultrafast bootstrap >=95, aLRT SH-like >=80), red circle corresponds to the MAD root, orange to MinVar.

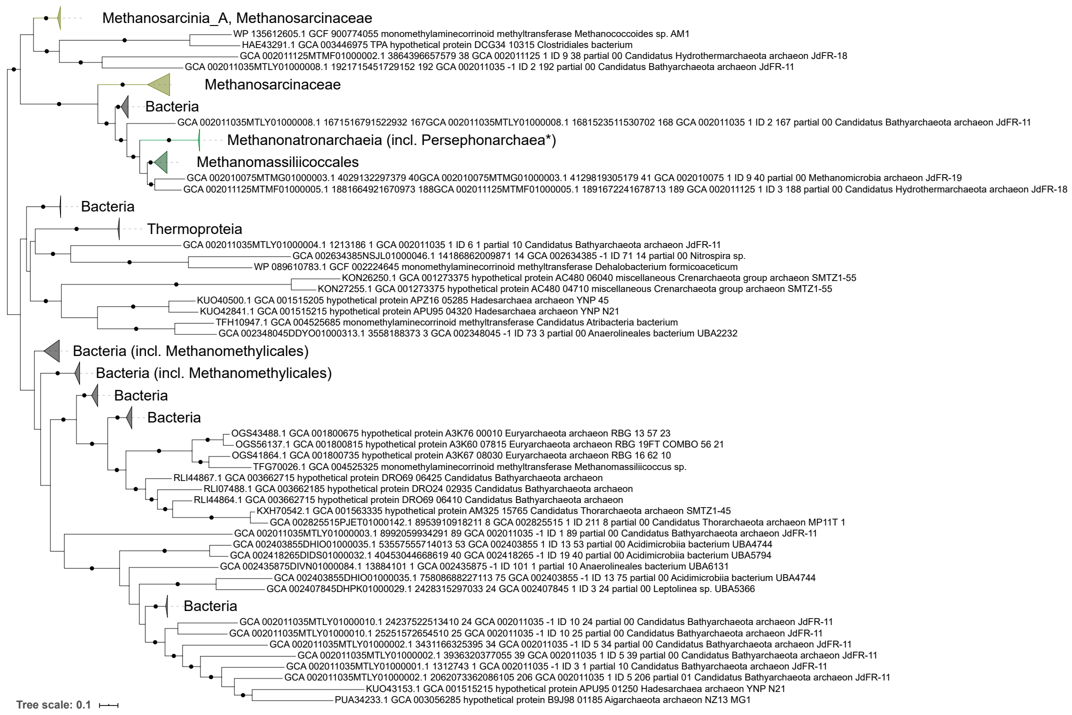

Fig. S5.

ML phylogeny of MtmB. Black circles indicate strongly supported branches (ultrafast bootstrap >=95, aLRT SH-like >=80).

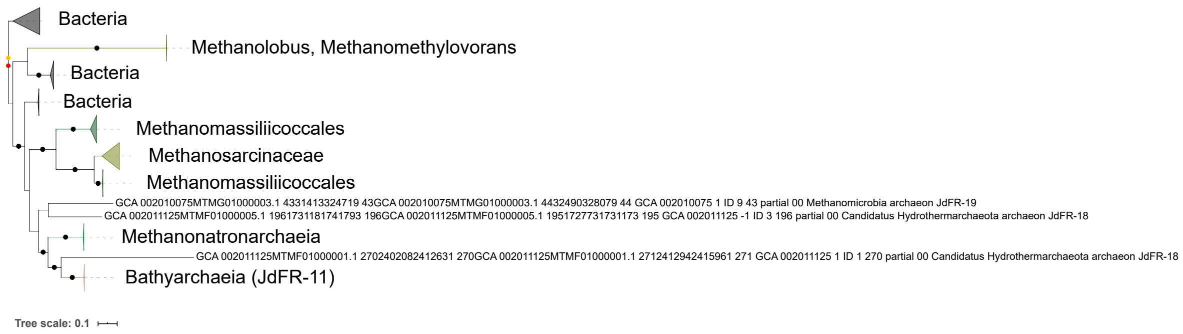

Fig. S6.
ML phylogeny of MtbB. Black circles indicate strongly supported branches (ultrafast bootstrap >=95, aLRT SH-like >=80), red circle corresponds to the MAD root, orange to MinVar.

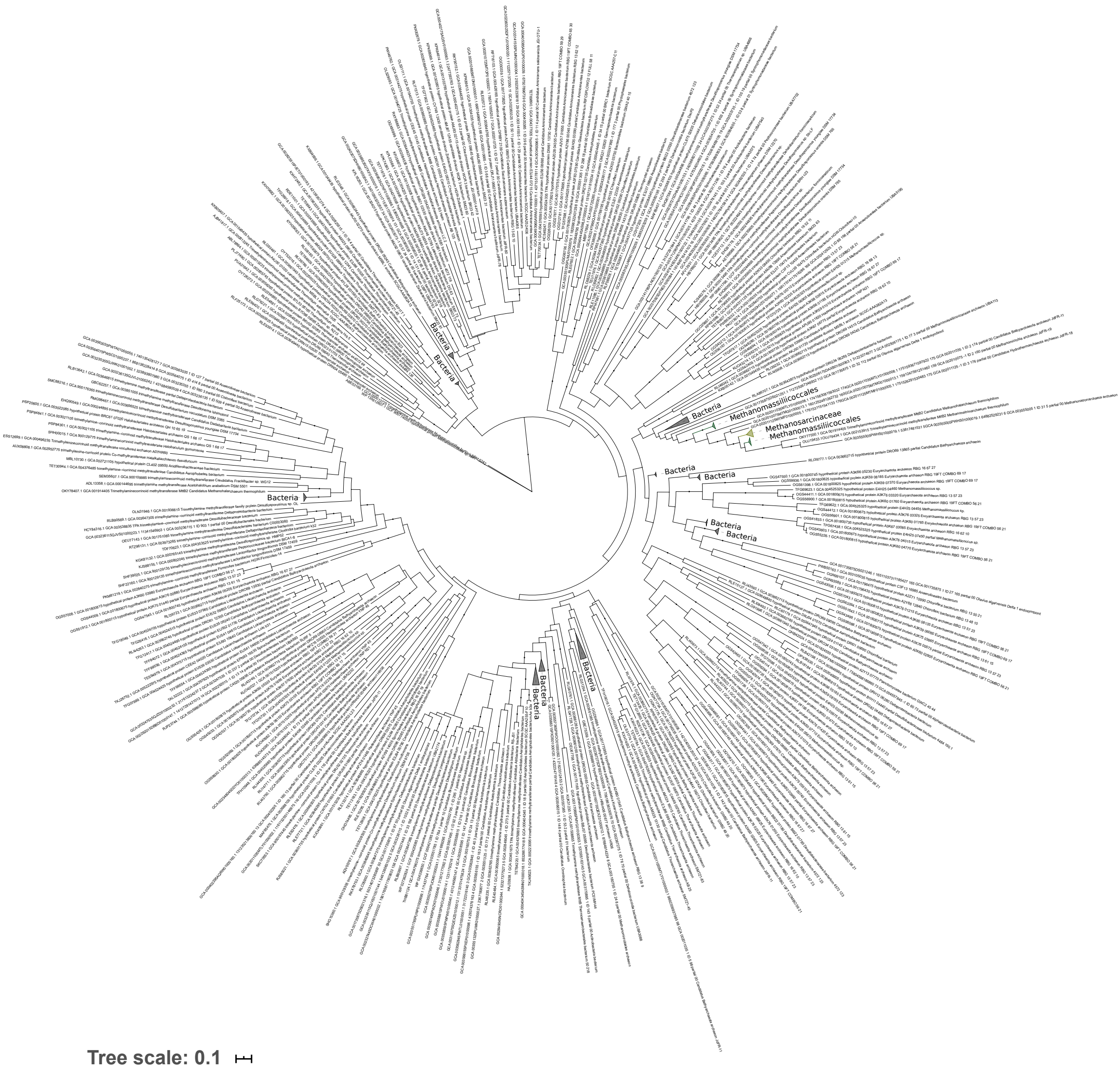

Fig. S7.
ML phylogeny of MttB. Black circles indicate strongly supported branches (ultrafast bootstrap >=95, aLRT SH-like >=80).

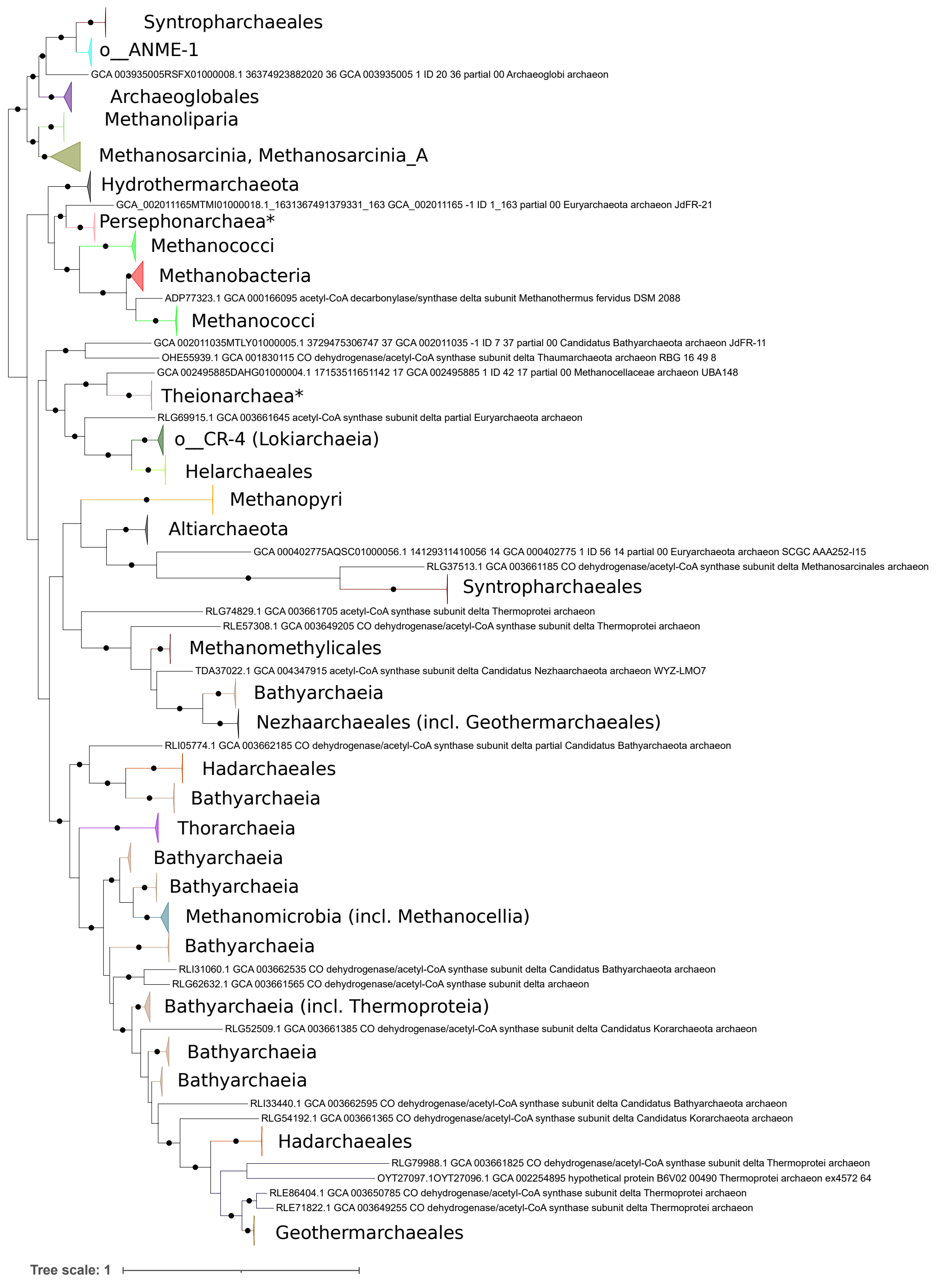

Fig. S8.
ML phylogeny of CdhD. Black circles indicate strongly supported branches (ultrafast bootstrap >=95, aLRT SH-like >=80).

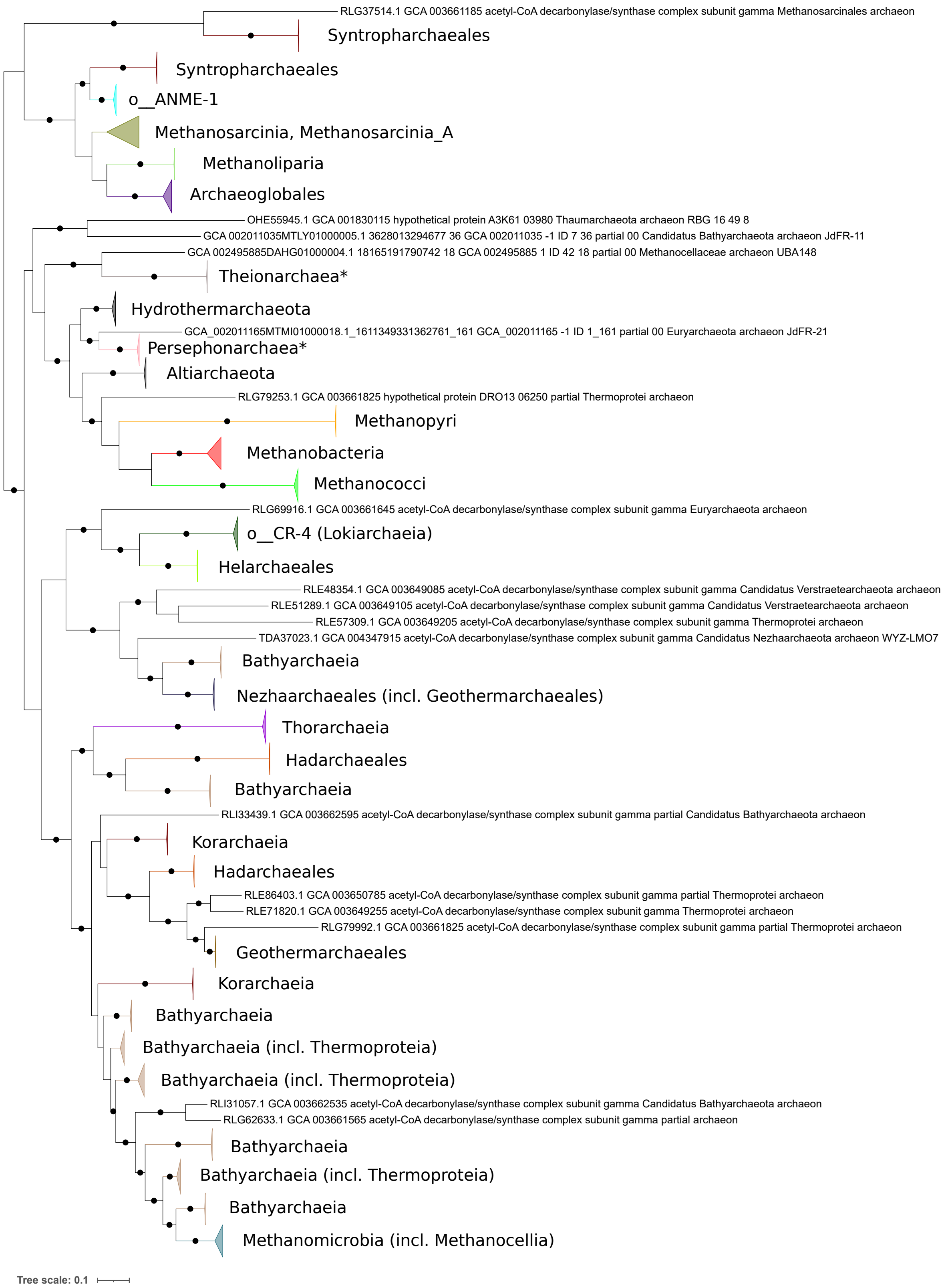

Fig. S9.
ML phylogeny of CdhE. Black circles indicate strongly supported branches (ultrafast bootstrap >=95, aLRT SH-like >=80).

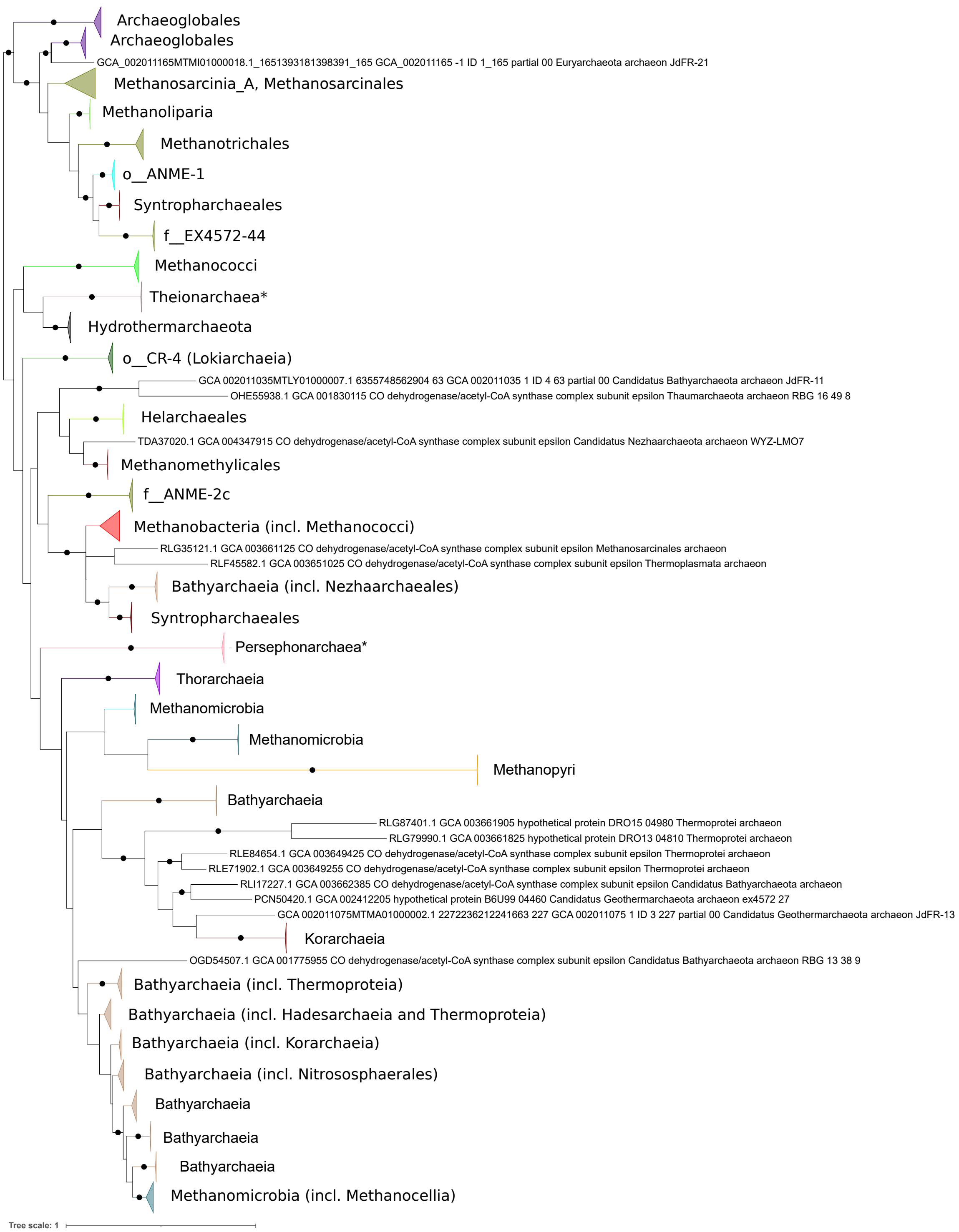

Fig. S10.
ML phylogeny of CdhB. Black circles indicate strongly supported branches (ultrafast bootstrap >=95, aLRT SH-like >=80).

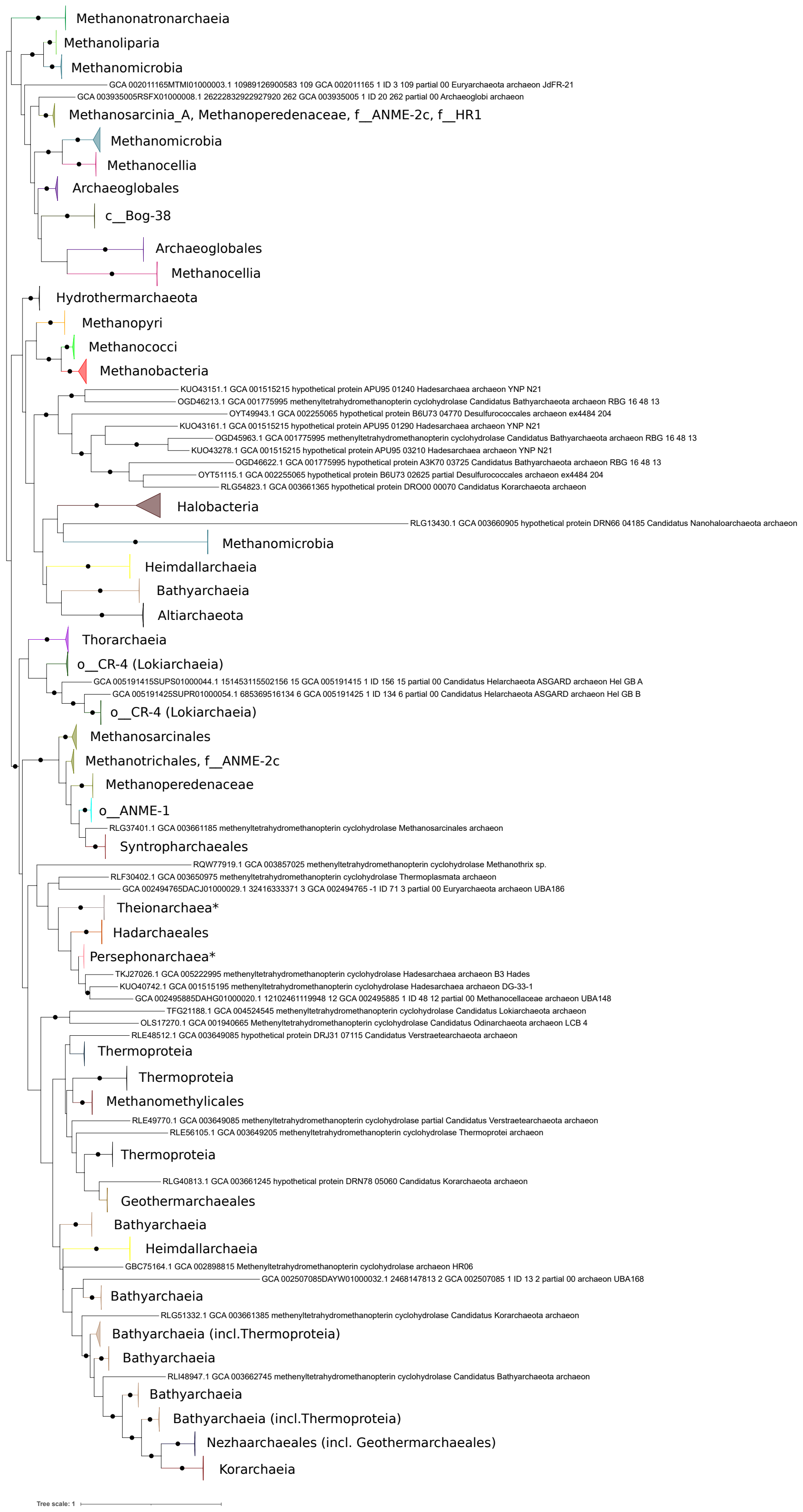

Fig. S11.
ML phylogeny of Mch. Black circles indicate strongly supported branches (ultrafast bootstrap >=95, aLRT SH-like >=80).

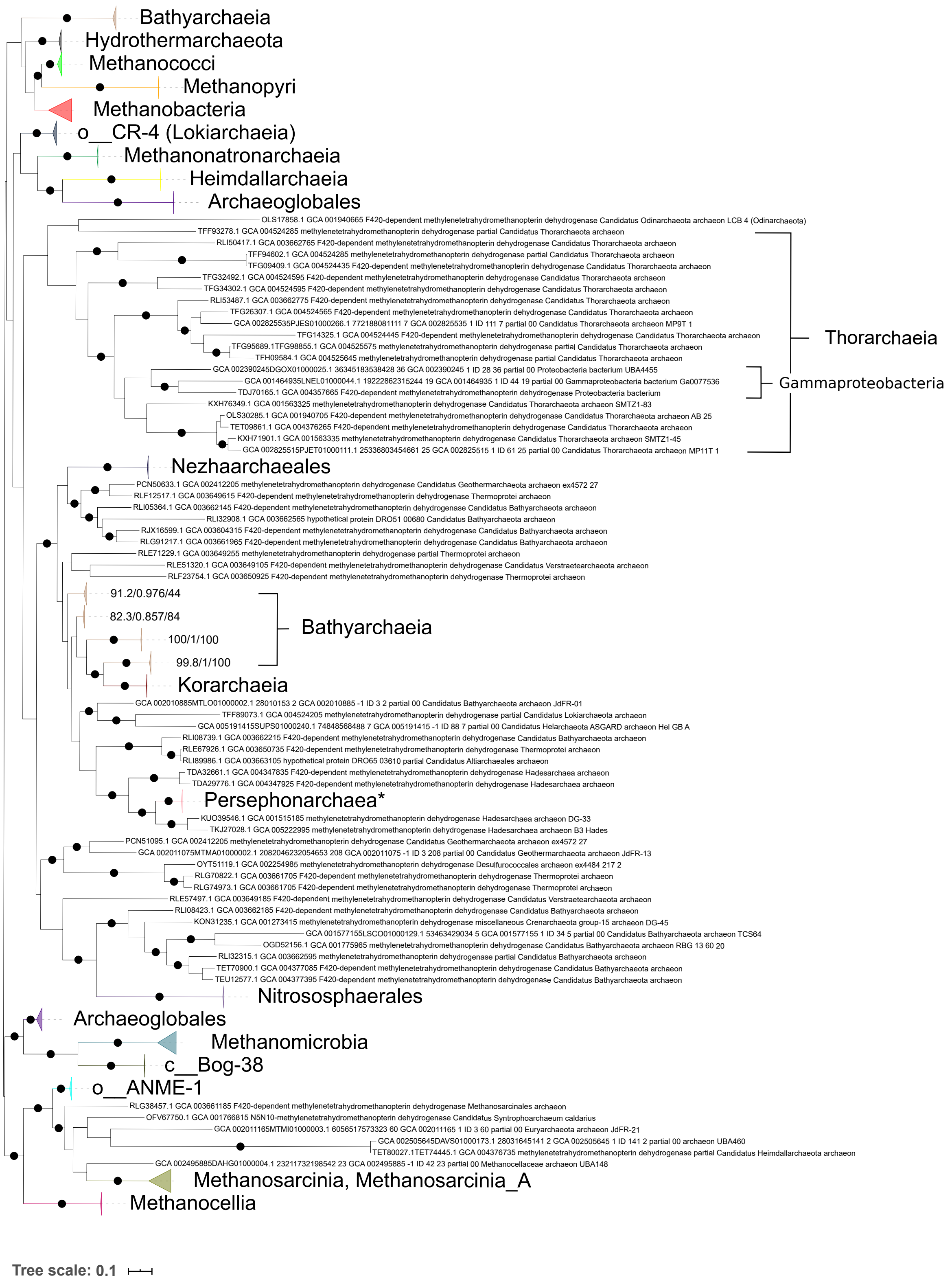

Fig. S12.
ML phylogeny of Mtd. Black circles indicate strongly supported branches (ultrafast bootstrap >=95, aLRT SH-like >=80).

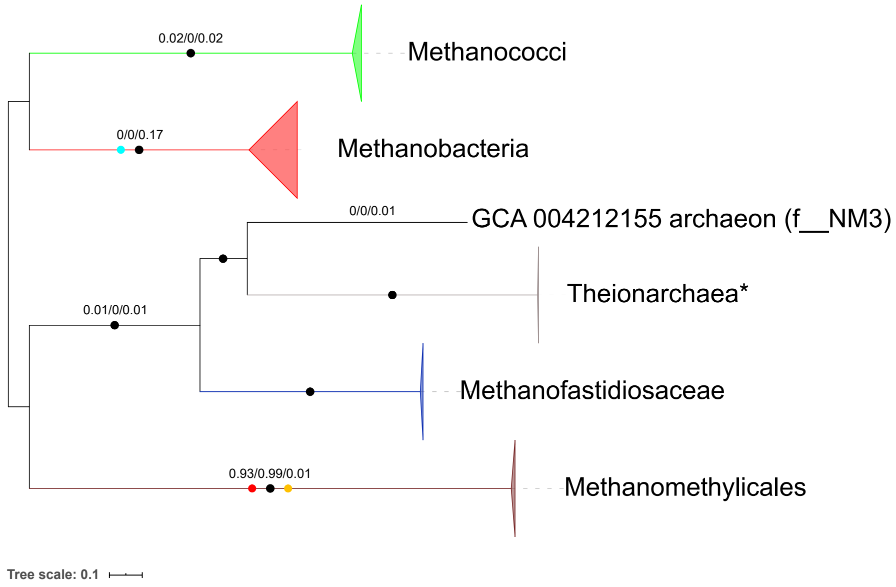

Fig. S13.
ML phylogeny of EhbEFGHIKLMO (1914 aa positions). Black circles indicate strongly supported branches (ultrafast bootstrap >=95, aLRT SH-like >=80), red circle corresponds to the MAD root, orange to MinVar, light blue to NONREV. Branch values correspond to rootstrap supports for MAD, MinVar, and NONREV respectively.

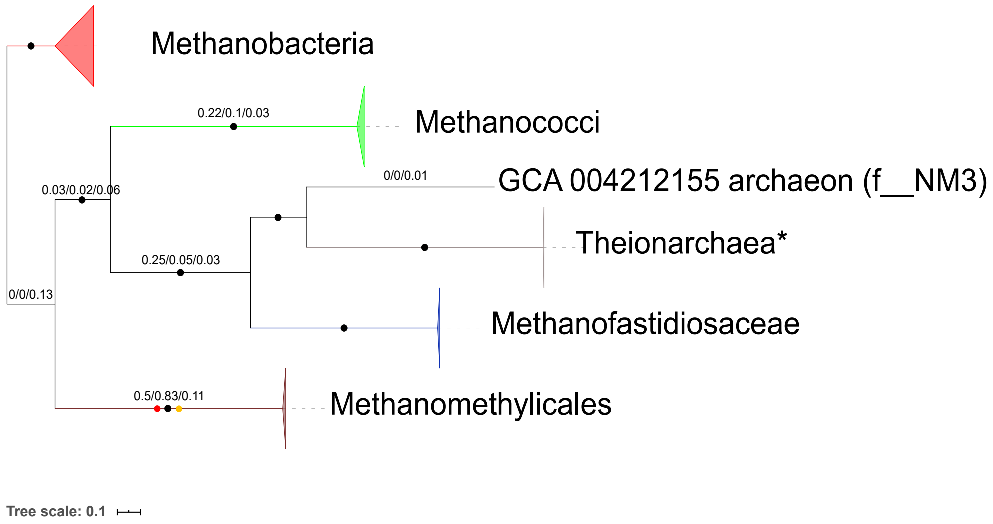

Fig. S14.
ML phylogeny of EhbEGHIKLM (1135 aa positions). Black circles indicate strongly supported branches (ultrafast bootstrap >=95, aLRT SH-like >=80), red circle corresponds to the MAD root, orange to MinVar. Branch values correspond to rootstrap supports for MAD, MinVar, and NONREV respectively. The NONREV root is within a collapsed clade.

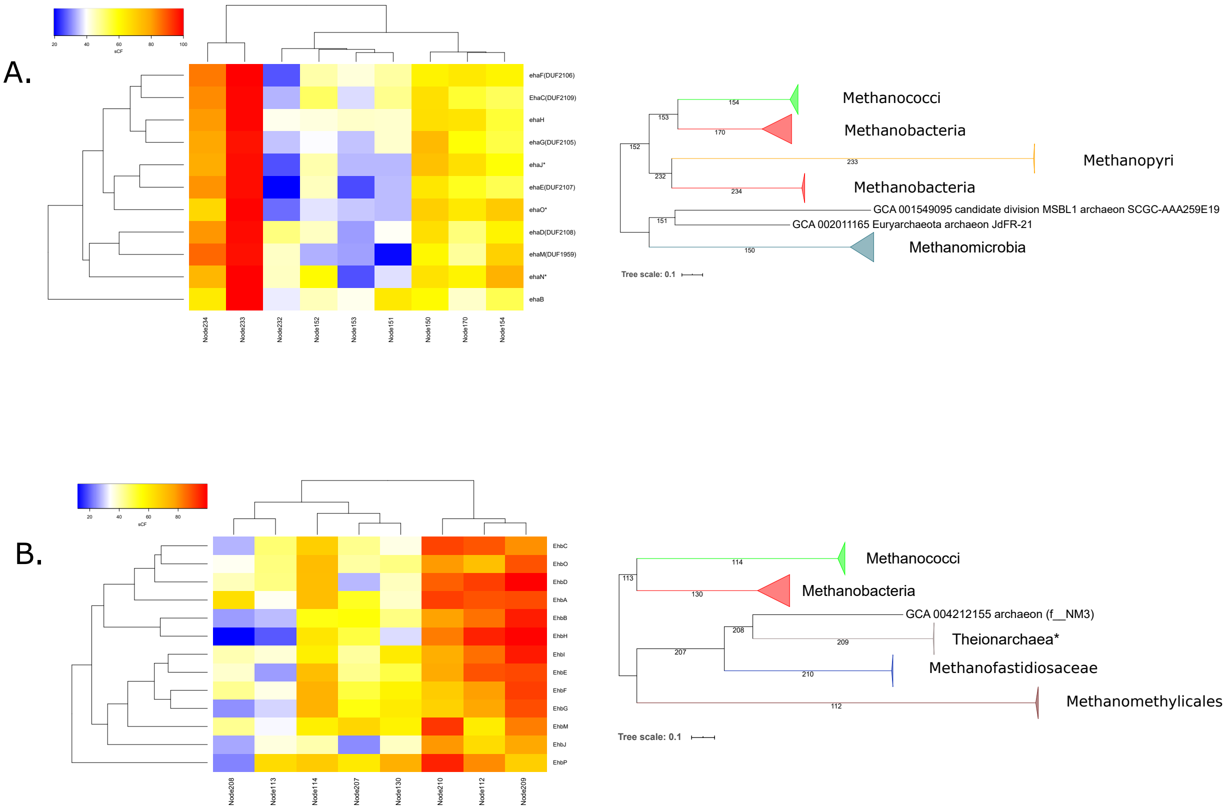

A.
ehaF(DUF2106)
EhaC(DUF2109)
ehaH
ehaG(DUF2105)
ehaJ*
ehaE(DUF2107)
ehaO*
ehaD(DUF2108)
ehaM(DUF1959)
ehaN*
ehaB
Node234
Node233
Node232
Node152
Node153
Node151
Node150
Node170
Node154
Methanococci
Methanobacteria
Methanopyri
Methanobacteria
GCA 001549095 candidate division MSBL1 archaeon SCGC-AAA259E19
GCA 002011165 Euryarchaeota archaeon JdFR-21
Methanomicrobia
Tree scale: 0.1
B.
EhbC
EhbO
EhbD
EhbA
EhbB
EhbH
EhbI
EhbE
EhbF
EhbG
EhbM
EhbJ
EhbP
Node208
Node113
Node114
Node207
Node130
Node210
Node112
Node209
Methanococci
Methanobacteria
GCA 004212155 archaeon (f__NM3)
Theionarchaea*
Methanofastidiosaceae
Methanomethylicales
Tree scale: 0.1

C.

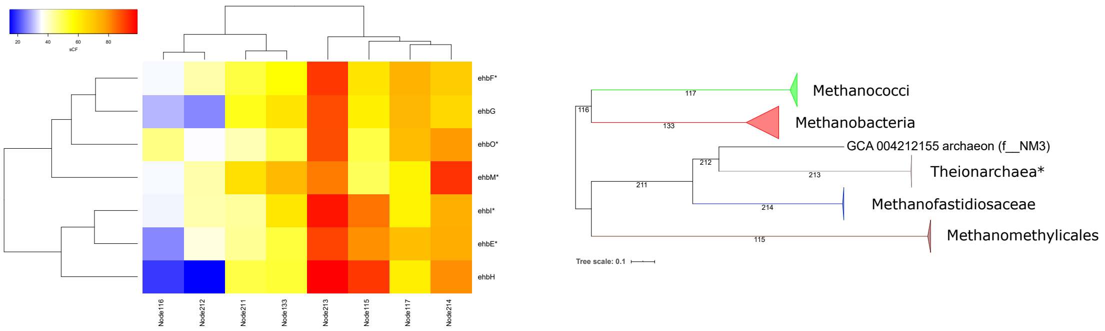

sCF
ehbF*
ehbG
ehbO*
ehbM*
ehbI*
ehbE*
ehbH
Node116
Node212
Node211
Node133
Node213
Node115
Node117
Node214
Methanococci
Methanobacteria
GCA 004212155 archaeon (f__NM3)
Theionarchaea*
Methanofastidiosaceae
Methanomethylicales
Tree scale: 0.1

D.

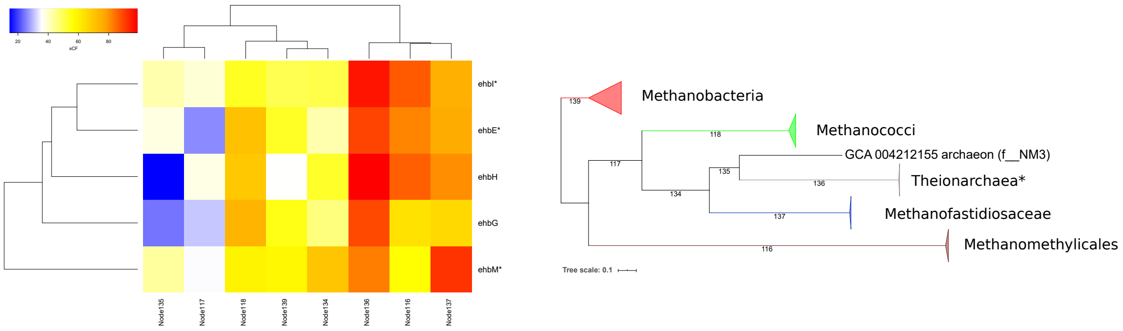

sCF
ehbI*
ehbE*
ehbH
ehbG
ehbM*
Node135
Node117
Node118
Node139
Node134
Node136
Node116
Node137
Methanobacteria
Methanococci
GCA 004212155 archaeon (f__NM3)
Theionarchaea*
Methanofastidiosaceae
Methanomethylicales
Tree scale: 0.1

E.

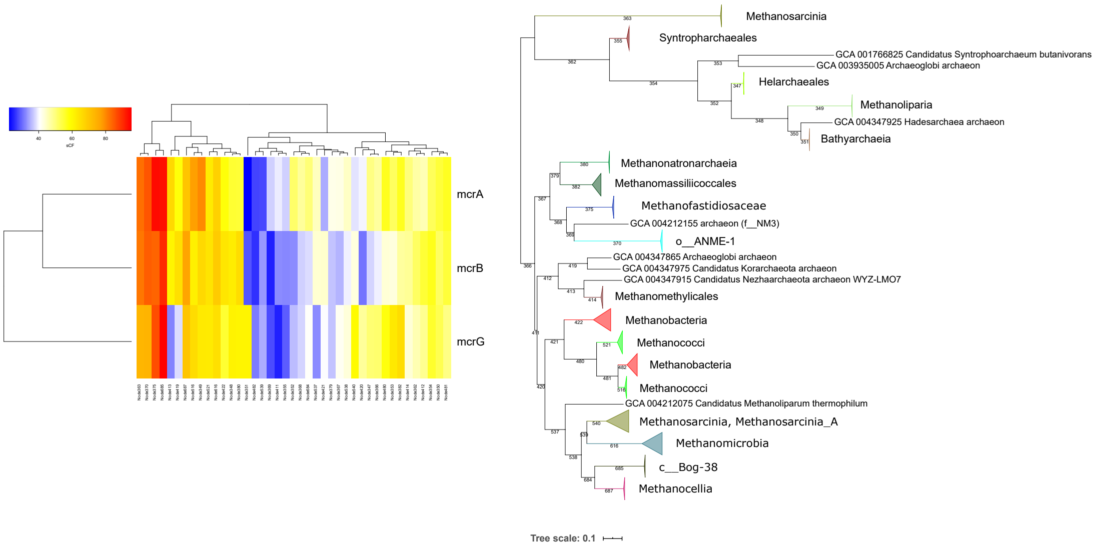

F.

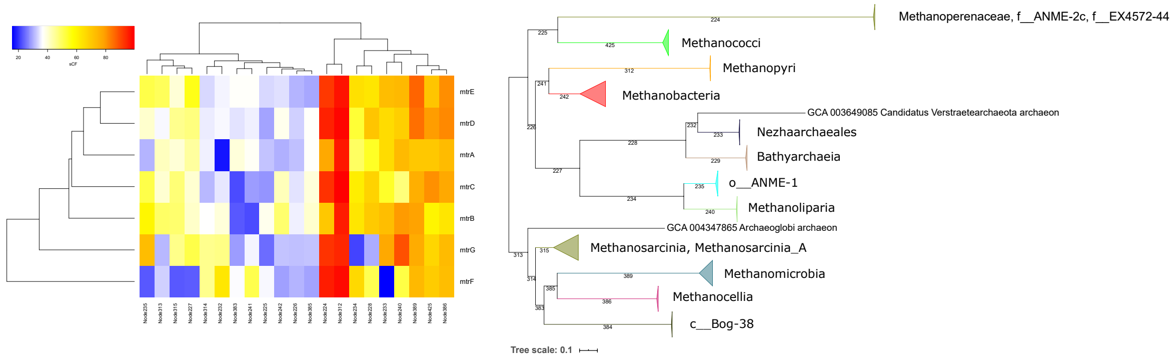

Fig. S15.
Heatmap graphs of subunit-specific site concordance factors for the main clades in the phylogenies of (**A**) EhaBCDEFGHJMNO, (**B**) EhbABCDEFGHIJMOP, (**C**) EhbEFGHIMO, (**D**) EhbEGHIM, (**E**) McrABG, (**F**) MtrABCDEFG. Cladograms were derived from Euclidean distance clustering. Subunits noted with asterisks have known homologs in other hydrogenases (generic subunits). Some subunits are missing from the sCF analyses, as the concatenation dataset needs to, in terms of distribution, perfectly match the datasets of the single subunits. In case major clades of the concatenation phylogenies were absent in the single subunit phylogenies, those subunits were omitted from the analysis.

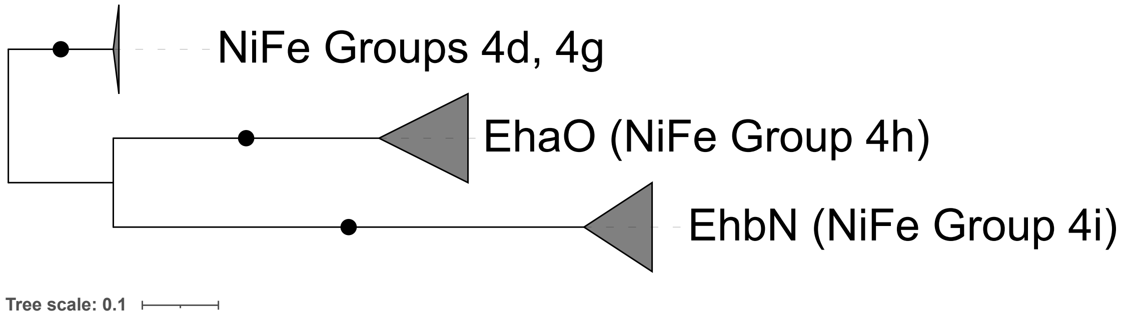

Fig. S16.
ML phylogeny of EhaO-EhbN, with an outgroup of NiFe groups 4d and 4g hydrogenases. Black circles indicate strongly supported branches (ultrafast bootstrap >=95, aLRT SH-like >=80).

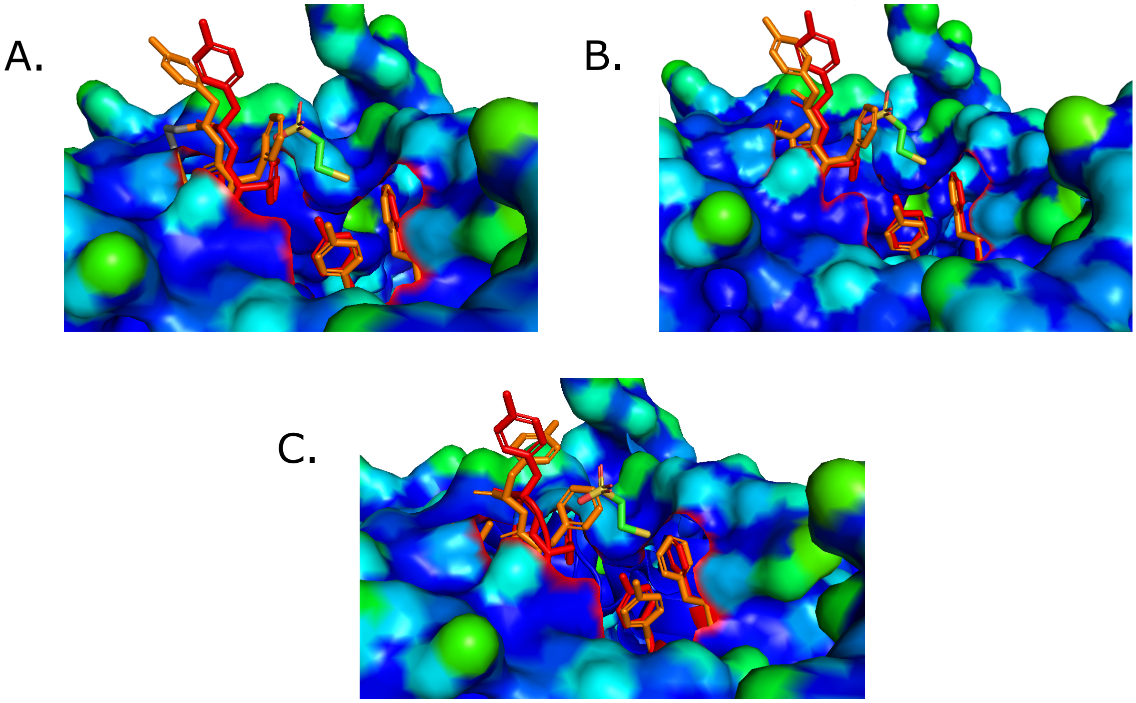

Fig. S17.
Homology model of the ancestral McrA sequence (orange) with the (**A**) Mcr-like/Acr, (**B**) Halobacteriota, (**C**) Thermoproteota roots aligned with the template structure of the *M. marburgensis* McrA (PDB ID: 1HBU, chain D; red). The view is focused on the methyl-CoM binding cavity.

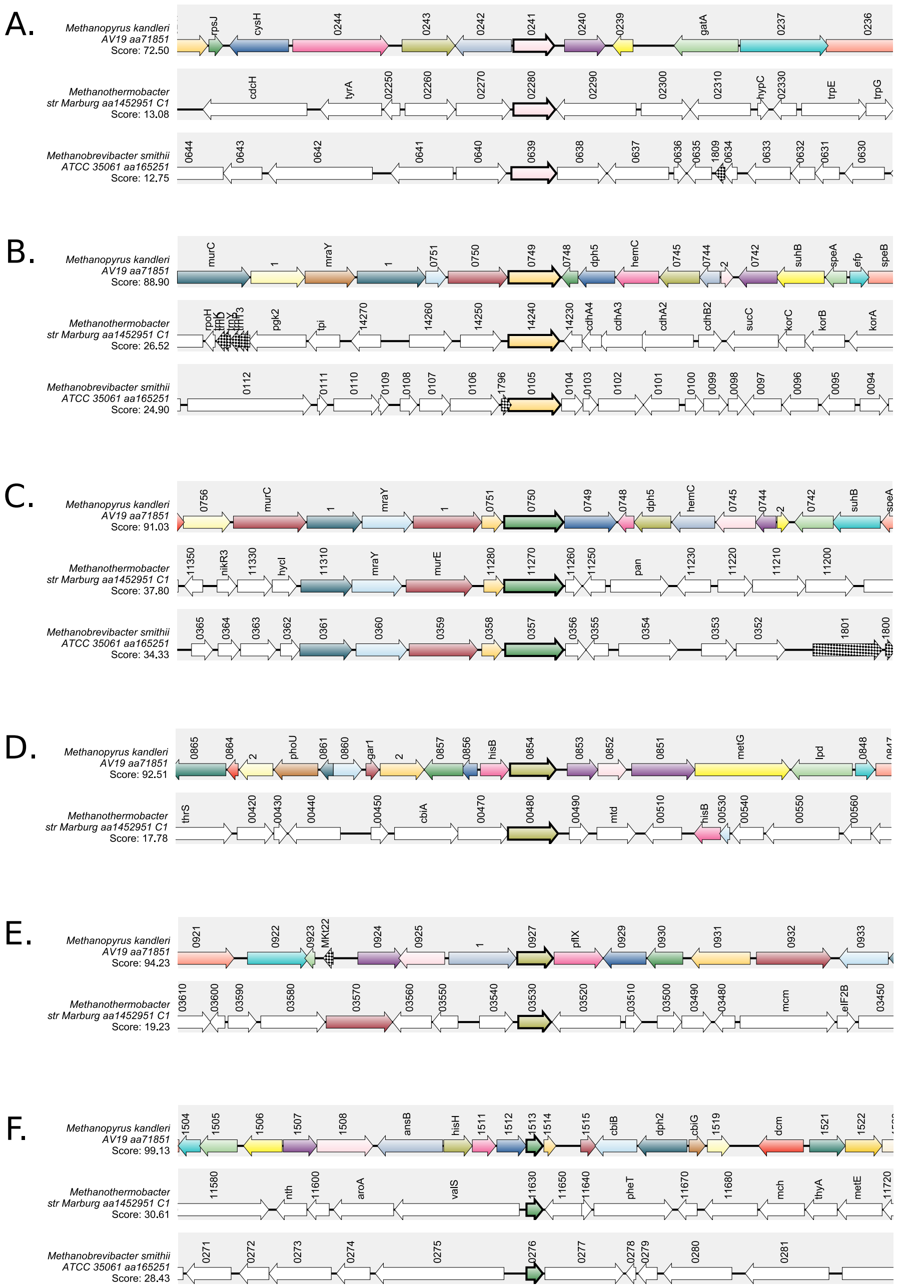

Fig. S18.

Graphical representation of synteny for genes (**A**) MK0241, (**B**) MK0749, (**C**) MK0750 (includes MK0751), (**D**) MK0854, (**E**) MK0927, and (**F**) MK1513 in the genomes of *Methanopyrus kandleri*, *Methanothermobacter marburgensis str. Marburg*, and *Methanobrevibacter smithii*. Other than MK0750 and MK0751, MK0854 is annotated as belonging in the MurG family and MK0927 is syntenic to a MurE-like protein in *M. kandleri*. For the synteny analysis we used the SyntTax server (*Methanopyrus kandleri* AV19 sequences as queries, *M. kandleri* AV19, *M. marburgensis* str. Marburg, and *Methanobrevibacter smithii* ATCC 35061 as selected chromosomes, best match, default minimal score)(*141*).

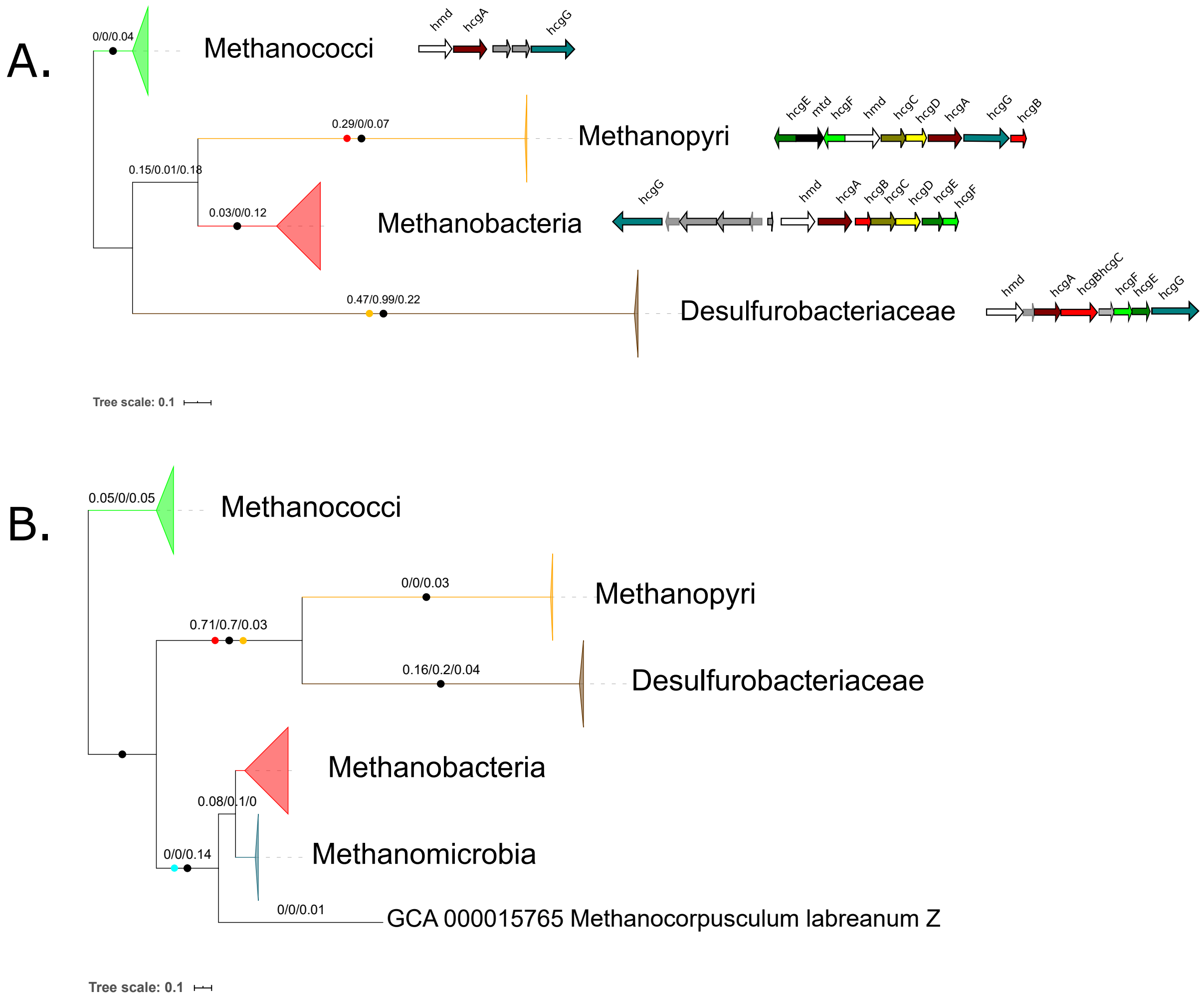

Fig. S19.
ML phylogenies of (**A**) HcgAEFG (938 aa positions) along with the genomic organization of the clusters in a representative genome for each major clade, (**B**) HcgBC (366 aa positions). Black circles indicate strongly supported branches (ultrafast bootstrap >=95, aLRT SH-like >=80), red circles correspond to the MAD root, orange to MinVar, light blue to NONREV. Branch values correspond to rootstrap supports for MAD, MinVar, and NONREV respectively. For HcgAEFG, the NONREV root is within a collapsed clade. Genomes used to compare clusters were the same as Fig. 3, with additionally *Desulfurobacterium thermolithotrophum* DSM 11699 (GCA_000191045; Desulfurobacteriales).

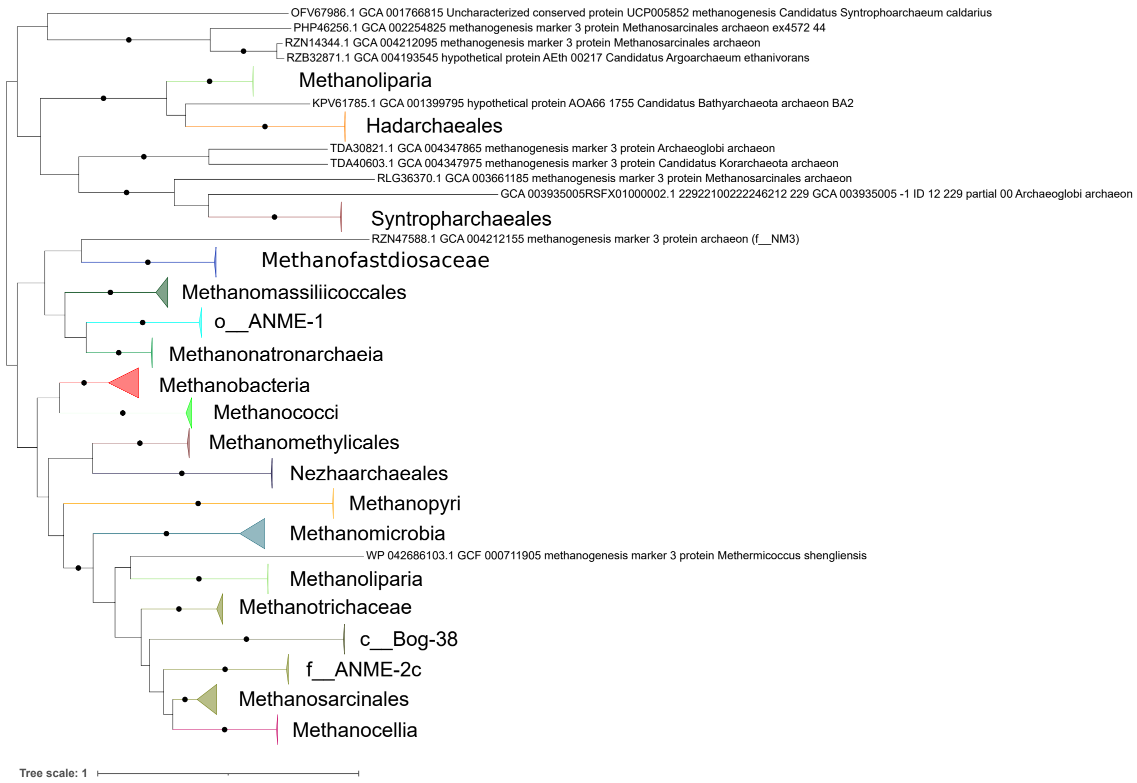

Fig. S20.
ML phylogeny of methanogenesis marker m4. Black circles indicate strongly supported branches (ultrafast bootstrap >=95, aLRT SH-like >=80).

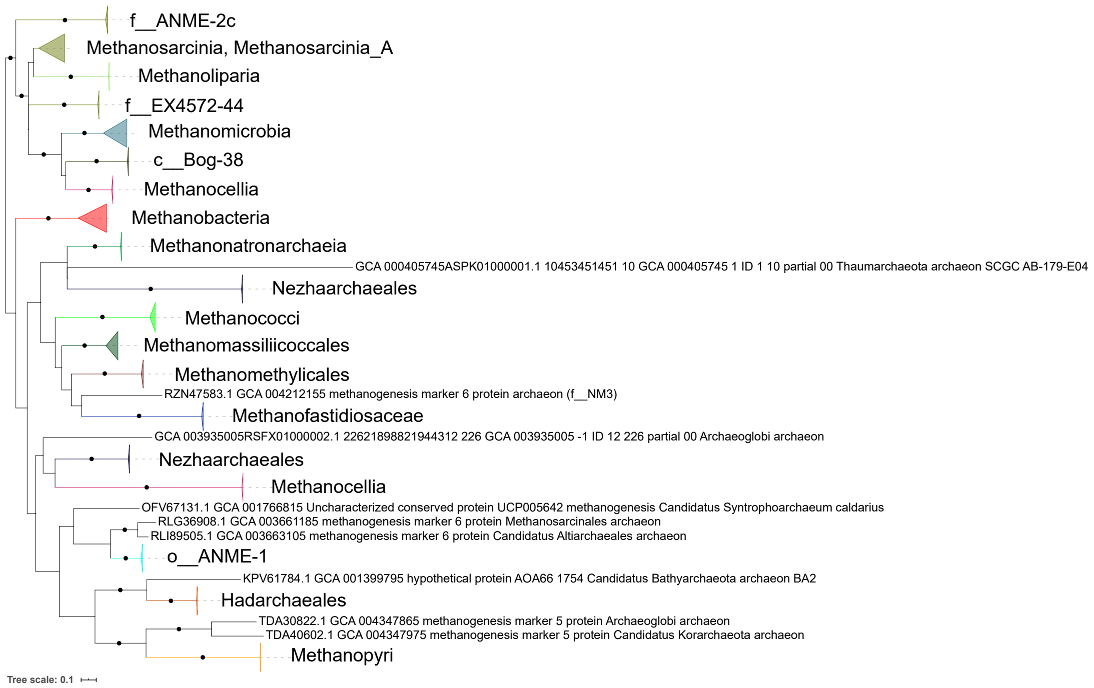

Fig. S21.
ML phylogeny of methanogenesis marker m5. Black circles indicate strongly supported branches (ultrafast bootstrap >=95, aLRT SH-like >=80).

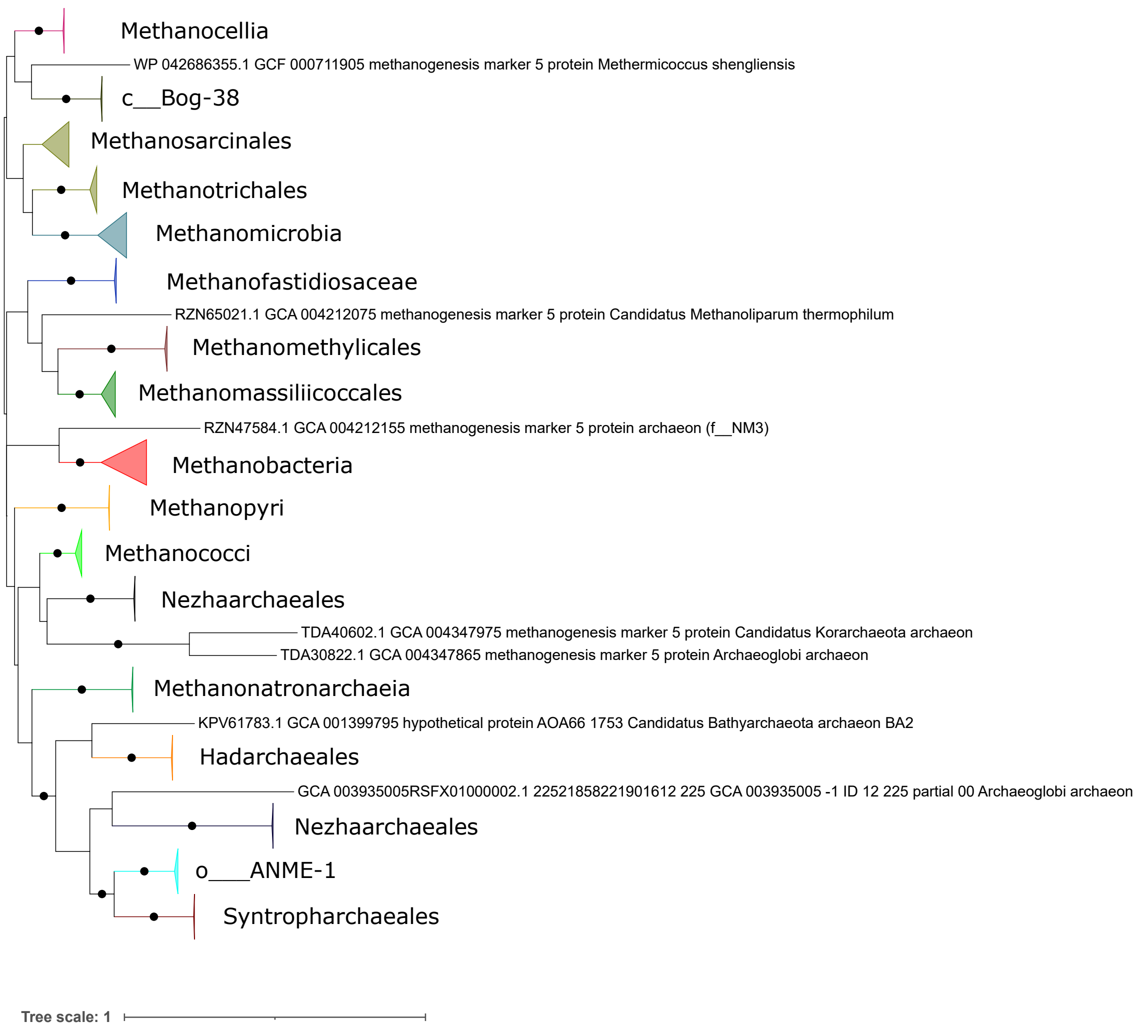

Fig. S22.
ML phylogeny of methanogenesis marker m6. Black circles indicate strongly supported branches (ultrafast bootstrap >=95, aLRT SH-like >=80).

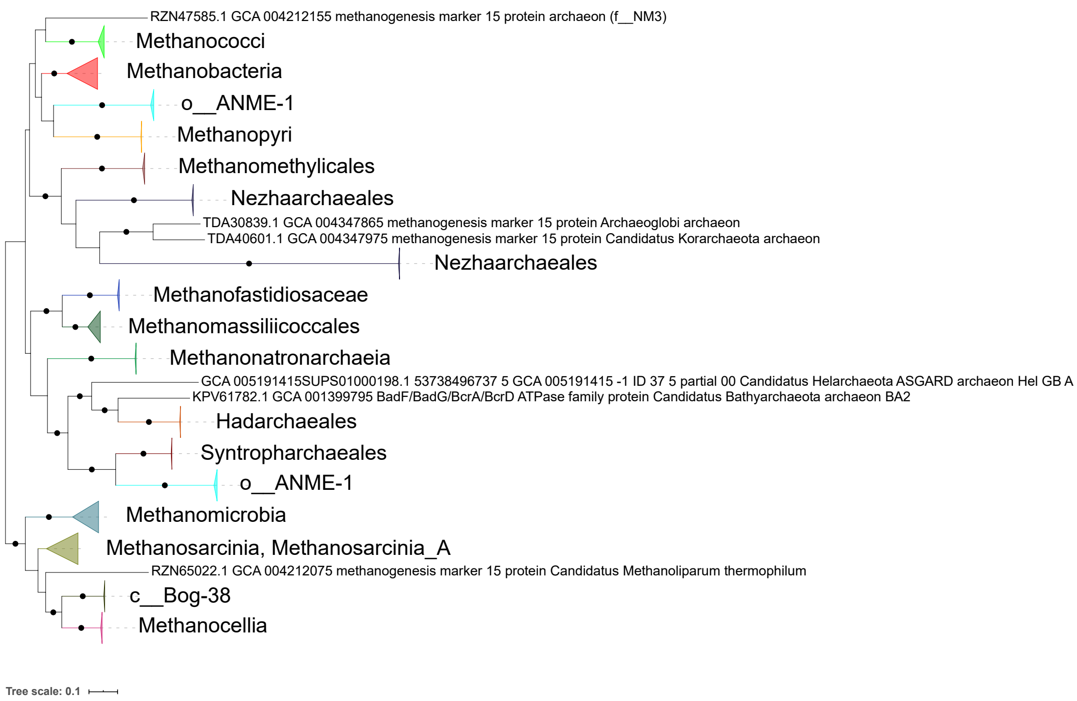

Fig. S23.
ML phylogeny of methanogenesis marker m7. Black circles indicate strongly supported branches (ultrafast bootstrap >=95, aLRT SH-like >=80).

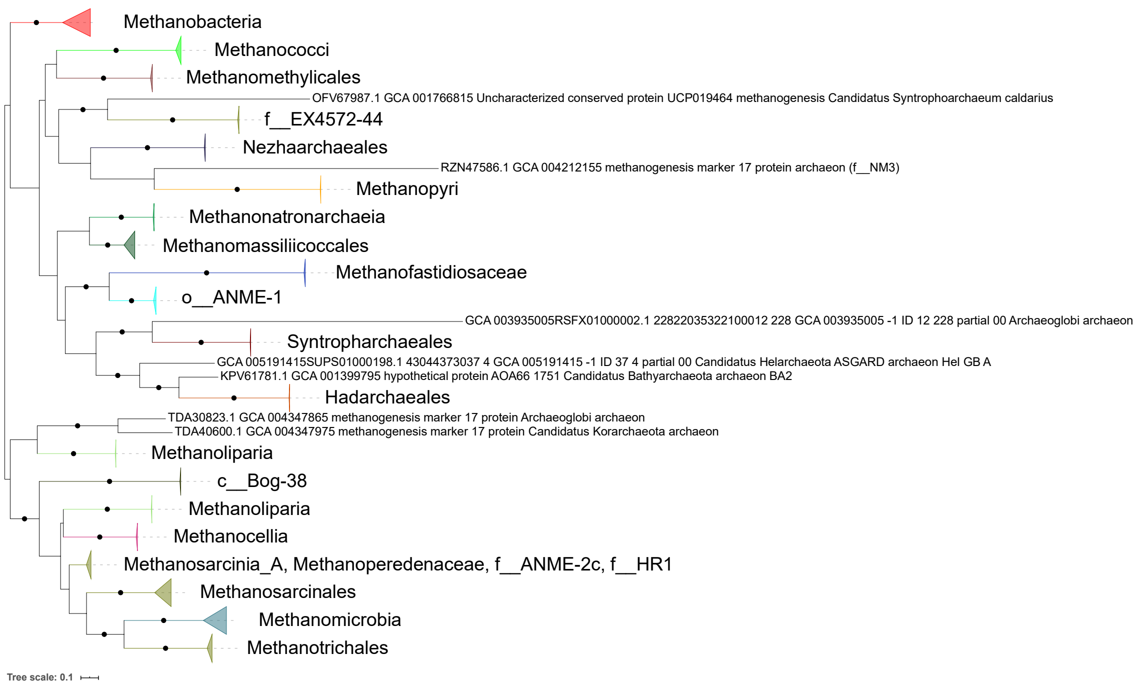

Fig. S24.
ML phylogeny of methanogenesis marker m8. Black circles indicate strongly supported branches (ultrafast bootstrap >=95, aLRT SH-like >=80).

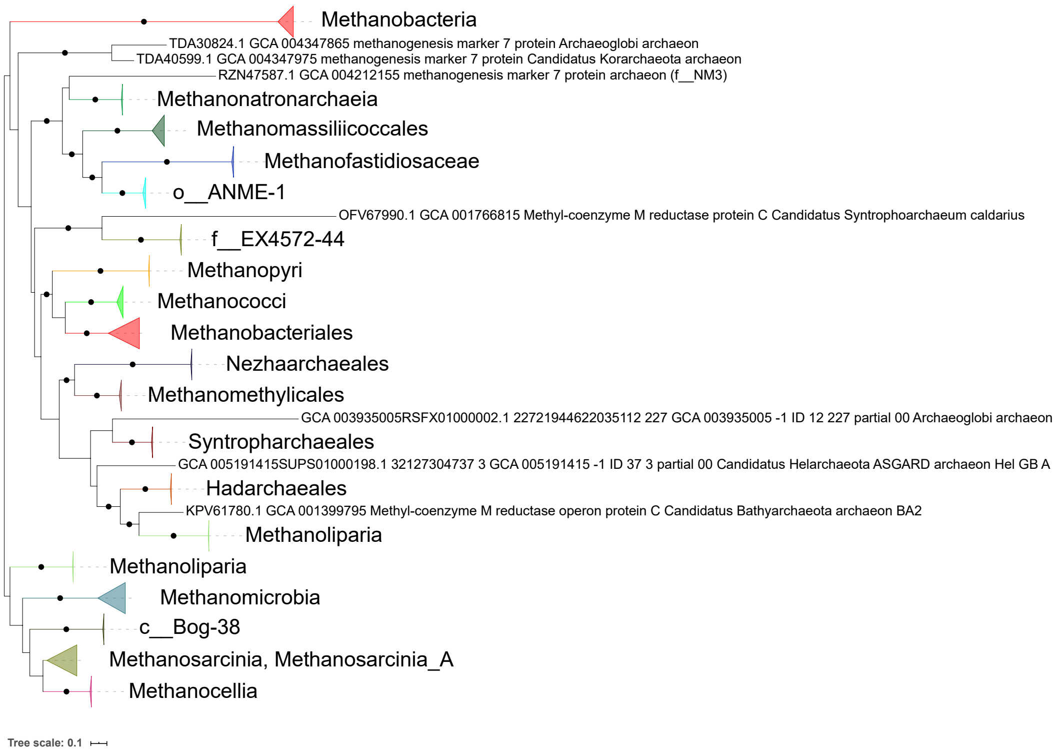

Fig. S25.
ML phylogeny of methanogenesis marker m9. Black circles indicate strongly supported branches (ultrafast bootstrap >=95, aLRT SH-like >=80).

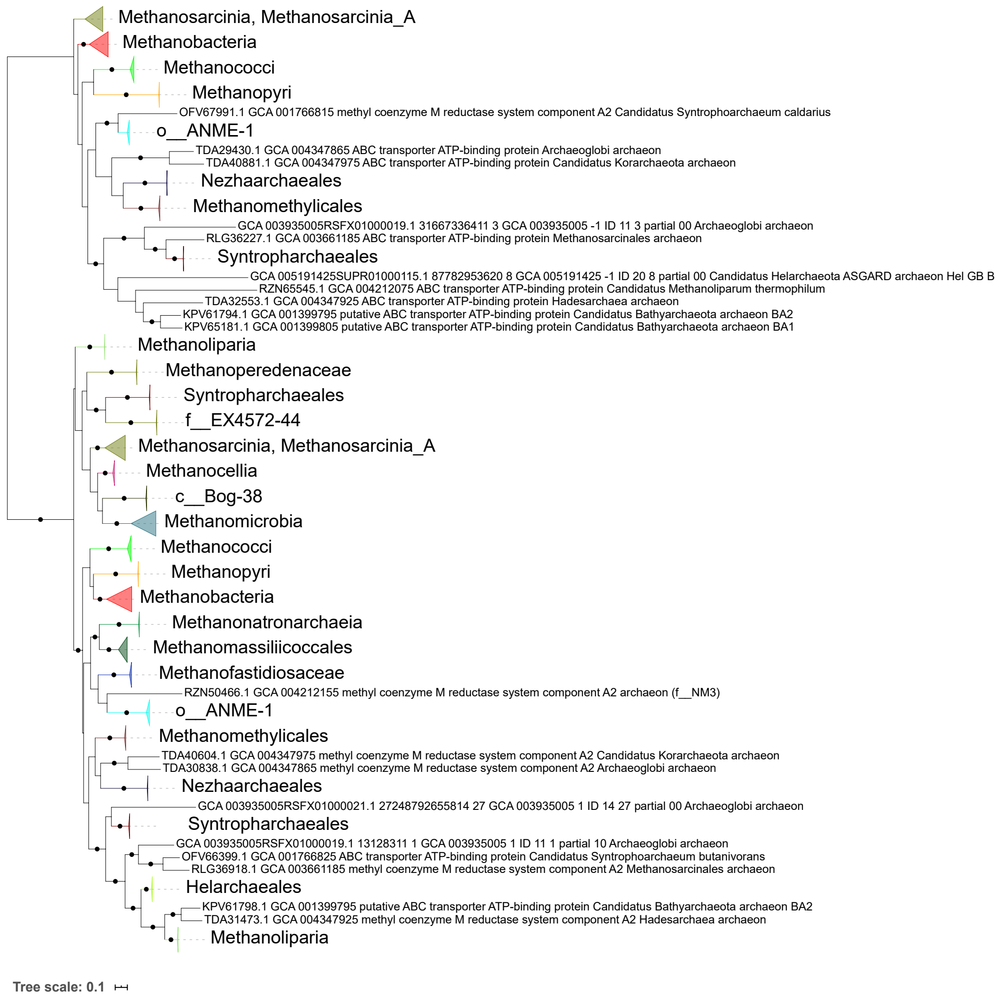

Fig. S26.

ML phylogeny of methanogenesis marker m10. Black circles indicate strongly supported branches (ultrafast bootstrap >=95, aLRT SH-like >=80).

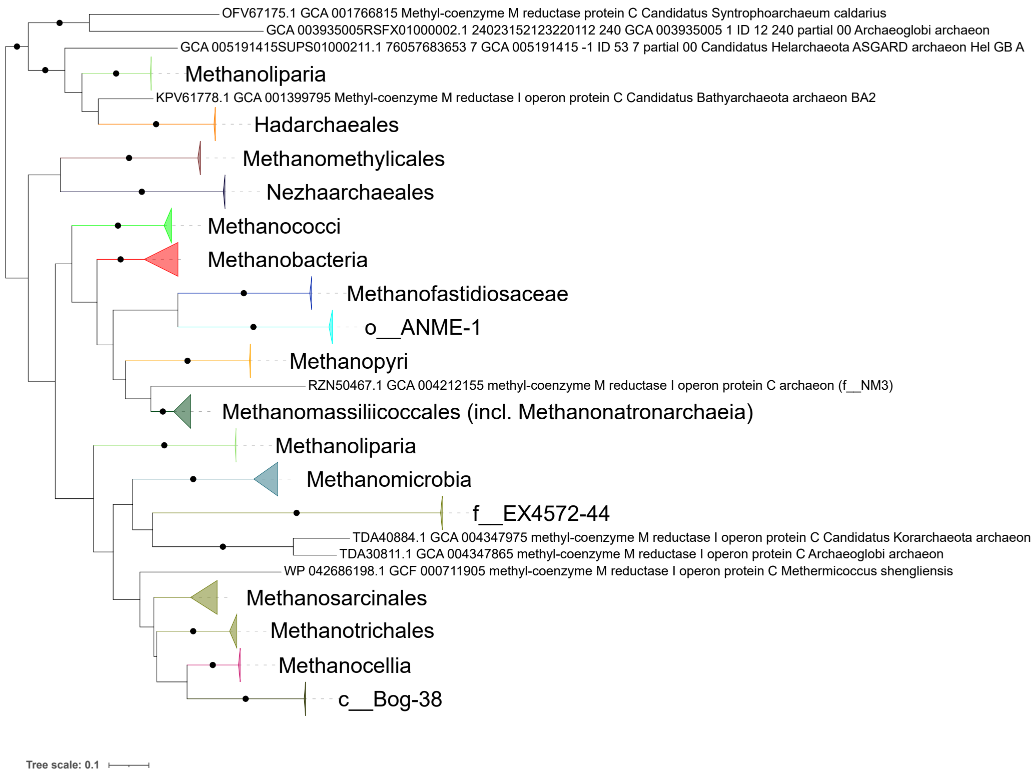

Fig. S27.

ML phylogeny of methanogenesis marker m11. Black circles indicate strongly supported branches (ultrafast bootstrap >=95, aLRT SH-like >=80).

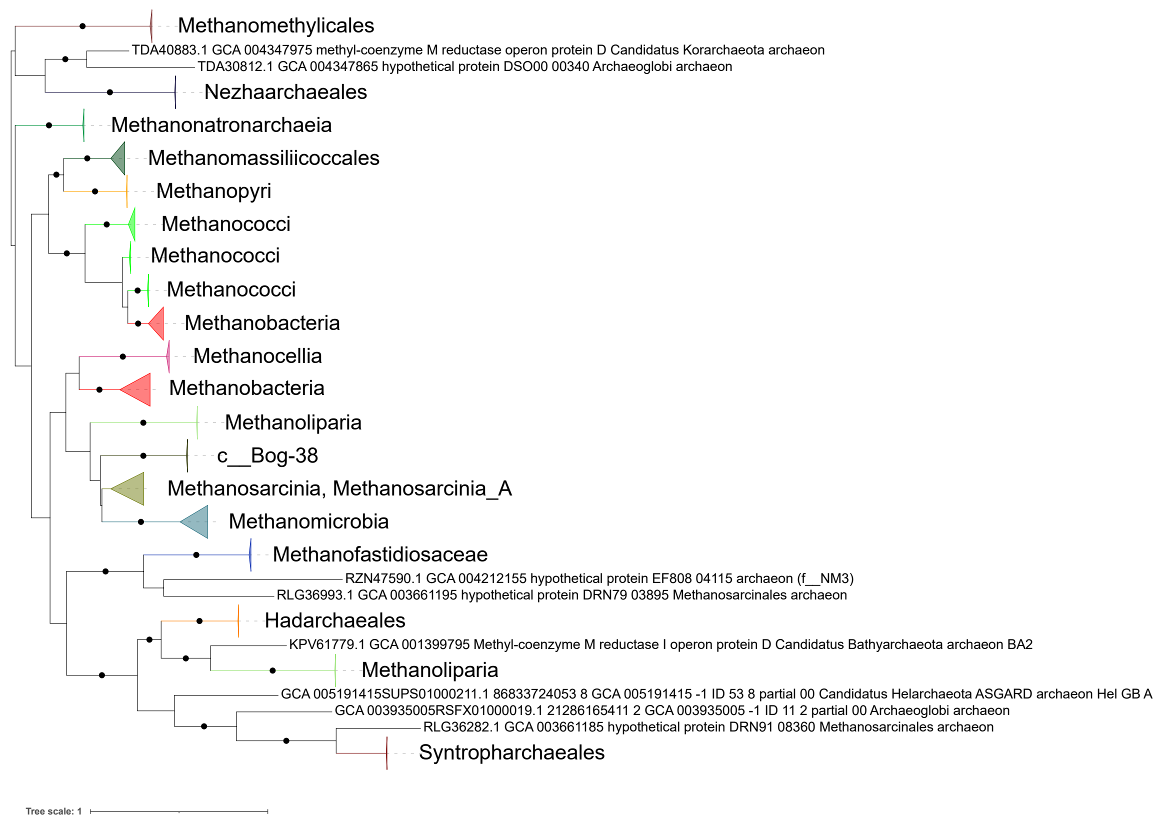

Fig. S28.
ML phylogeny of methanogenesis marker m12. Black circles indicate strongly supported branches (ultrafast bootstrap >=95, aLRT SH-like >=80).

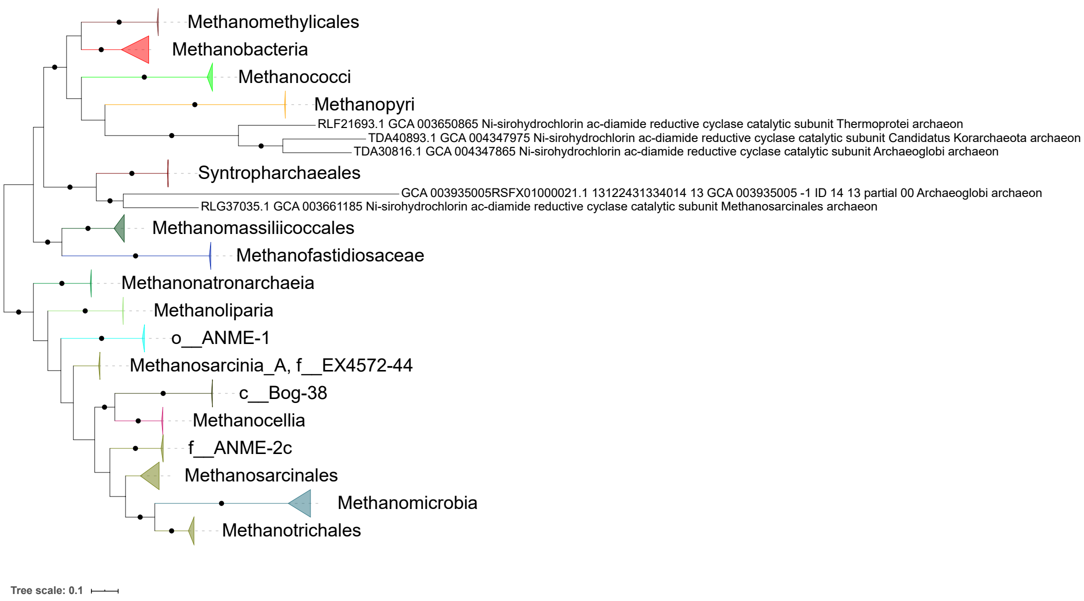

Fig. S29.
ML phylogeny of methanogenesis marker m13. Black circles indicate strongly supported branches (ultrafast bootstrap >=95, aLRT SH-like >=80).

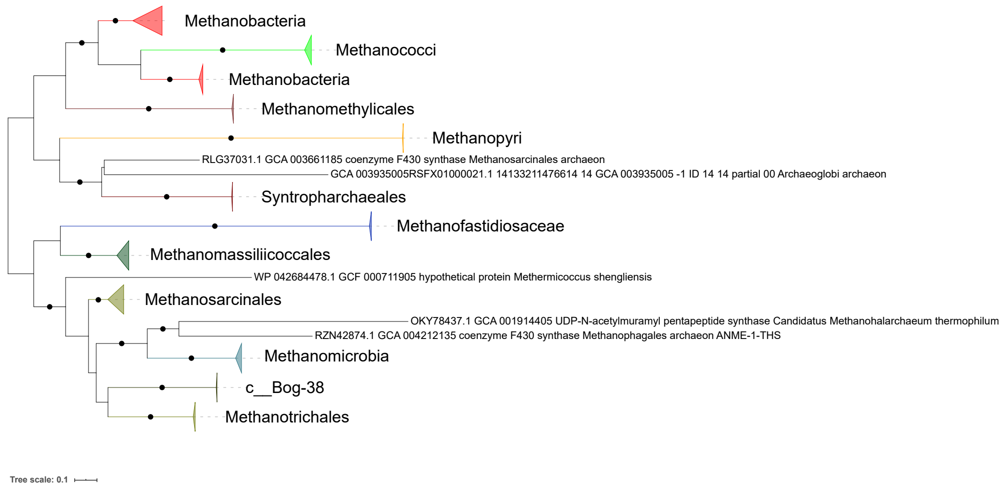

Fig. S30.
ML phylogeny of methanogenesis marker m14. Black circles indicate strongly supported branches (ultrafast bootstrap >=95, aLRT SH-like >=80).

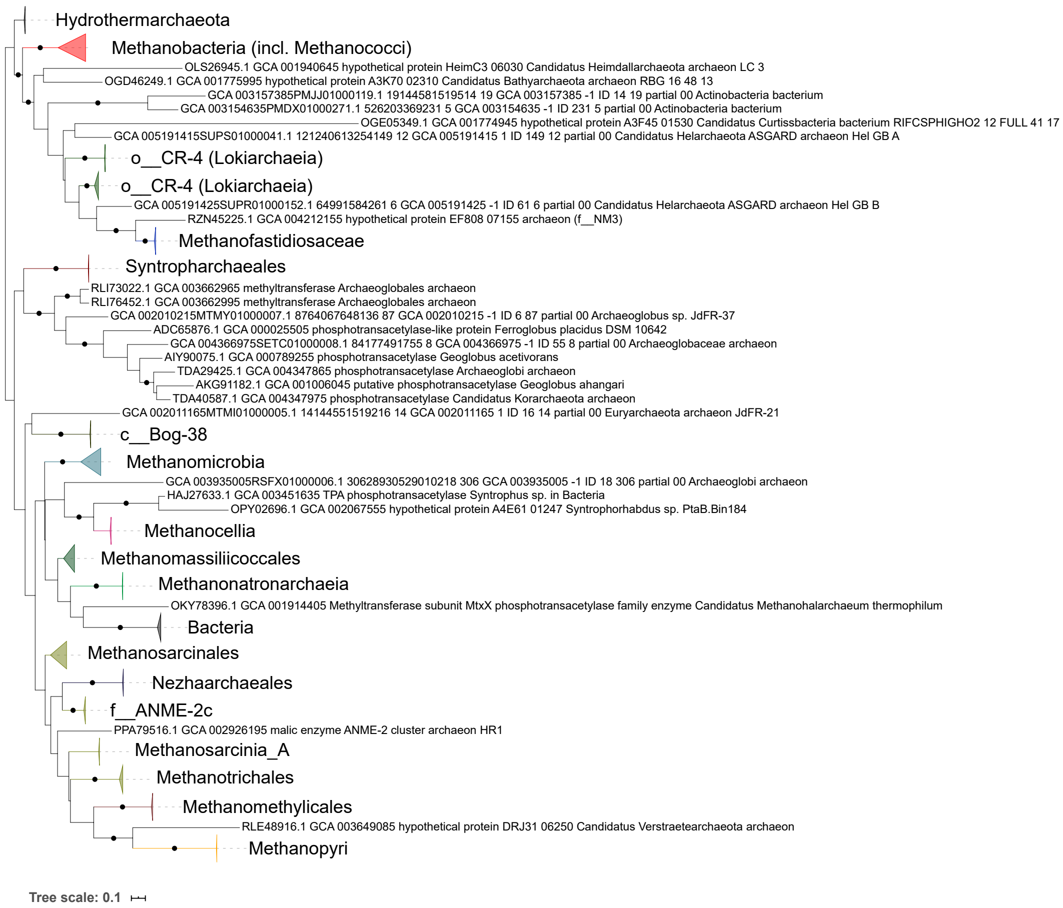

Fig. S31.
ML phylogeny of methanogenesis marker m15. Black circles indicate strongly supported branches (ultrafast bootstrap >=95, aLRT SH-like >=80).

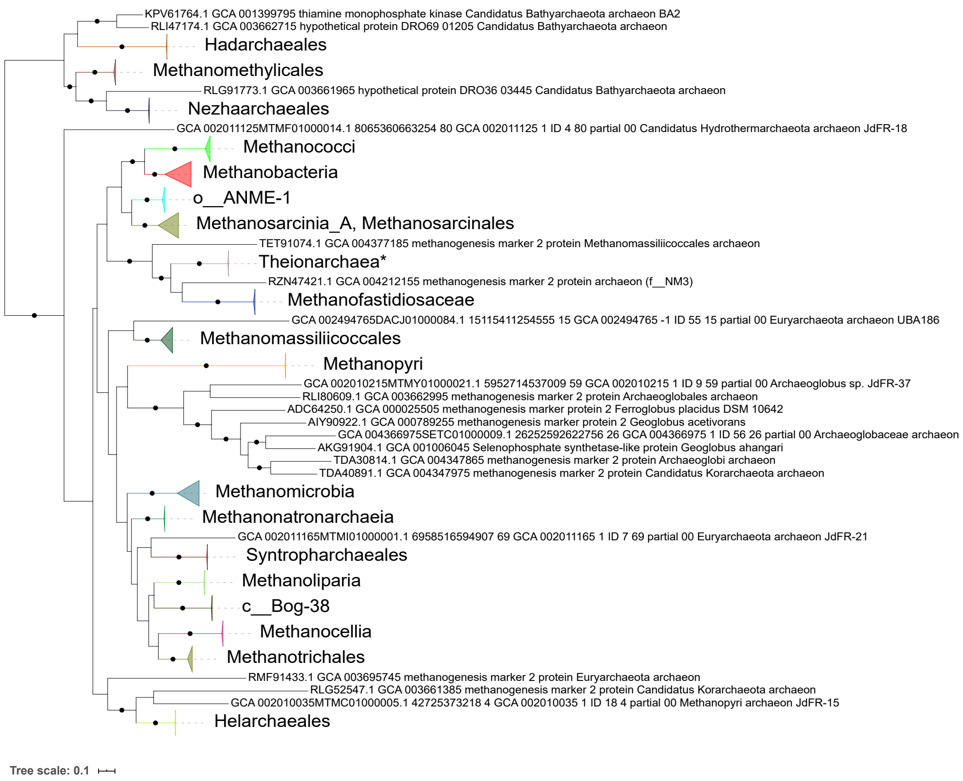

Fig. S32.
ML phylogeny of methanogenesis marker m16. Black circles indicate strongly supported branches (ultrafast bootstrap >=95, aLRT SH-like >=80).

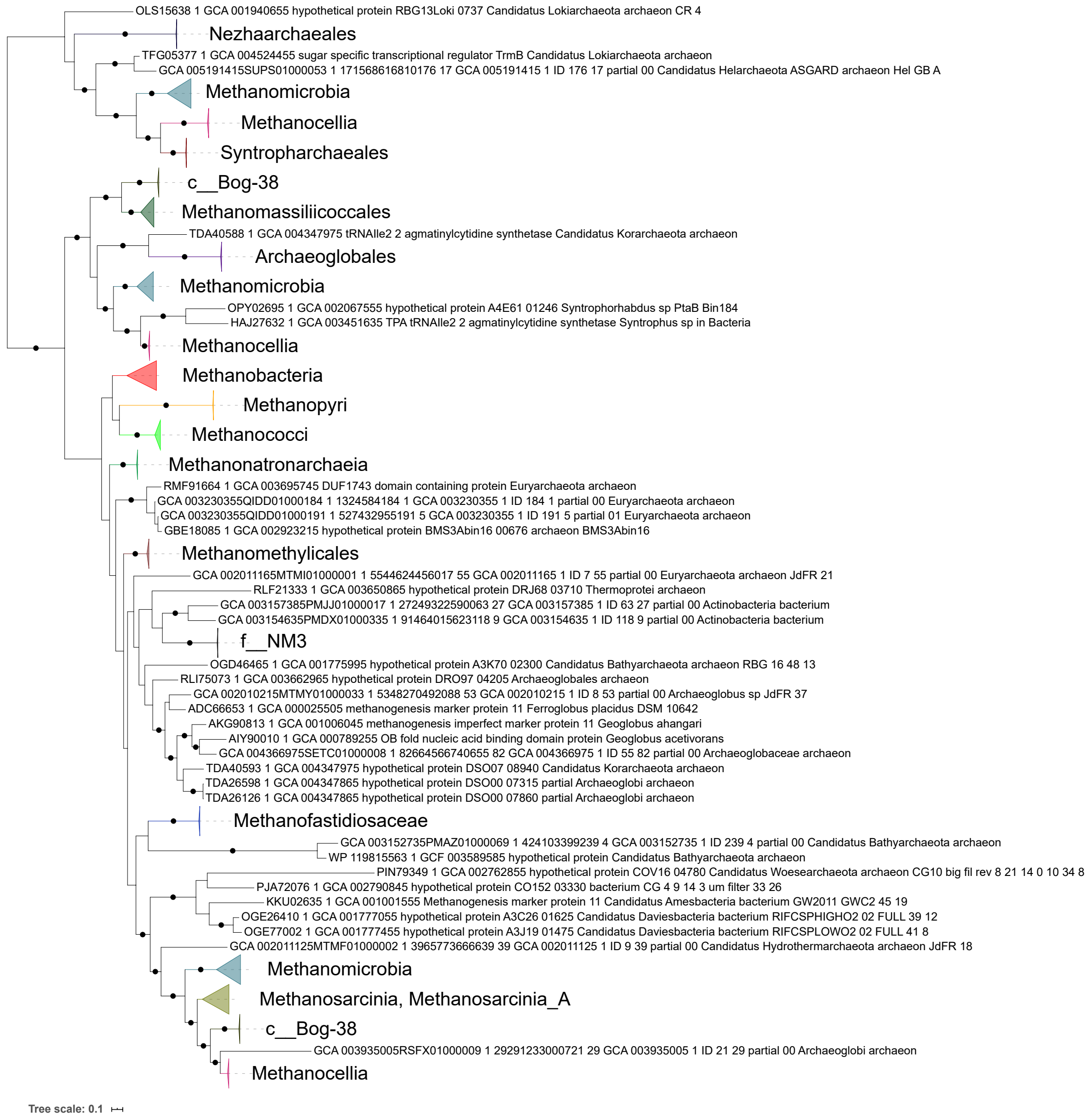

Fig. S33.
ML phylogeny of methanogenesis marker m17. Black circles indicate strongly supported branches (ultrafast bootstrap >=95, aLRT SH-like >=80).

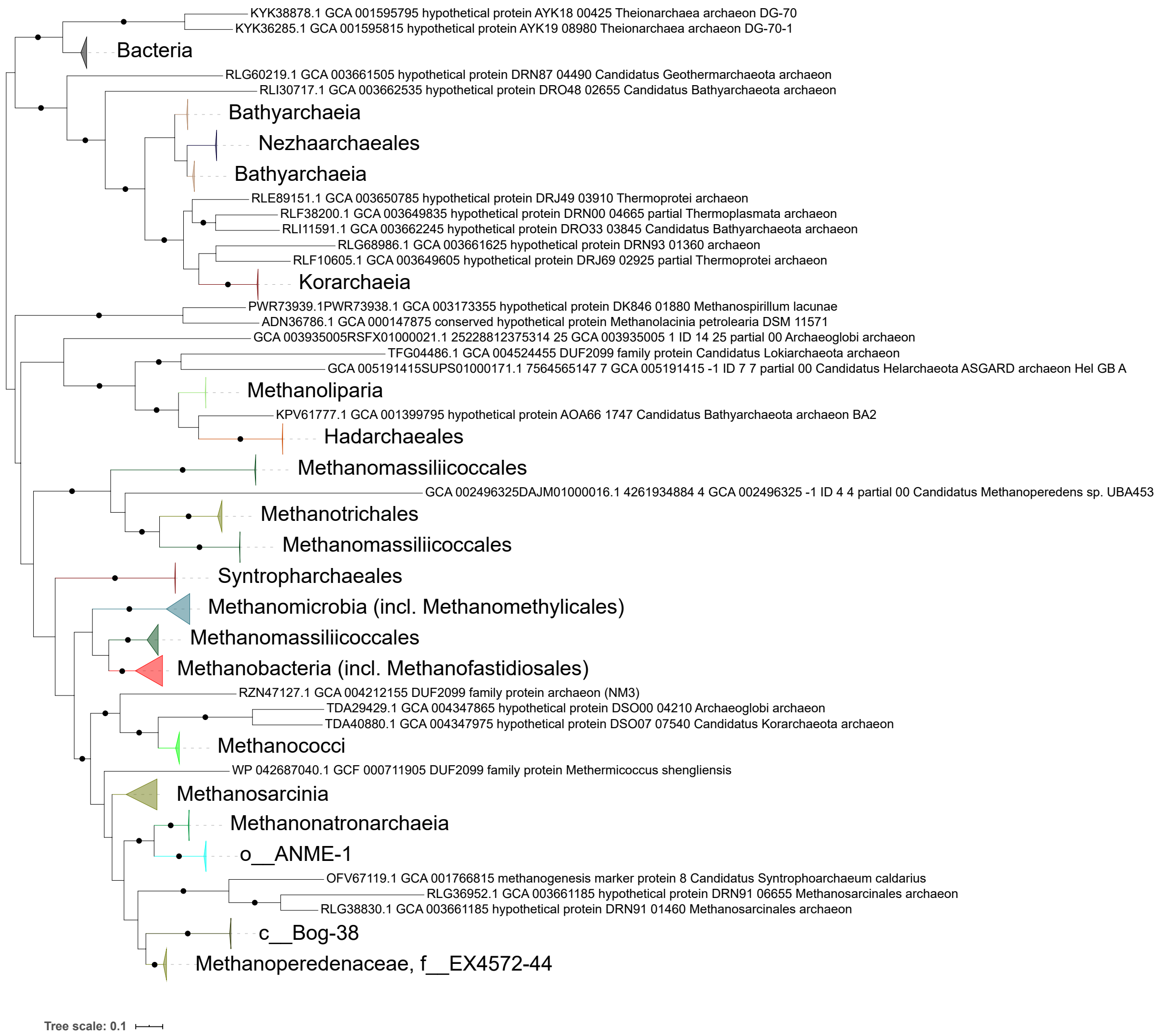

Fig. S34.
ML phylogeny of methanogenesis marker m18. Black circles indicate strongly supported branches (ultrafast bootstrap >=95, aLRT SH-like >=80).

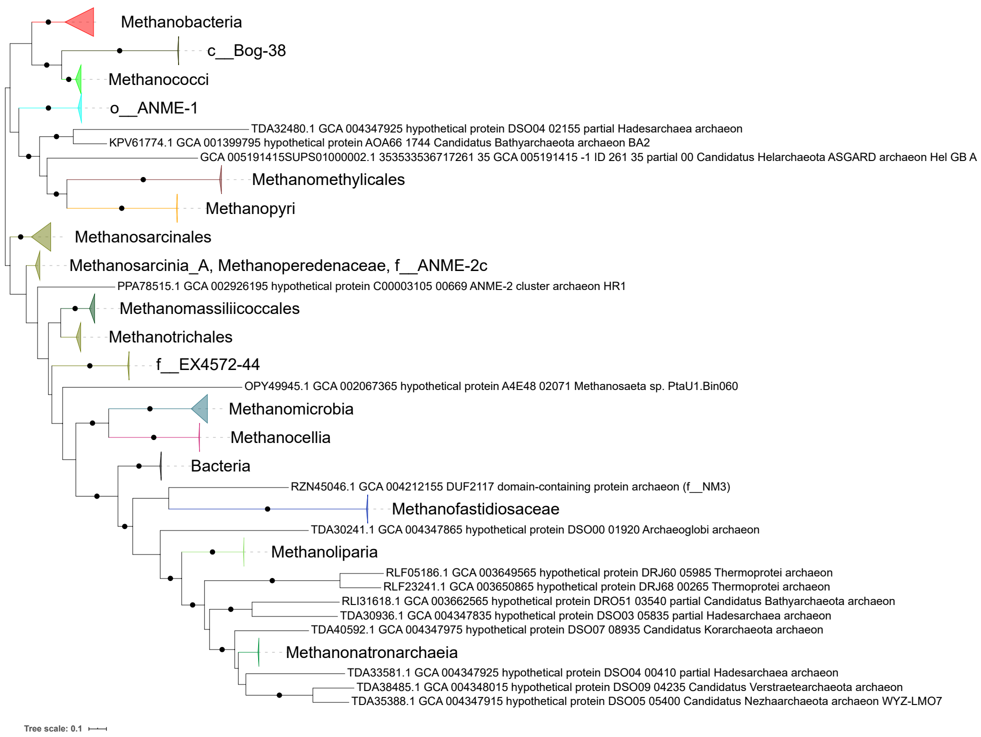

Fig. S35.
ML phylogeny of methanogenesis marker m19. Black circles indicate strongly supported branches (ultrafast bootstrap >=95, aLRT SH-like >=80).

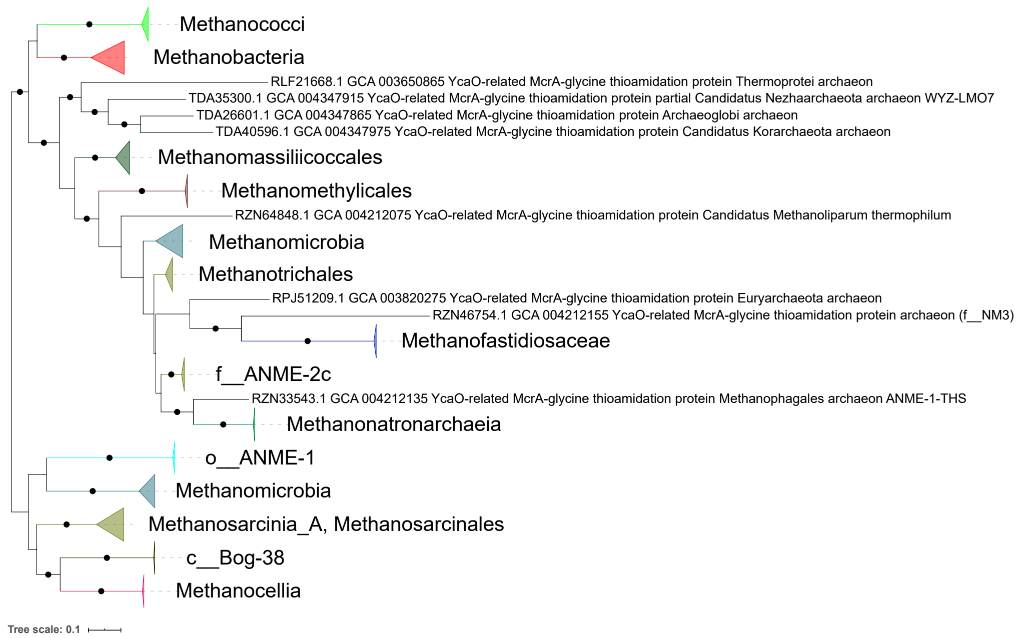

Fig. S36.
ML phylogeny of methanogenesis marker m20. Black circles indicate strongly supported branches (ultrafast bootstrap >=95, aLRT SH-like >=80).

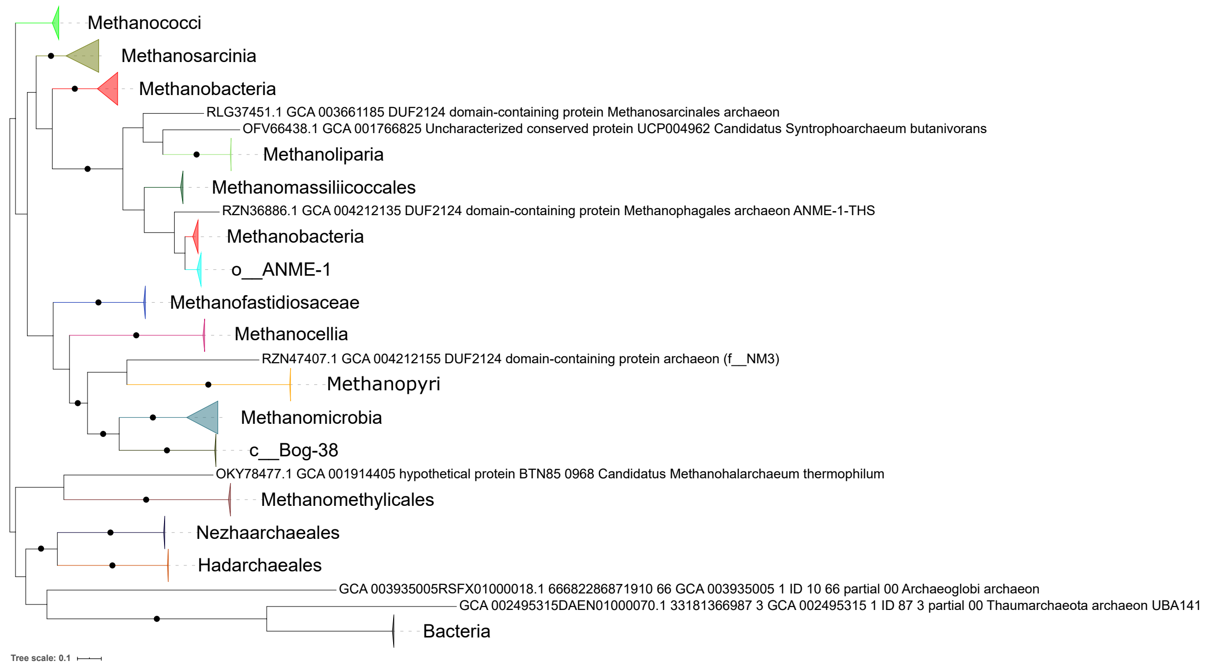

Fig. S37.
ML phylogeny of methanogenesis marker m21. Black circles indicate strongly supported branches (ultrafast bootstrap >=95, aLRT SH-like >=80).

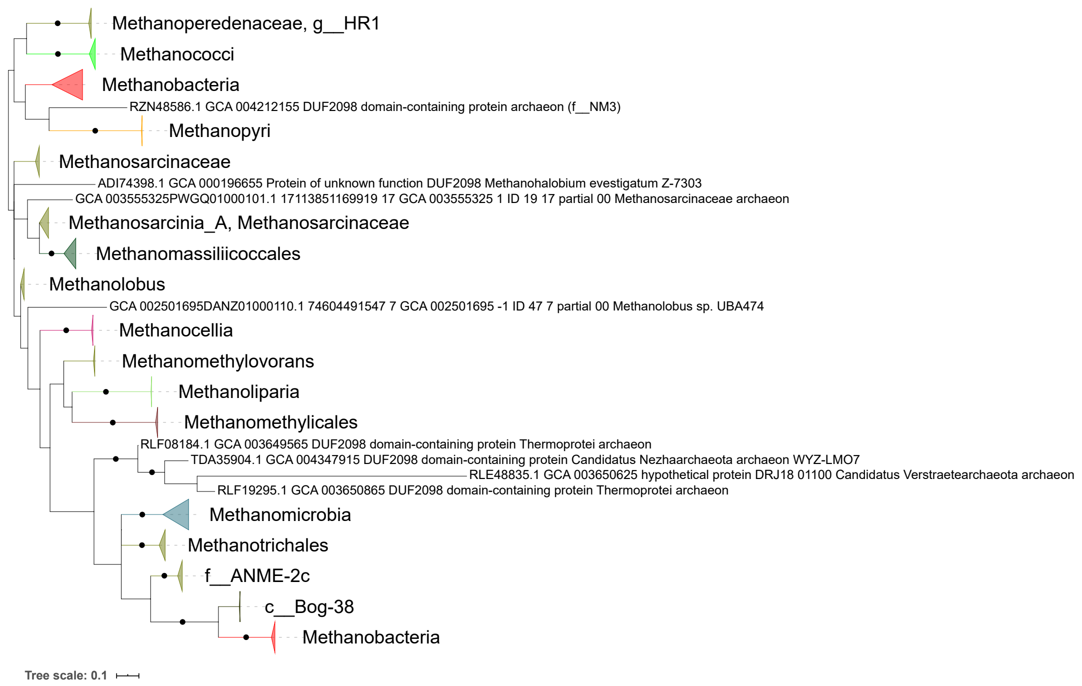

Fig. S38.
ML phylogeny of methanogenesis marker m22. Black circles indicate strongly supported branches (ultrafast bootstrap >=95, aLRT SH-like >=80).

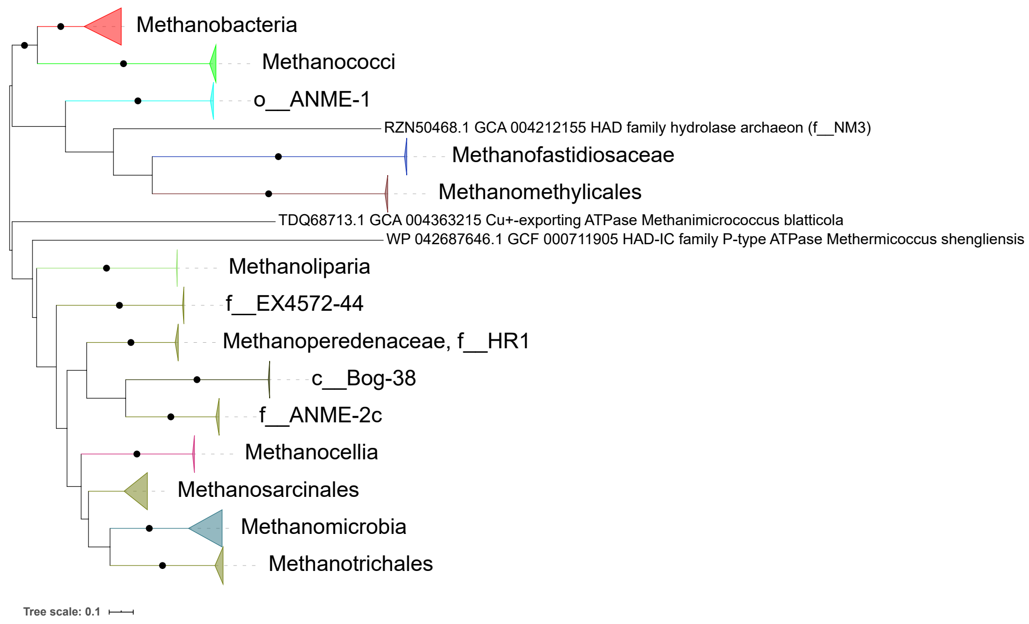

Fig. S39.
ML phylogeny of methanogenesis marker m23. Black circles indicate strongly supported branches (ultrafast bootstrap >=95, aLRT SH-like >=80).

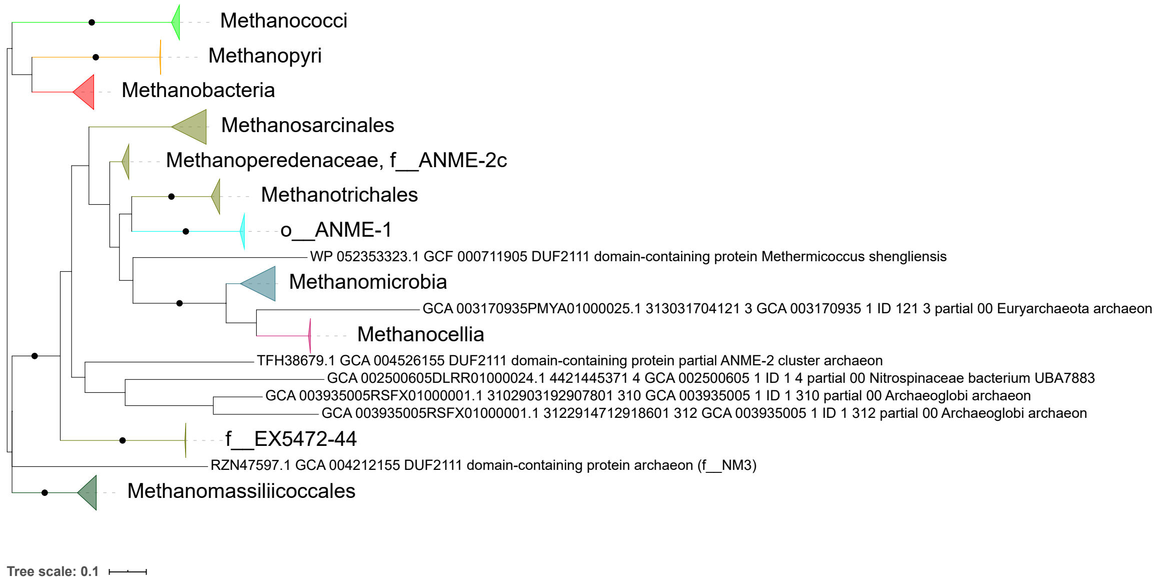

Fig. S40.
ML phylogeny of methanogenesis marker m24. Black circles indicate strongly supported branches (ultrafast bootstrap >=95, aLRT SH-like >=80).

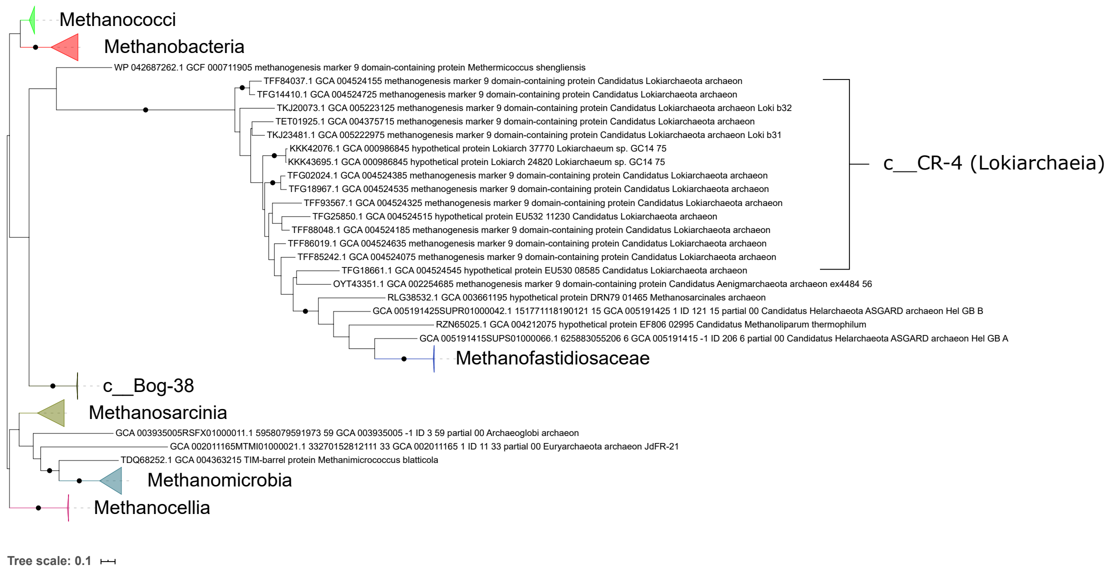

Fig. S41.
ML phylogeny of methanogenesis marker m25. Black circles indicate strongly supported branches (ultrafast bootstrap >=95, aLRT SH-like >=80).

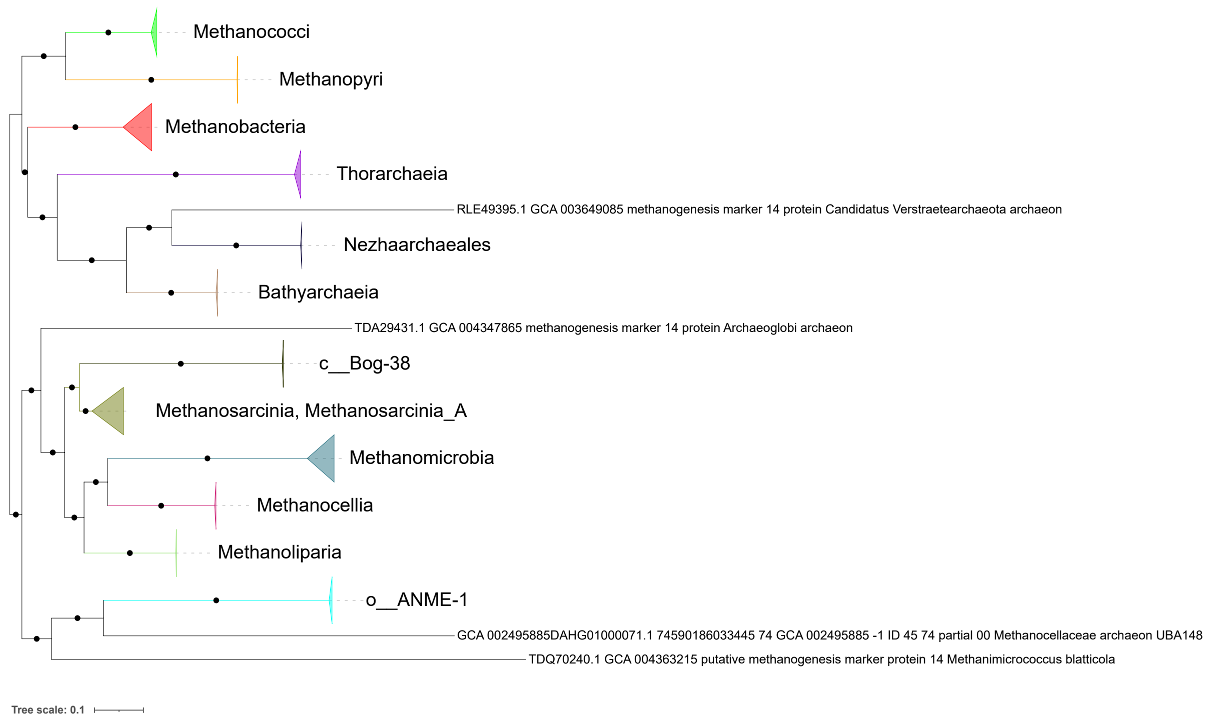

Fig. S42.
ML phylogeny of methanogenesis marker m26. Black circles indicate strongly supported branches (ultrafast bootstrap >=95, aLRT SH-like >=80).

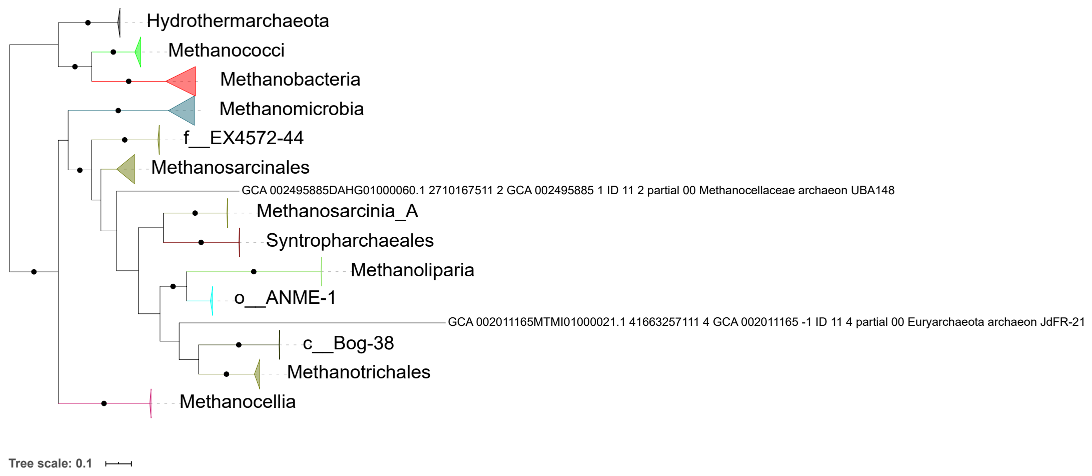

Fig. S43.
ML phylogeny of methanogenesis marker m32. Black circles indicate strongly supported branches (ultrafast bootstrap >=95, aLRT SH-like >=80).

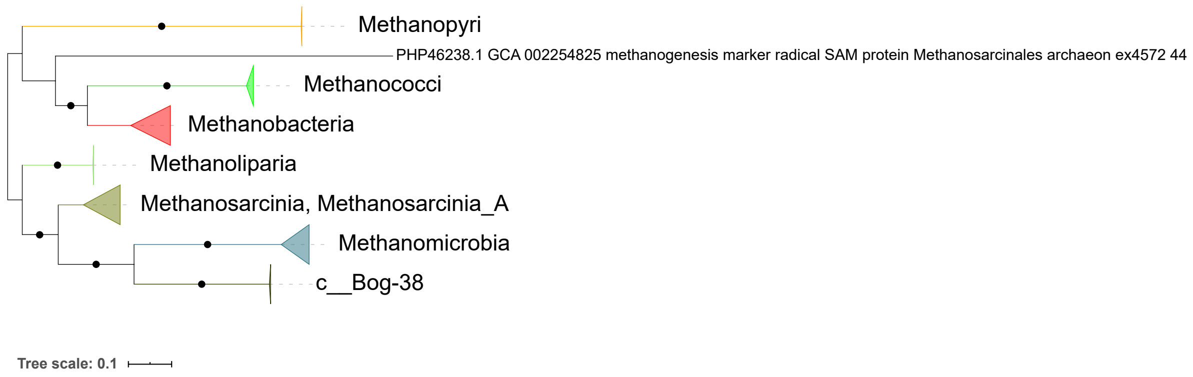

Fig. S44.
ML phylogeny of methanogenesis marker m33. Black circles indicate strongly supported branches (ultrafast bootstrap >=95, aLRT SH-like >=80).

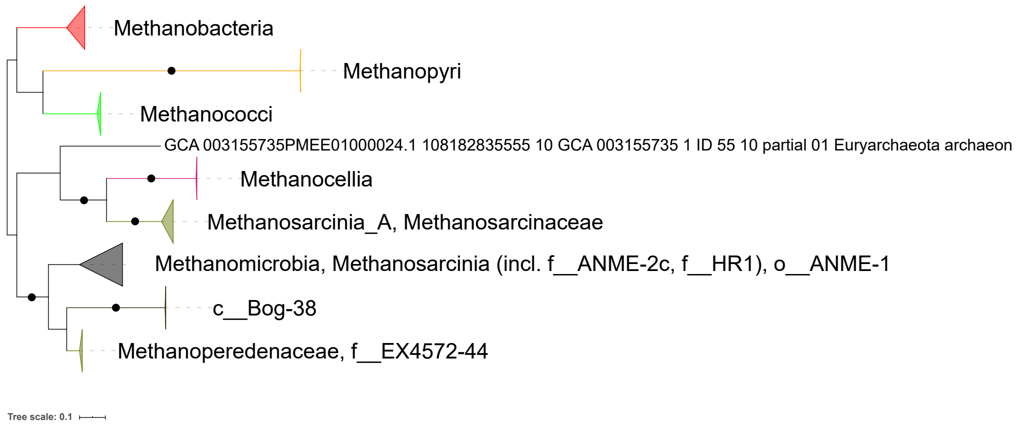

Fig. S45.
ML phylogeny of methanogenesis marker m34. Black circles indicate strongly supported branches (ultrafast bootstrap >=95, aLRT SH-like >=80).

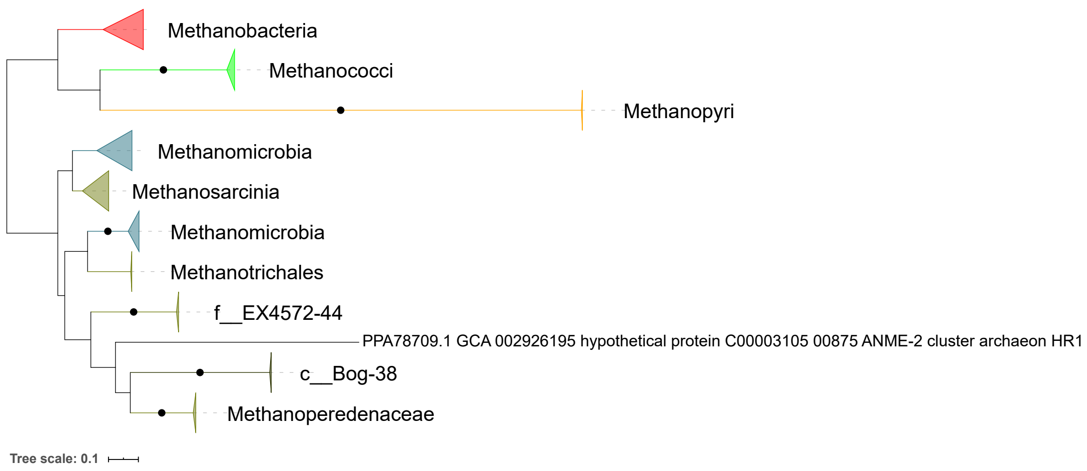

Fig. S46.
ML phylogeny of methanogenesis marker m35. Black circles indicate strongly supported branches (ultrafast bootstrap >=95, aLRT SH-like >=80).

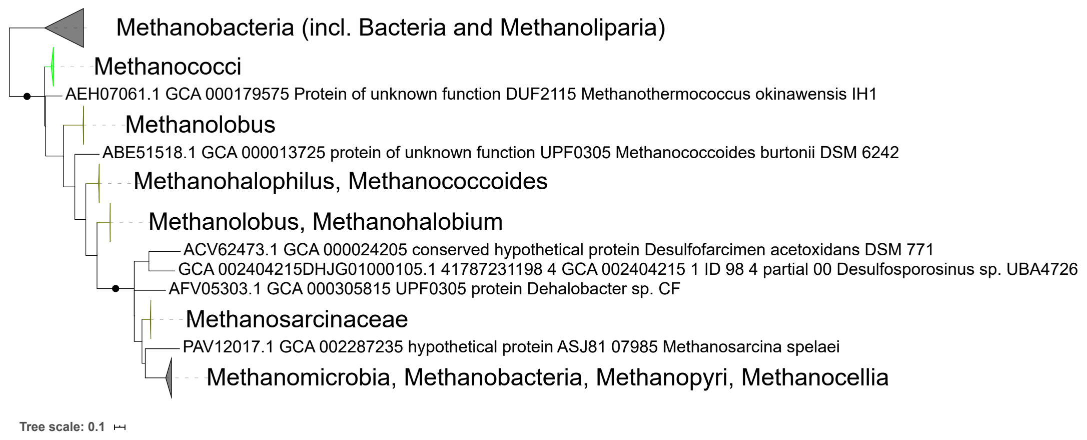

Fig. S47.
ML phylogeny of methanogenesis marker m36. Black circles indicate strongly supported branches (ultrafast bootstrap >=95, aLRT SH-like >=80).

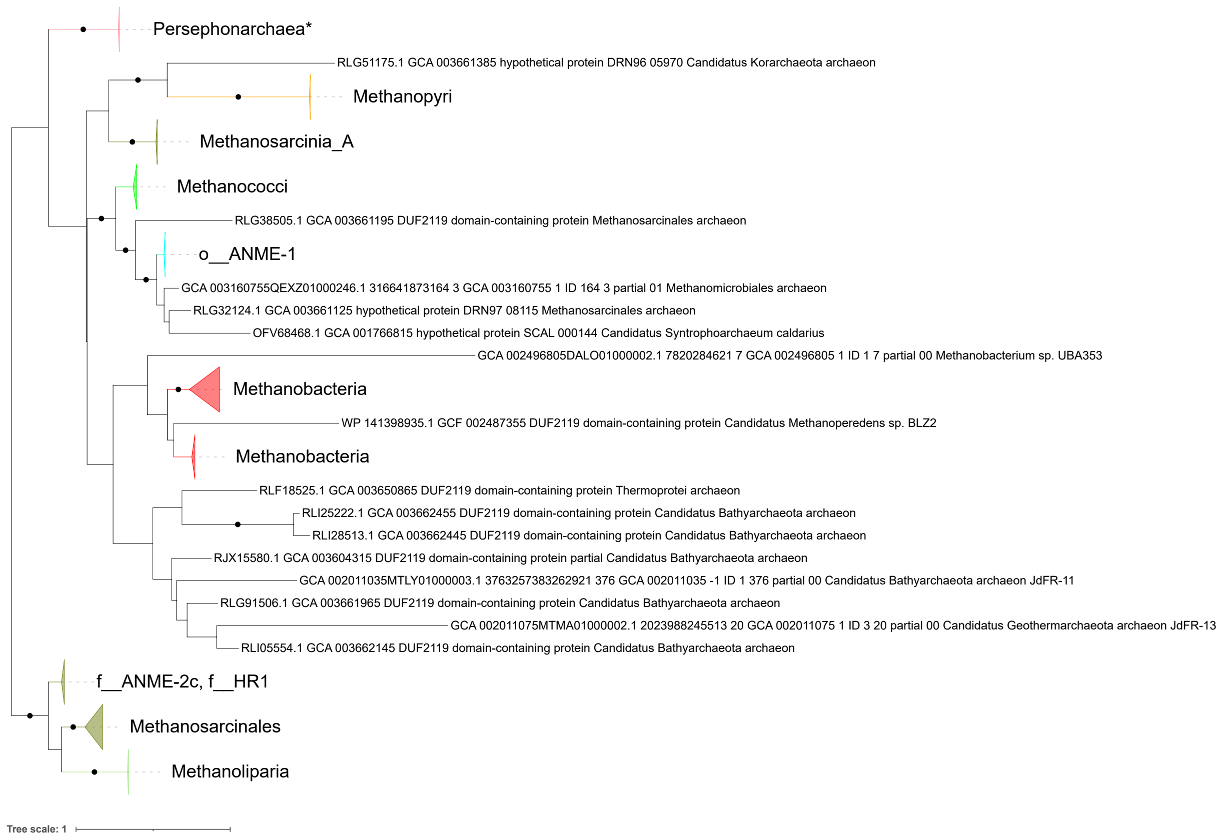

Fig. S48.
ML phylogeny of methanogenesis marker m37. Black circles indicate strongly supported branches (ultrafast bootstrap >=95, aLRT SH-like >=80).

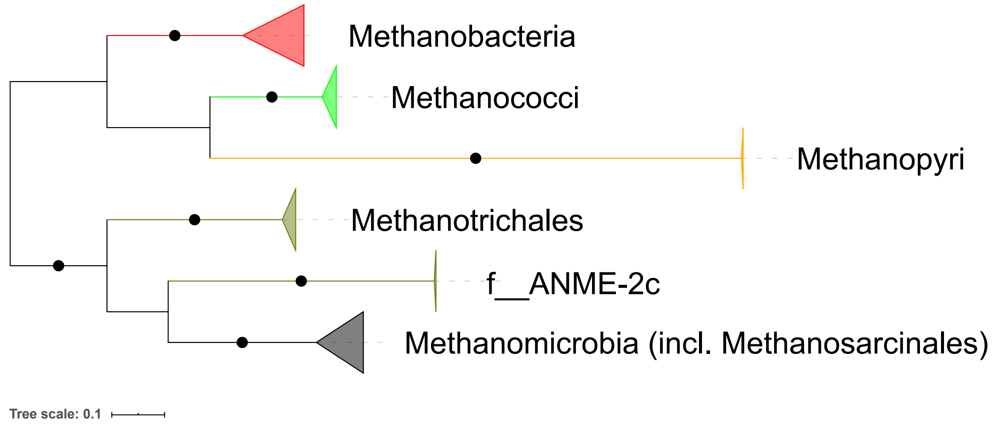

Fig. S49.
ML phylogeny of methanogenesis marker m38. Black circles indicate strongly supported branches (ultrafast bootstrap >=95, aLRT SH-like >=80).

A.

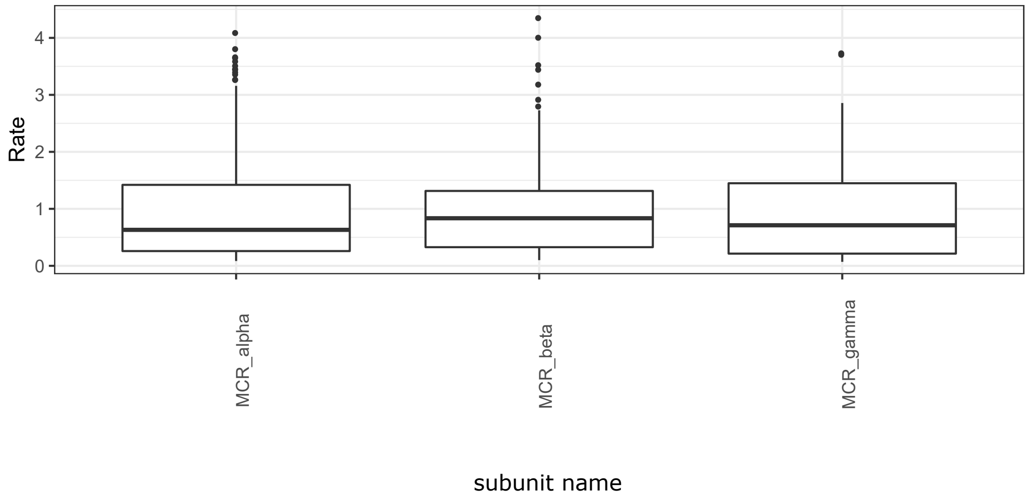

Rate
0
1
2
3
4
MCR_alpha
MCR_beta
MCR_gamma
subunit name

B.

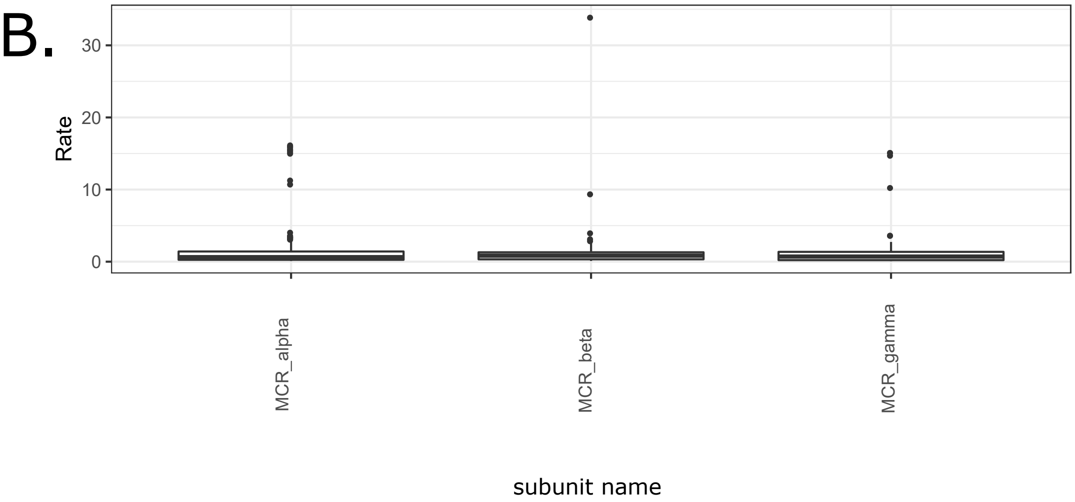

Rate
0
10
20
30
MCR_alpha
MCR_beta
MCR_gamma
subunit name

C.

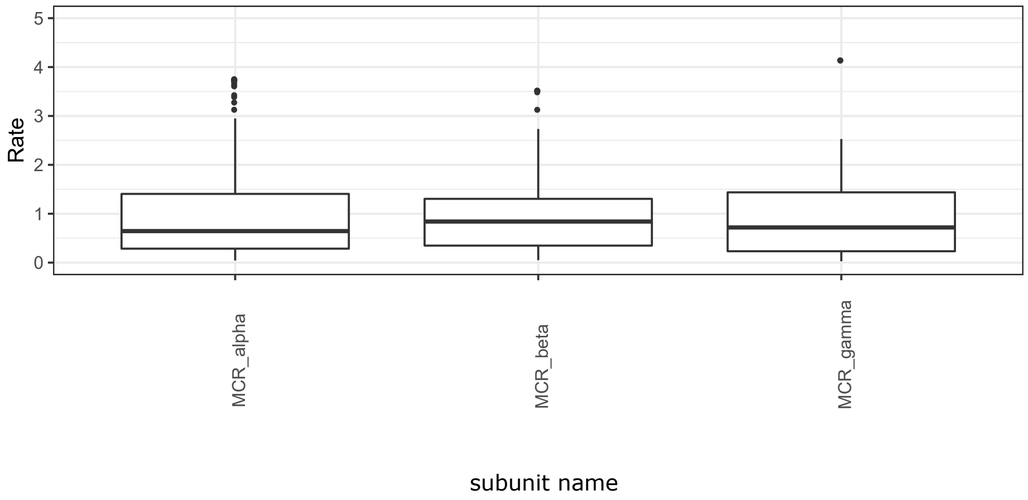

Rate
0
1
2
3
4
5
MCR_alpha
MCR_beta
MCR_gamma
subunit name

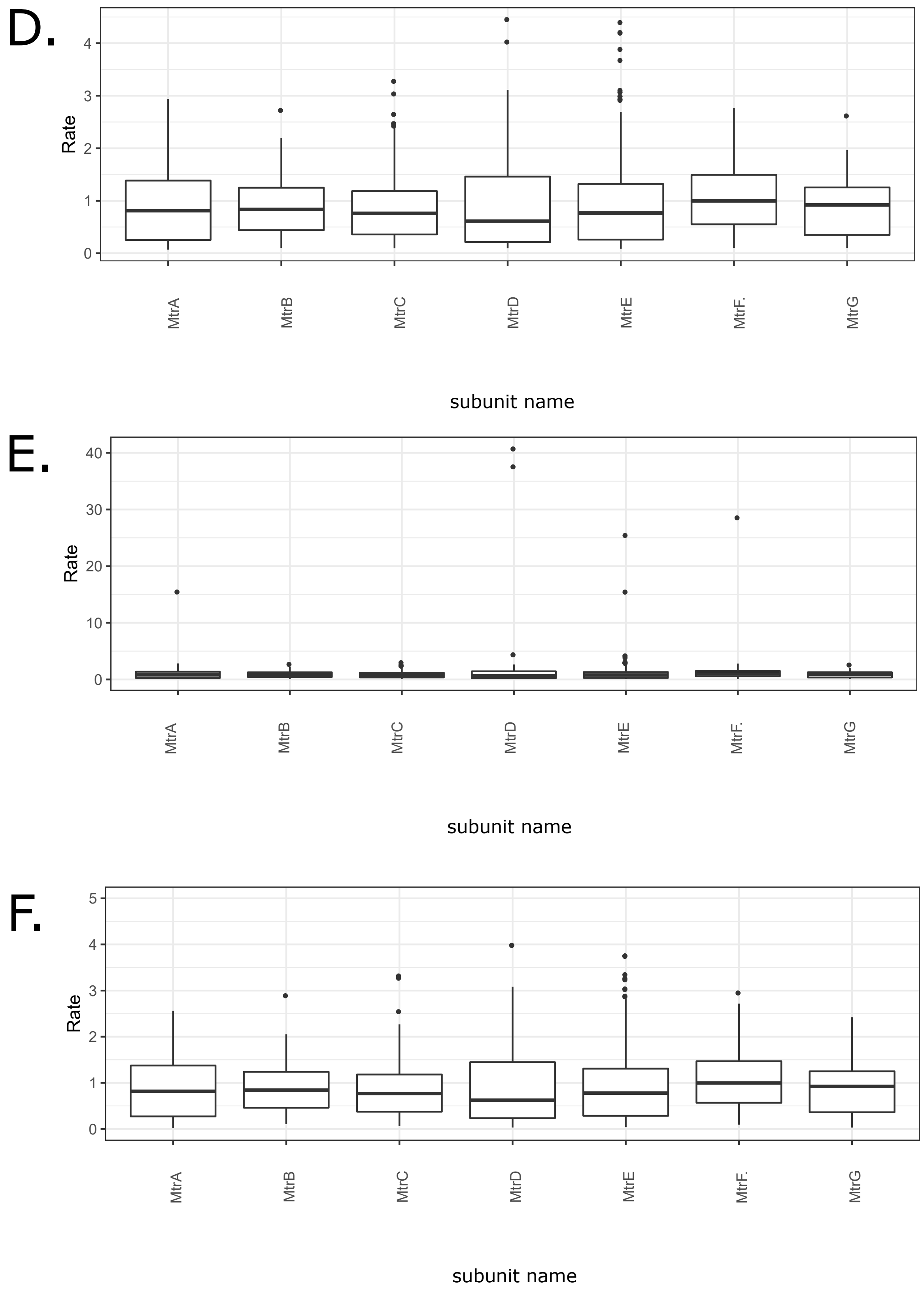
D.
Rate
0
1
2
3
4
MtrA
MtrB
MtrC
MtrD
MtrE
MtrF.
MtrG
subunit name
E.
Rate
0
10
20
30
40
MtrA
MtrB
MtrC
MtrD
MtrE
MtrF.
MtrG
subunit name
F.
Rate
0
1
2
3
4
5
MtrA
MtrB
MtrC
MtrD
MtrE
MtrF.
MtrG
subunit name

G.

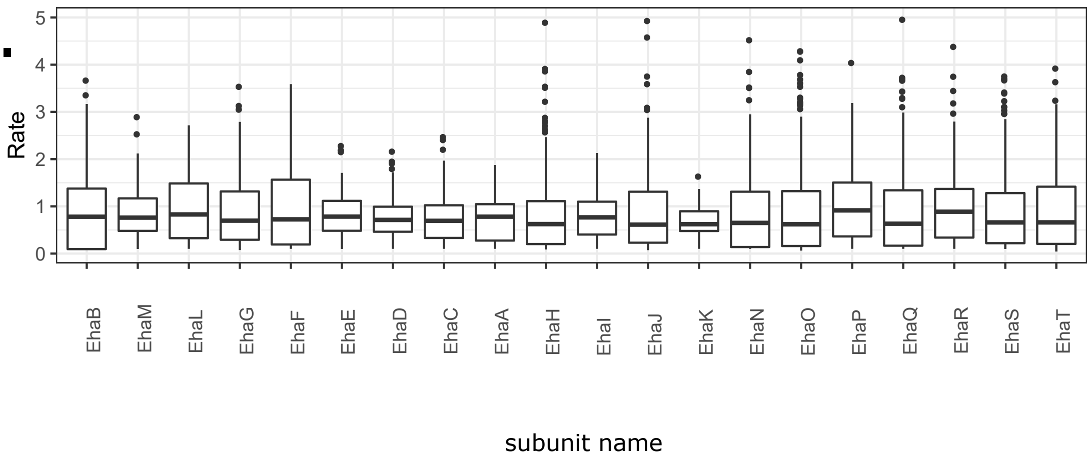
5
4
3
2
1
0
Rate
EhaB
EhaM
EhaL
EhaG
EhaF
EhaE
EhaD
EhaC
EhaA
EhaH
EhaI
EhaJ
EhaK
EhaN
EhaO
EhaP
EhaQ
EhaR
EhaS
EhaT
subunit name

H.

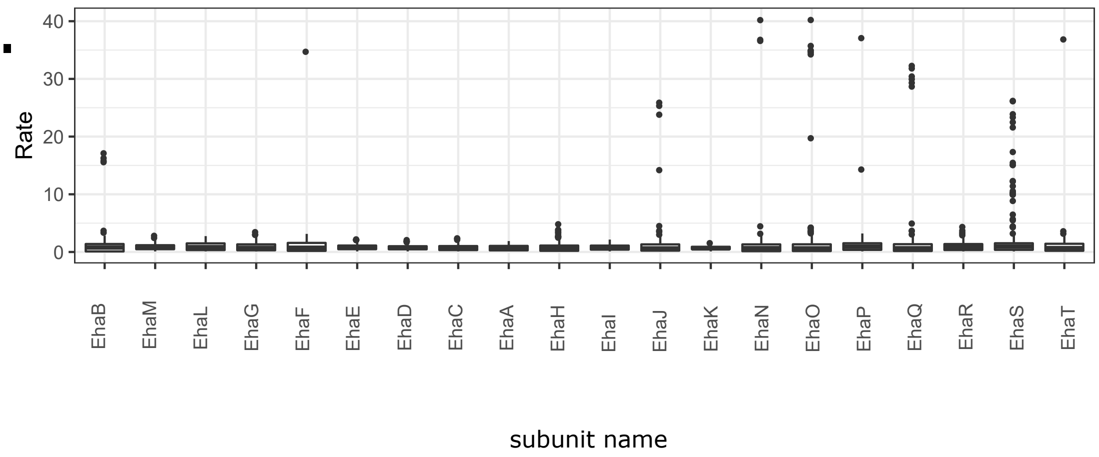
40
30
20
10
0
Rate
EhaB
EhaM
EhaL
EhaG
EhaF
EhaE
EhaD
EhaC
EhaA
EhaH
EhaI
EhaJ
EhaK
EhaN
EhaO
EhaP
EhaQ
EhaR
EhaS
EhaT
subunit name

I.

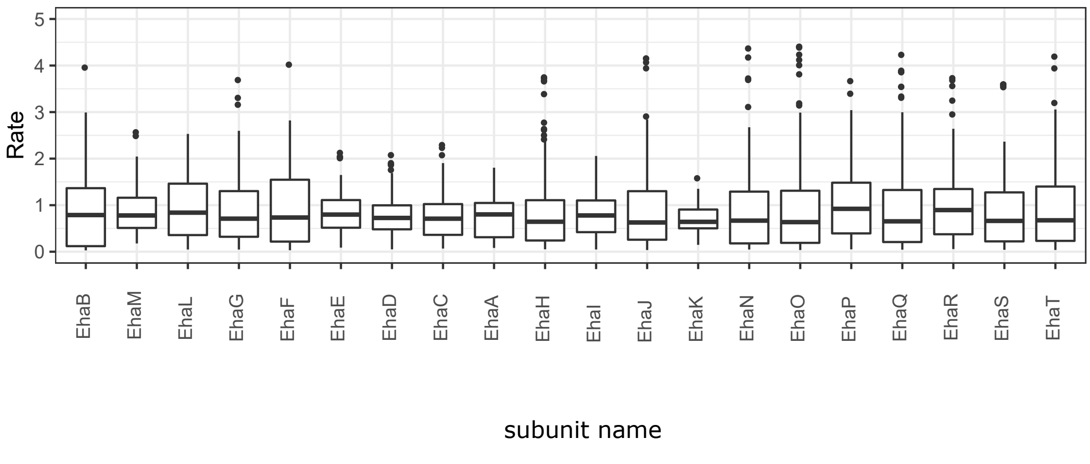
5
4
3
2
1
0
Rate
EhaB
EhaM
EhaL
EhaG
EhaF
EhaE
EhaD
EhaC
EhaA
EhaH
EhaI
EhaJ
EhaK
EhaN
EhaO
EhaP
EhaQ
EhaR
EhaS
EhaT
subunit name

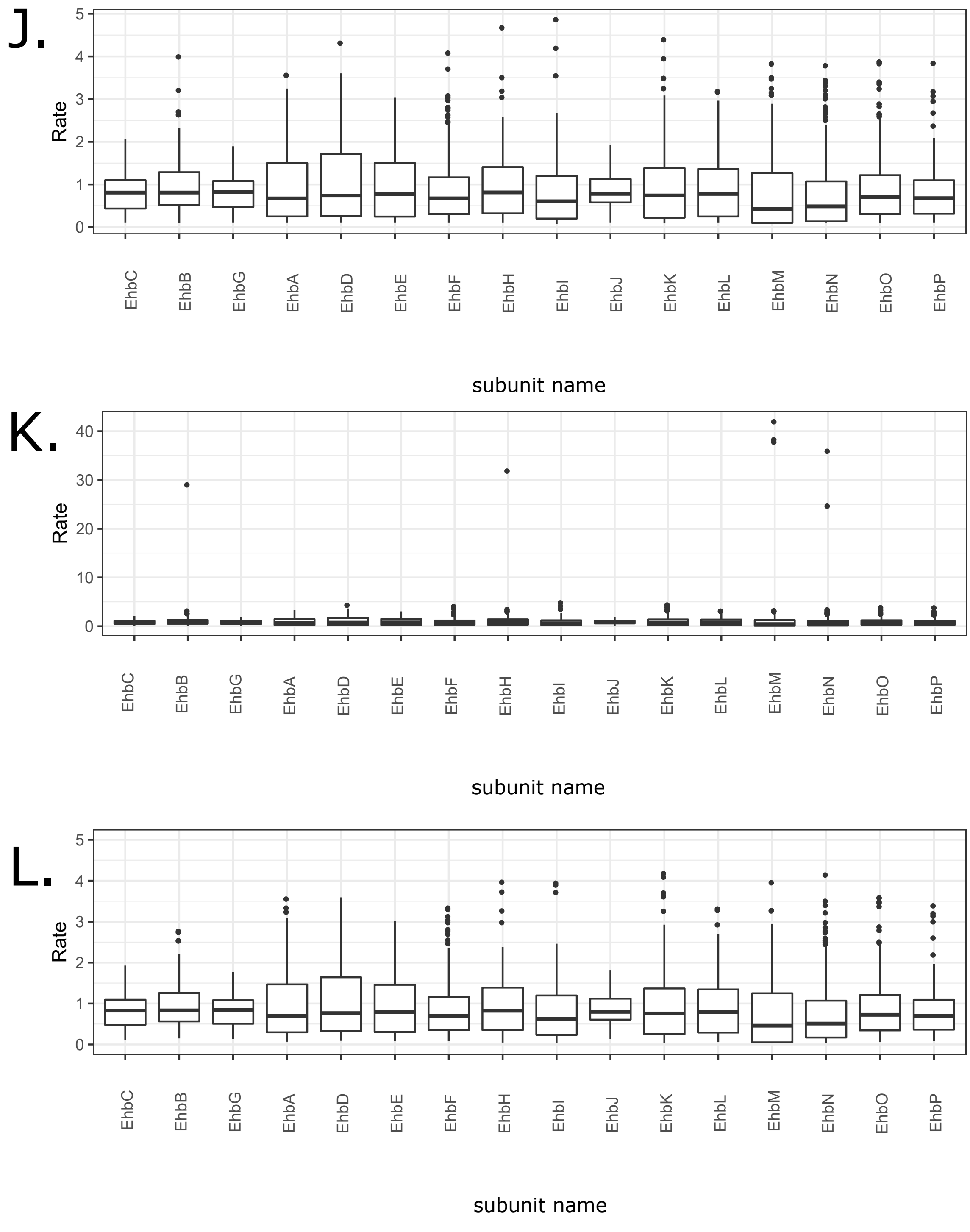

Fig. S50.

Boxplots for site-specific evolutionary rates for each subunit of (**A**, **B**, **C**) Mcr, (**D**, **E**, **F**) Mtr, (**G**, **H**, **I**) Eha, (**J**, **K**, **L**) Ehb. The plots for each complex correspond to Poisson ML, Poisson+G16 ML, Poisson+G16 empirical Bayesian respectively.

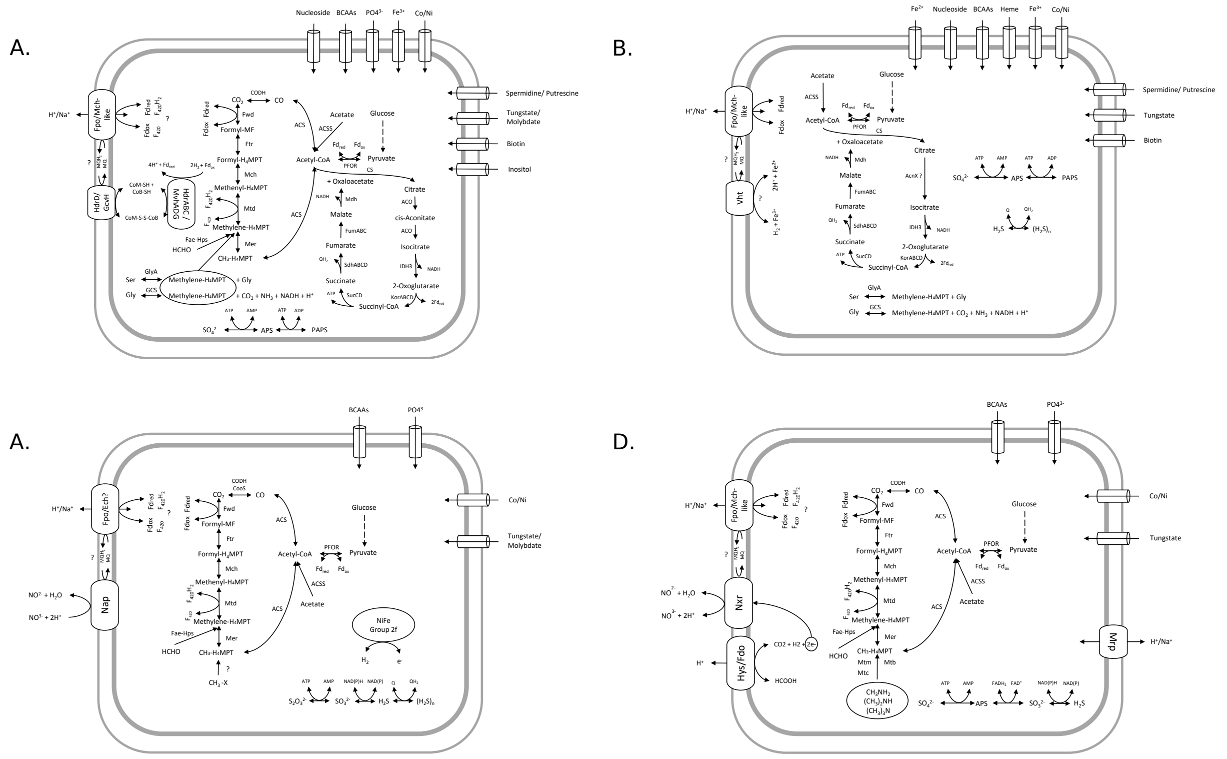

Fig. S51.

Metabolic reconstructions for (**A**) *Ca.* Scotarchaeum ottlingeri*, (**B**) *Ca.* Geothermarchaeum rappei*, (**C**) *Ca.* Pyrohabitans jungbluthii* and (**D**) *Ca.* Hydrothermarchaeum profundi*.

A.

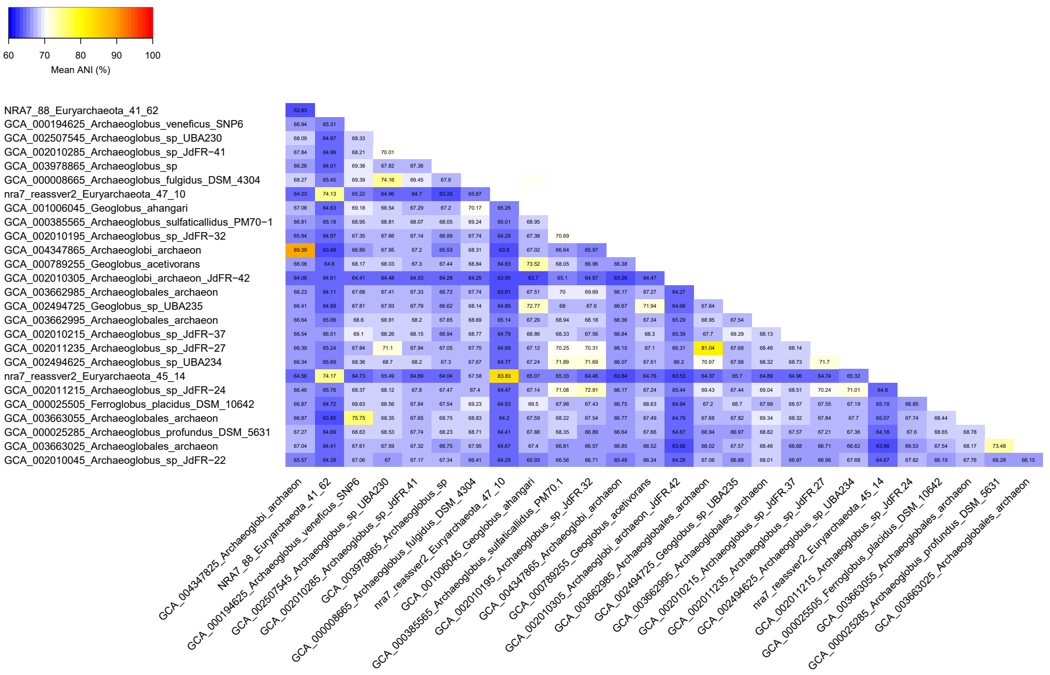

B.

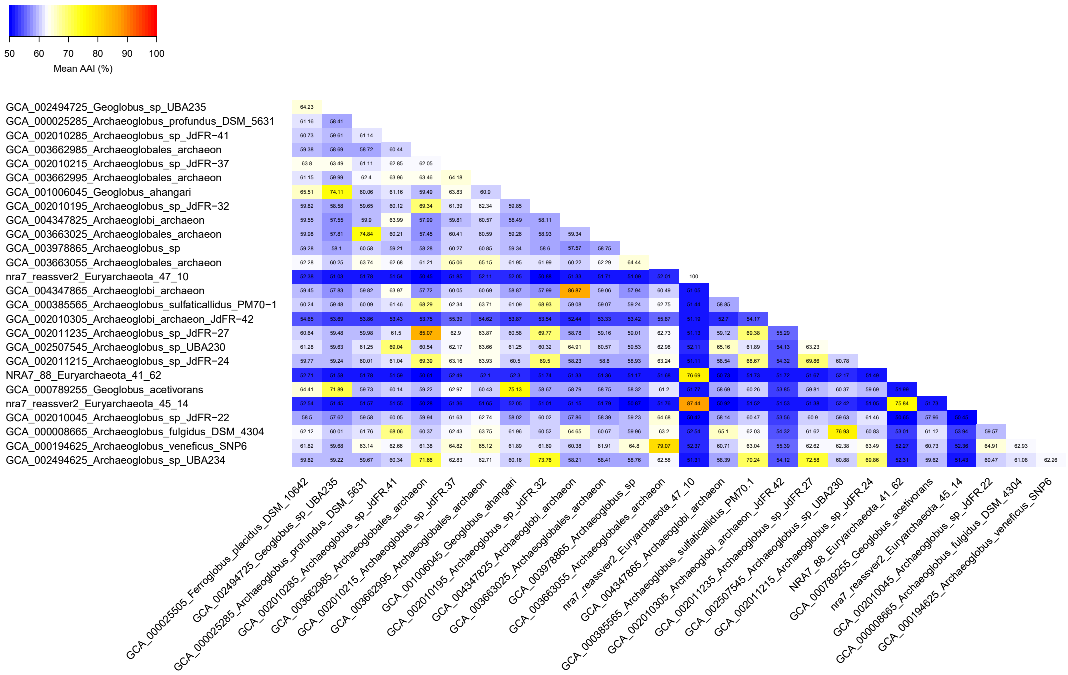

Fig. S52.
Heatmap graphs of (**A**) mean pairwise ANI (%), (**B**) mean pairwise AAI (%) of Archaeoglobi GTDB representative genomes with the Mnemosynellales* from this study (*Ca.* M. biddleae* substituting s__JdFR-21 sp002011165 and *Ca.* M. bozhongmuii* substituting s__JdFR-21 sp014361165).

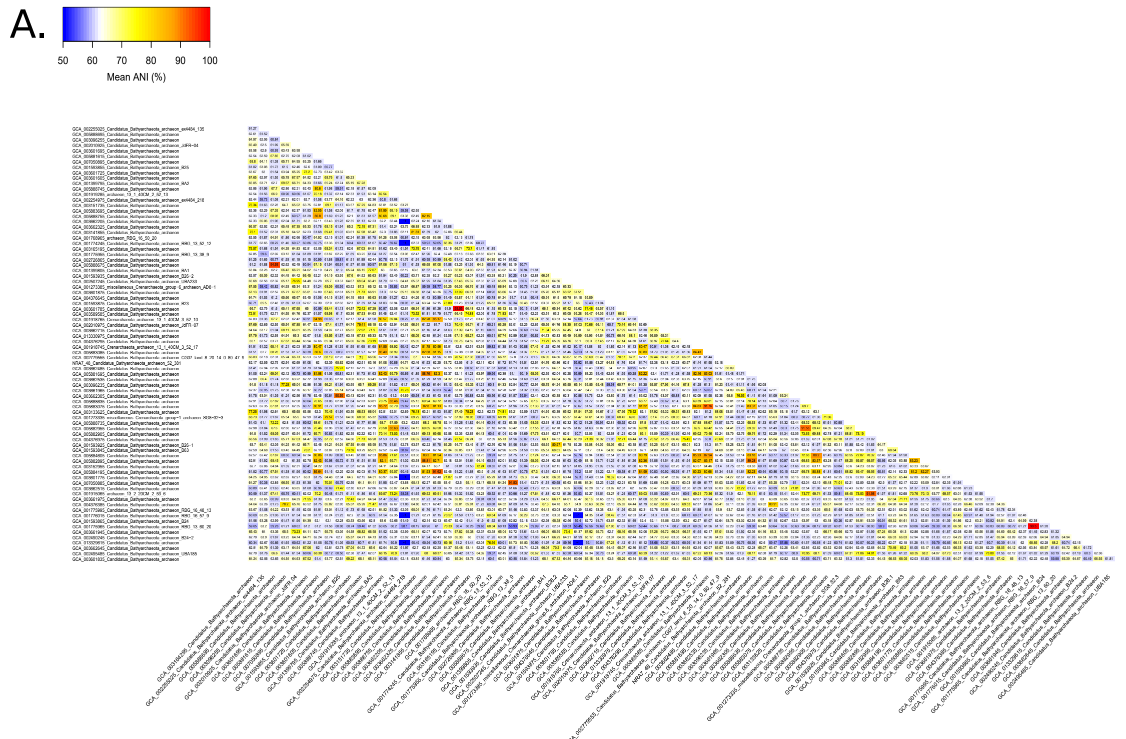

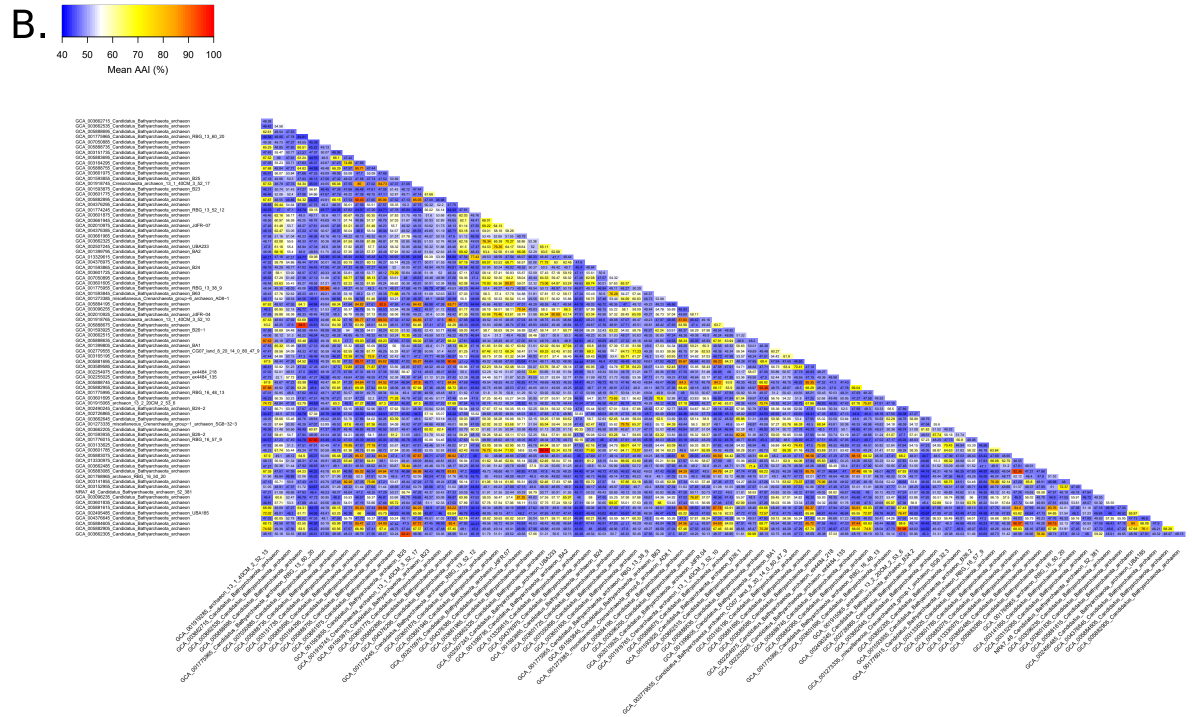

Fig. S53.
Heatmap graphs of (**A**) mean pairwise ANI (%), and (**B**) mean pairwise AAI (%) of Bathyarchaeia GTDB representative genomes with *Ca.* H. orcuttiae* substituting s__JdFR-11 sp002011035.

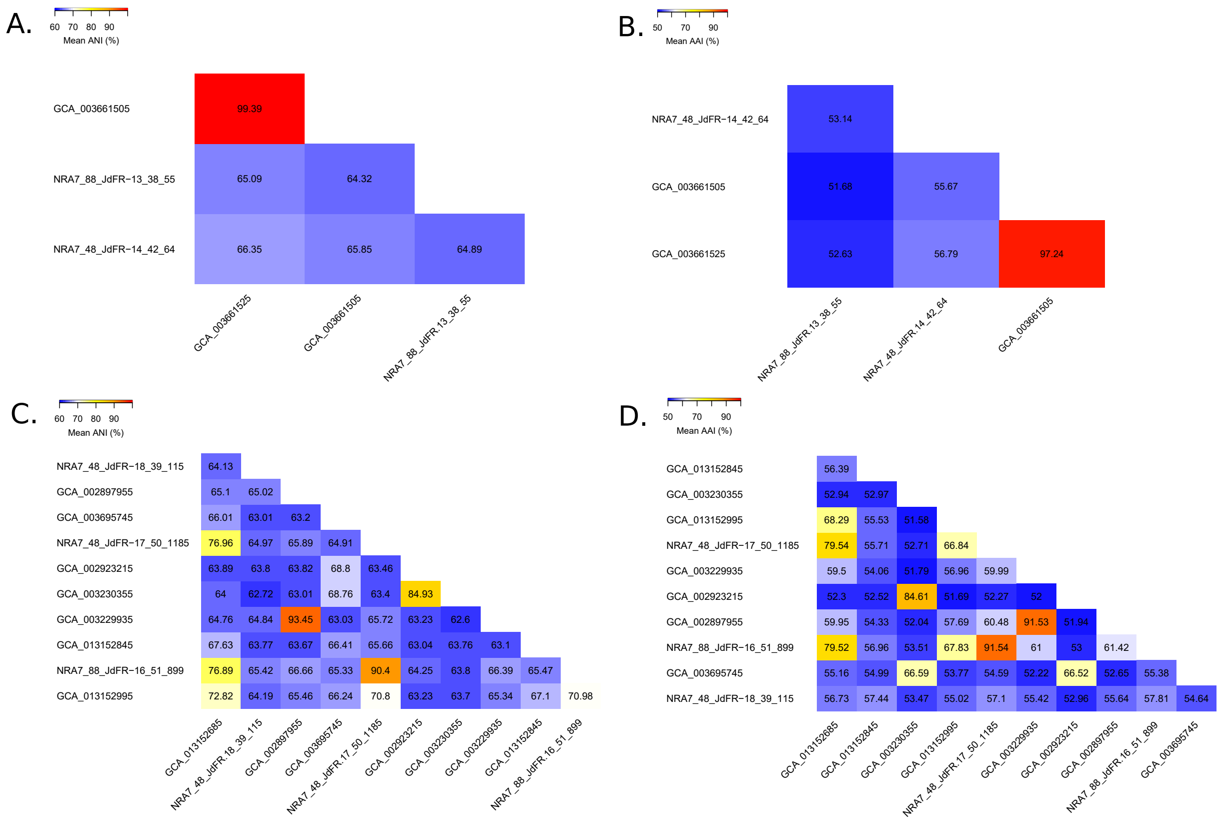

Fig. S54.

Heatmap graphs of (**A**) mean pairwise ANI (%), (**B**) mean pairwise AAI (%) of Geothermarchaeales GTDB representative genomes with *Ca.* Scotarchaeum otlingeri* substituting s__JdFR-13 sp002011075 and *Ca*. Geothermarchaeum rappei* substituting s__JDFR-14 sp002011085, (**C**) mean pairwise ANI (%), and (**D**) mean pairwise AAI (%) of Hydrothermarchaeota GTDB representative genomes with *Ca*. Pyrohabitans jungbluthii* (JdFR-16) and *Ca*. pyrohabitans sp.* (JdFR-17) added.

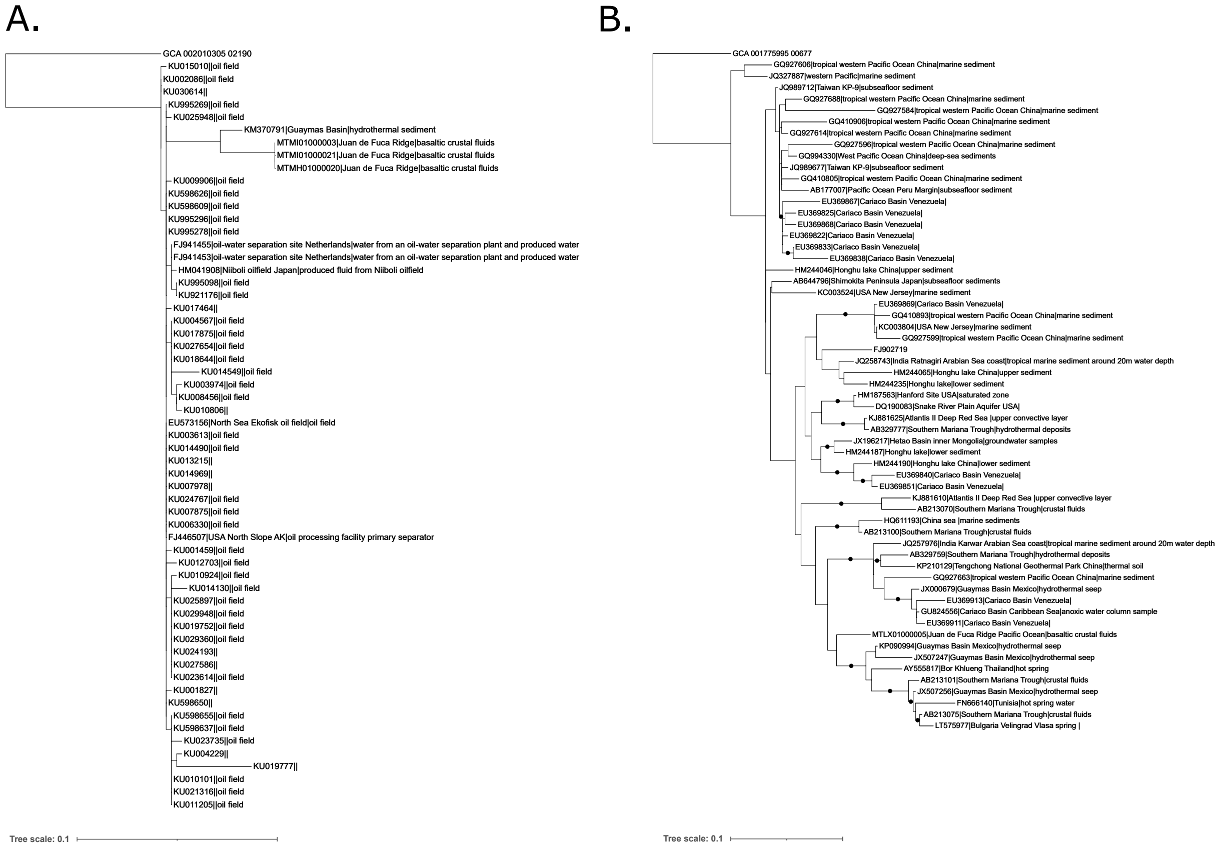

Fig. S55.

ML phylogenies of the 16S genes in (**A**) Mnemosynellales and (**B**) Hecatellales. Each leaf contains the sequence accession, pipe separated with its sampling locality, and environment, depending on their availability in each sequence's metadata in SILVA. Black circles indicate strongly supported branches (ultrafast bootstrap >=95, aLRT SH-like >=80).

Borrel et al., 2019	EggNOG accession number	Gao & Gupta, 2007
m1_mcrA	arCOG04857	MMP1559
m2_mcrB	arCOG04860	MMP1555
m3_mcrG	arCOG04858	MMP1558
m4_Predicted rotamase	arCOG04900	MMP0154
m5_DUF2102	arCOG04901	MMP0337
m6_DUF2112	arCOG04903	MMP0312
m7_YjiL-like	arCOG02679	MMP0608
m8_DUF2113	arCOG04904	MMP0656
m9_arCOG03226	arCOG03226	MMP0421
m10_atwA	arCOG00185	MMP0620
m11_mcrC	arCOG03225	MMP1557
m12_mcrD	arCOG04859	MMP1556
m13_cfbD	arCOG04888	MMP0428
m14_cfbE	arCOG02822	MMP0173
m15_mtxX	arCOG00854	MMP1346
m16_AIR-synthase-related	arCOG00640	MMP1531
m17_Zn-ribbon protein	arCOG01116	MMP1593
m18_DUF2099	arCOG04893	MMP1644
m19_DUF2117	arCOG03231	MMP0143
m20_Gly-thioamidase	arCOG02882	MMP1056
m21_DUF2124	arCOG04847	MMP0563
m22_DUF2098	arCOG04846	MMP0001
m23_SolubleP-type ATPase	arCOG01579	MMP1641
m24_DUF2111	arCOG04902	MMP0311
m25_arCOG04853	arCOG04853	MMP1704
m26_DUF2114	arCOG04866	MMP1223
m27_mtrA	arCOG03221	MMP1564
m28_mtrB	arCOG04867	MMP1563
m29_mtrC	arCOG04868	MMP1562
m30_mtrD	arCOG04869	MMP1561
m31_mtrE	arCOG04870	MMP1560
m32_arCOG04885	arCOG04885	MMP0642
m33_Arg-methyltransferase	arCOG00950	MMP1554
m34_DUF1894	arCOG04844	MMP0698
m35_DUF1890	arCOG04845	MMP0701
m36_DUF2115	arCOG03215	MMP0665
m37_DUF2119	arCOG04894	MMP1309
m38_DUF2121	arCOG03213	MMP0021

Table S1.

Table of all 38 (m1-m38) methanogenesis marker genes following the set and numbering in Borrel et al.(*11*), their respective EggNOG accession numbers and *Methanococcus maripaludis* gene names as mentioned in (*27*). Markers noted in red are not included in Gao & Gupta(*27*).

Genome	Proposed Name	NCBI assembly	Genome Size (Mb)	Completeness (%)	Contamination	GC (%)
NRA7_88_Euryarchaeota_41_62	*Ca.* Mnemosynella biddleae	GCA_002011165	1.50	96.08	0.65	41.18
NRA7_48_Euryarchaeota_41_21		GCA_002011155	1.47	96.08	0.65	41.14
nra7_reassver2_Euryarchaeota_45_14	*Ca.* Mnemosynella bozhongmuii	GCA_014361165	1.29	93.46	1.31	44.82
nra7_reassver2_Euryarchaeota_47_10	*Ca.* Mnemosynella sp.	GCA_014361185	0.90	81.05	0.65	46.69
NRA7_48_Candidatus_Bathyarchaeota_archaeon_52_381	*Ca.* Hecatella orcuttiae	GCA_002011035	1.75	100	0.93	51.85
NRA7_88_Candidatus_Bathyarchaeota_52_49		GCA_002009985	1.74	100	0.93	51.93
NRA7_88_JdFR-13_38_55	*Ca.* Scotarchaeum ottlingeri	GCA_002011075	1.70	93.20	0.97	37.67
NRA7_48_JdFR-14_42_64	*Ca.* Geothermarchaeum rappei	GCA_002011085	1.58	94.17	0.97	41.83
NRA7_88_JdFR-14_42_10		GCA_002011085	1.44	93.20	1.29	42.03
NRA7_88_JdFR-16_51_899	*Ca.* Pyrohabitans jungbluthii	GCA_002010065	1.99	98.13	2.80	51.20
NRA7_48_JdFR-16_51_665		GCA_002010065	1.17	51.04	1.87	51.06
NRA7_48_JdFR-17_50_1185		GCA_002011115	1.62	74.22	2.88	50.46
NRA7_48_JdFR-18_39_115		GCA_002011125	2.19	98.13	1.87	39.11

	Scaffolds (number)	Longest Scaffold (bp)	N50 (scaffolds)	Contigs (number)	Longest contig (bp)	N50 (contigs)	Coding density (%)	Predicted genes (number)
NRA7_88_Euryarchaeota_41_62	11	341116	235752	11	341116	235752	91.98	1730
NRA7_48_Euryarchaeota_41_21	21	238144	187849	21	238144	187849	92.84	1711
nra7_reassver2_Euryarchaeota_45_14	38	106407	54584	41	106407	44894	93.21	1505
nra7_reassver2_Euryarchaeota_47_10	73	68921	19803	88	68921	17146	95.50	1106
NRA7_48_Candidatus_Bathyarchaeota_archaeon_52_381	3	731052	535545	3	731052	535545	89.25	1881
NRA7_88_Candidatus_Bathyarchaeota_52_49	9	1267205	1267205	13	769127	497978	89.37	1890
NRA7_88_JdFR-13_38_55	7	1676113	1676113	11	691515	495125	90.26	1750
NRA7_48_JdFR-14_42_64	1	1583546	1583546	1	1583546	1583546	92.28	1613
NRA7_88_JdFR-14_42_10	204	69808	11815	243	40089	9032	92.17	1644
NRA7_88_JdFR-16_51_899	15	620739	394757	15	620739	394757	92.48	2246
NRA7_48_JdFR-16_51_665	82	64021	22921	82	64021	22921	92.30	1370
NRA7_48_JdFR-17_50_1185	101	89324	20372	124	89324	19456	91.32	1880
NRA7_48_JdFR-18_39_115	19	1917514	1917514	24	1201297	1201297	91.69	2424

	5S	16S	23S	full tRNA	partial tRNA	stop codons
NRA7_88_Euryarchaeota_41_62	1 yes	no	1 partial	4/20	14/20	1 / 3
NRA7_48_Euryarchaeota_41_21	1 yes	no	no	4/20	14/20	1 / 3
nra7_reassver2_Euryarchaeota_45_14	no	no	no	3/20	14/20	1 / 3
nra7_reassver2_Euryarchaeota_47_10	no	no	no	1/20	10/20	0 / 3
NRA7_48_Candidatus_Bathyarchaeota_archaeon_52_381	no	2 partial	2 yes	2/20	9/20	0 / 3
NRA7_88_Candidatus_Bathyarchaeota_52_49	no	2 partial	1 yes / 1 partial	2/20	9/20	0 / 3
NRA7_88_JdFR-13_38_55	1 yes	2 partial	1 yes	2/20	12/20	0 / 3
NRA7_48_JdFR-14_42_64	1 yes	1 partial	no	1/20	13/20	0 / 3
NRA7_88_JdFR-14_42_10	1 yes	no	no	1/20	12/20	0 / 3
NRA7_88_JdFR-16_51_899	1 yes	1 yes	1 yes	4/20	15/20	0 / 3
NRA7_48_JdFR-16_51_665	no	no	no	3/20	12/20	0 / 3
NRA7_48_JdFR-17_50_1185	1 yes	no	no	1/20	15/20	0 / 3
NRA7_48_JdFR-18_39_115	1 yes	1 partial	1 partial	4/20	16/20	0 / 3

Table S2.

Summary statistics of genomes binned in this study and their proposed names (see Descriptions of proposed taxa). Bins named NRA7_48 and NRA7_88 correspond to SRR3723048/U1362A and SRR3732688/U1362B from (*45*), while the “nra7_reassver2” bins originate from a co-assembly of the three Shengli metagenomes(*47*).

taxon	assembly	coordinates	location	isolation_source	metagenome_source
Euryarchaeota archaeon JdFR-21 *	GCA_002011165	47.76 N 127.76 W	Pacific Ocean	basaltic crustal fluids	subsurface metagenome
Archaeoglobi archaeon *	GCA_014361165	38.15 N 118.5 E	China: Dongying	Shengli oilfield at Shandong province	oil production facility metagenome
Archaeoglobi archaeon *	GCA_014361185	38.15 N 118.5 E	China: Dongying	Shengli oilfield at Shandong province	oil production facility metagenome
Candidatus Bathyarchaeota archaeon ex4484 218	GCA_002254975	27.0388 N 111.2456 W	USA: Guaymas Basin, Gulf of California	deep-sea hydrothermal vent sediments from dive 4484	marine sediment metagenome
Candidatus Bathyarchaeota archaeon	GCA_003661965	29.52416667 N 113.57000000 W	Mexico: Guaymas Basin, Gulf of California	deep-sea hydrothermal vent sediments from dive 4569_2 depth 12-15 cm	marine sediment metagenome
Candidatus Bathyarchaeota archaeon JdFR-11 *	GCA_002011035	47.76 N 127.76 W	Pacific Ocean	basaltic crustal fluids	subsurface metagenome
Candidatus Bathyarchaeota archaeon	GCA_003662515	29.52416667 N 113.57000000 W	Mexico: Guaymas Basin, Gulf of California	deep-sea hydrothermal vent sediments	marine sediment metagenome
Candidatus Bathyarchaeota archaeon	GCA_003662485	29.52416667 N 113.57000000 W	Mexico: Guaymas Basin, Gulf of California	deep-sea hydrothermal vent sediments from dive 4571_4 depth 12-15 cm	marine sediment metagenome
Candidatus Bathyarchaeota archaeon	GCA_003662305	29.52416667 N 113.57000000 W	Mexico: Guaymas Basin, Gulf of California	deep-sea hydrothermal vent sediments from dive 4569_2 depth 21-24 cm	marine sediment metagenome
Candidatus Bathyarchaeota archaeon B25	GCA_001593855	27.0119 N 111.4054 W	Mexico: Guaymas Basin, Gulf of California	marine sediment sample collected by push cores at a deep sea vent site during cruise AT15-25 on Alvin dive 4358	hydrothermal vent metagenome
Candidatus Thorarchaeota archaeon	GCA_004525055	44.0552806 N 28.6053 E	Romania: Tekirghiol	lake sediment	sediment metagenome
Candidatus Thorarchaeota archaeon	GCA_004524305	44.0552806 N 28.6053 E	Romania: Tekirghiol	lake sediment	sediment metagenome
Candidatus Thorarchaeota archaeon	GCA_003345595	41.3778 N 82.5108 W	USA: Ohio, Lake Erie, Old Woman Creek	freshwater wetland soil	wetland metagenome
Candidatus Thorarchaeota archaeon	GCA_003345555	41.3778 N 82.5108 W	USA: Ohio, Lake Erie, Old Woman Creek	freshwater wetland soil	wetland metagenome
Candidatus Thorarchaeota archaeon	GCA_004524445	44.0552806 N 28.6053 E	Romania: Tekirghiol	lake sediment	sediment metagenome
Candidatus Thorarchaeota archaeon	GCA_003662765	29.52416667 N 113.57000000 W	Mexico: Guaymas Basin, Gulf of California	deep-sea hydrothermal vent sediments from dive 4571_4 depth 0-3 cm	marine sediment metagenome
Candidatus Thorarchaeota archaeon SMTZ1-83	GCA_001563325	34.7414 N 77.1234 W	USA: White Oak River Estuary, North Carolina	Sulfate-methane transisition zone estuary sediments 16-26 cm	sediment metagenome
Candidatus Thorarchaeota archaeon	GCA_003662775	27.015 N 111.379 W	Mexico: Guaymas Basin, Gulf of California	deep-sea hydrothermal vent sediments from dive 4569_9 depth 0-3 cm	marine sediment metagenome
Candidatus Thorarchaeota archaeon	GCA_004376265	26.28 N 86.81 W	Atlantic Ocean	deep sea sediments associated with petroleum seepage	marine sediment metagenome
Candidatus Thorarchaeota archaeon AB_25	GCA_001940705	56.103333 N 10.45783299 E	Denmark: Aarhus Bay, Baltic Sea	Marine sediment	marine sediment metagenome
Candidatus Thorarchaeota archaeon MP11T_1	GCA_002825515	22.4992 N 114.0276 E	Hong Kong: Mai Po Nature Reserve	mangrove wetland sediments	sediment metagenome
Candidatus Thorarchaeota archaeon SMTZ1-45	GCA_001563335	34.7414 N 77.1234 W	USA: White Oak River Estuary, North Carolina	Sulfate-methane transisition zone estuary sediments 16-26 cm	sediment metagenome
Candidatus Thorarchaeota archaeon MP9T_1	GCA_002825535	22.4979 N 114.0295 E	Hong Kong: Mai Po Nature Reserve	sediment (20-25 cm) from mangrove covering field	sediment metagenome
Candidatus Thorarchaeota archaeon	GCA_003345545	41.3778 N 82.5108 W	USA: Ohio, Lake Erie, Old Woman Creek	freshwater wetland soil	wetland metagenome
Candidatus Thorarchaeota archaeon	GCA_004524595	44.6085139 N 27.331375 E	Romania: Amara	lake sediment	sediment metagenome
Candidatus Thorarchaeota archaeon	GCA_004525375	63.29 N 19.49 E	Sweden: Bothnian Sea	Bothnian Sea sediment, site NB8	marine sediment metagenome
Candidatus Thorarchaeota archaeon	GCA_004525645	63.29 N 19.49 E	Sweden: Bothnian Sea	Bothnian Sea sediment, site NB8	marine sediment metagenome
Candidatus Thorarchaeota archaeon	GCA_004524565	44.6085139 N 27.331375 E	Romania: Amara	lake sediment	sediment metagenome
Candidatus Thorarchaeota archaeon	GCA_004524435	44.0552806 N 28.6053 E	Romania: Tekirghiol	lake sediment	sediment metagenome

* genomes from metagenomes reassembled and rebinned in this study

Table S3.

Detailed metadata (accessions, location, environment) for the Mnemosynellales*, Hecatellales*, and Thorarchaeia MAGs in Fig. 6C. For convenience, since there are too many entries, the metadata for Mnemosynellales* and Hecatellales* 16S sequences are presented in Data S14.

REFERENCES AND NOTES

1. P. S. Garcia, S. Gribaldo, G. Borrel, Diversity and evolution of methane-related pathways in Archaea. *Annu. Rev. Microbiol.* **76**, 727–755 (2022).

2. C. Rinke, M. Chuvochina, A. J. Mussig, P.-A. Chaumeil, A. A. Davín, D. W. Waite, W. B. Whitman, D. H. Parks, P. Hugenholtz, A standardized archaeal taxonomy for the Genome Taxonomy Database. *Nat. Microbiol.* **6**, 946–959 (2021).

3. D. H. Parks, M. Chuvochina, D. W. Waite, C. Rinke, A. Skarshewski, P.-A. Chaumeil, P. Hugenholtz, A standardized bacterial taxonomy based on genome phylogeny substantially revises the tree of life. *Nat. Biotechnol.* **36**, 996–1004 (2018).

4. D. H. Parks, M. Chuvochina, P.-A. Chaumeil, C. Rinke, A. J. Mussig, P. Hugenholtz, A complete domain-to-species taxonomy for Bacteria and Archaea. *Nat. Biotechnol.* **38**, 1079–1086 (2020).

5. B. J. Woodcroft, C. M. Singleton, J. A. Boyd, P. N. Evans, J. B. Emerson, A. A. F. Zayed, R. D. Hoelzle, T. O. Lamberton, C. K. McCalley, S. B. Hodgkins, R. M. Wilson, S. O. Purvine, C. D. Nicora, C. Li, S. Frolking, J. P. Chanton, P. M. Crill, S. R. Saleska, V. I. Rich, G. W. Tyson, Genome-centric view of carbon processing in thawing permafrost. *Nature* **560**, 49–54 (2018).

6. D. Y. Sorokin, K. S. Makarova, B. Abbas, M. Ferrer, P. N. Golyshin, E. A. Galinski, S. Ciordia, M. C. Mena, A. Y. Merkel, Y. I. Wolf, M. C. M. van Loosdrecht, E. V. Koonin, Discovery of extremely halophilic, methyl-reducing euryarchaea provides insights into the evolutionary origin of methanogenesis. *Nat. Microbiol.* **2**, 17081 (2017).

7. P. S. Adam, G. Borrel, C. Brochier-Armanet, S. Gribaldo, The growing tree of Archaea: New perspectives on their diversity, evolution and ecology. *ISME J.* **11**, 2407–2425 (2017).

8. Z.-S. Hua, Y.-L. Wang, P. N. Evans, Y.-N. Qu, K. M. Goh, Y.-Z. Rao, Y.-L. Qi, Y.-X. Li, M.-J. Huang, J.-Y. Jiao, Y.-T. Chen, Y.-P. Mao, W.-S. Shu, W. Hozzein, B. P. Hedlund, G. W. Tyson, T. Zhang, W.-J. Li, Insights into the ecological roles and evolution of methyl-coenzyme M reductase-containing hot spring Archaea. *Nat. Commun.* **10**, 1–11 (2019).

9. Y. Wang, G. Wegener, J. Hou, F. Wang, X. Xiao, Expanding anaerobic alkane metabolism in the domain of Archaea. *Nat. Microbiol.* **4**, 595–602 (2019).

10. Y.-F. Liu, J. Chen, L. S. Zaramela, L.-Y. Wang, S. M. Mbadinga, Z.-W. Hou, X.-L. Wu, J.-D. Gu, K. Zengler, B.-Z. Mu, Genomic and transcriptomic evidence supports methane metabolism in *Archaeoglobi*. *mSystems* **5**, e00651-19 (2020).

11. G. Borrel, P. S. Adam, L. J. McKay, L.-X. Chen, I. N. Sierra-García, C. M. K. Sieber, Q. Letourneur, A. Ghozlane, G. L. Andersen, W.-J. Li, S. J. Hallam, G. Muyzer, V. M. de Oliveira, W. P. Inskeep, J. F. Banfield, S. Gribaldo, Wide diversity of methane and short-chain alkane metabolisms in uncultured archaea. *Nat. Microbiol.* **4**, 603–613 (2019).

12. B. A. Berghuis, F. B. Yu, F. Schulz, P. C. Blainey, T. Woyke, S. R. Quake, Hydrogenotrophic methanogenesis in archaeal phylum Verstraetearchaeota reveals the shared ancestry of all methanogens. *Proc. Natl. Acad. Sci. U.S.A.* **116**, 5037–5044 (2019).

13. J. M. Kurth, M. K. Nobu, H. Tamaki, N. de Jonge, S. Berger, M. S. M. Jetten, K. Yamamoto, D. Mayumi, S. Sakata, L. Bai, Methanogenic archaea use a bacteria-like methyltransferase system to demethoxylate aromatic compounds. *ISME J.* **15**, 3549–3565 (2021).

14. J. M. Kurth, H. J. M. Op den Camp, C. U. Welte, Several ways one goal—Methanogenesis from unconventional substrates. *Appl. Microbiol. Biotechnol.* **104**, 6839–6854 (2020).

15. M. K. Nobu, T. Narihiro, K. Kuroda, R. Mei, W.-T. Liu, Chasing the elusive Euryarchaeota class WSA2: Genomes reveal a uniquely fastidious methyl-reducing methanogen. *ISME J.* **10**, 2478–2487 (2016).

16. Y. Wang, G. Wegener, T. A. Williams, R. Xie, J. Hou, C. Tian, Y. Zhang, F. Wang, X. Xiao, A methylotrophic origin of methanogenesis and early divergence of anaerobic multicarbon alkane metabolism. *Sci. Adv.* **7**, eabj1453 (2021).

17. I. Vanwonterghem, P. N. Evans, D. H. Parks, P. D. Jensen, B. J. Woodcroft, P. Hugenholtz, G. W. Tyson, Methylotrophic methanogenesis discovered in the archaeal phylum Verstraetearchaeota. *Nat. Microbiol.* **1**, 16170 (2016).

18. L. J. McKay, M. Dlakić, M. W. Fields, T. O. Delmont, A. M. Eren, Z. J. Jay, K. B. Klingelsmith, D. B. Rusch, W. P. Inskeep, Co-occurring genomic capacity for anaerobic methane and dissimilatory sulfur metabolisms discovered in the Korarchaeota. *Nat. Microbiol.* **4**, 614–622 (2019).

19. R. Laso-Pérez, G. Wegener, K. Knittel, F. Widdel, K. J. Harding, V. Krukenberg, D. V. Meier, M. Richter, H. E. Tegetmeyer, D. Riedel, H.-H. Richnow, L. Adrian, T. Reemtsma, O. J. Lechtenfeld, F. Musat, Thermophilic archaea activate butane via alkyl-coenzyme M formation. *Nature* **539**, 396–401 (2016).

20. S.-C. Chen, N. Musat, O. J. Lechtenfeld, H. Paschke, M. Schmidt, N. Said, D. Popp, F. Calabrese, H. Stryhanyuk, U. Jaekel, Y.-G. Zhu, S. B. Joye, H.-H. Richnow, F. Widdel, F. Musat, Anaerobic oxidation of ethane by archaea from a marine hydrocarbon seep. *Nature* **568**, 108–111 (2019).

21. C. J. Hahn, R. Laso-Pérez, F. Vulcano, K.-M. Vaziourakis, R. Stokke, I. H. Steen, A. Teske, A. Boetius, M. Liebeke, R. Amann, K. Knittel, G. Wegene, "*Candidatus* Ethanoperedens," a thermophilic genus of *archaea* mediating the anaerobic oxidation of ethane. *MBio* **11**, e00600-20 (2020).

22. P. N. Evans, D. H. Parks, G. L. Chadwick, S. J. Robbins, V. J. Orphan, S. D. Golding, G. W. Tyson, Methane metabolism in the archaeal phylum Bathyarchaeota revealed by genome-centric metagenomics. *Science* **350**, 434–438 (2015).

23. K. W. Seitz, N. Dombrowski, L. Eme, A. Spang, J. Lombard, J. R. Sieber, A. P. Teske, T. J. G. Ettema, B. J. Baker, Asgard archaea capable of anaerobic hydrocarbon cycling. *Nat. Commun.* **10**, 1822 (2019).

24. J. A. Boyd, S. P. Jungbluth, A. O. Leu, P. N. Evans, B. J. Woodcroft, G. L. Chadwick, V. J. Orphan, J. P. Amend, M. S. Rappé, G. W. Tyson, Divergent methyl-coenzyme M reductase genes in a deep-subseafloor Archaeoglobi. *ISME J.* **13**, 1269–1279 (2019).

25. A. Bateman, P. Coggill, R. D. Finn, DUFs: Families in search of function. *Acta Crystallogr. Sect. F Struct. Biol. Cryst. Commun.* **66**, 1148–1152 (2010).

26. P. S. Adam, G. Borrel, S. Gribaldo, An archaeal origin of the Wood–Ljungdahl H_4MPT branch and the emergence of bacterial methylotrophy. *Nat. Microbiol.* **4**, 2155–2163 (2019).

27. B. Gao, R. S. Gupta, Phylogenomic analysis of proteins that are distinctive of Archaea and its main subgroups and the origin of methanogenesis. *BMC Genomics* **8**, 86 (2007).

28. A.-K. Kaster, M. Goenrich, H. Seedorf, H. Liesegang, A. Wollherr, G. Gottschalk, R. K. Thauer, More than 200 genes required for methane formation from H_2 and CO_2 and energy conservation are present in *Methanothermobacter marburgensis* and *Methanothermobacter thermautotrophicus*. *Archaea* **2011**, 973848 (2011).

29. T. J. Lie, K. C. Costa, B. Lupa, S. Korpole, W. B. Whitman, J. A. Leigh, Essential anaplerotic role for the energy-converting hydrogenase Eha in hydrogenotrophic methanogenesis. *Proc. Natl. Acad. Sci. U.S.A.* **109**, 15473–15478 (2012).

30. I. Porat, W. Kim, E. L. Hendrickson, Q. Xia, Y. Zhang, T. Wang, F. Taub, B. C. Moore, I. J. Anderson, M. Hackett, J. A. Leigh, W. B. Whitman, Disruption of the operon encoding Ehb hydrogenase limits anabolic CO_2 assimilation in the archaeon *Methanococcus maripaludis*. *J. Bacteriol.* **188**, 1373–1380 (2006).

31. T. A. Major, Y. Liu, W. B. Whitman, Characterization of energy-conserving hydrogenase B in Methanococcus maripaludis. *J. Bacteriol.* **192**, 4022–4030 (2010).

32. D. R. Speth, V. J. Orphan, Metabolic marker gene mining provides insight in global *mcrA* diversity and, coupled with targeted genome reconstruction, sheds further light on metabolic potential of the *Methanomassiliicoccales*. *PeerJ* **6**, e5614 (2018).

33. UniProt Consortium, UniProt: A hub for protein information. *Nucleic Acids Res.* **43**, D204–D212 (2015).

34. K. Raymann, C. Brochier-Armanet, S. Gribaldo, The two-domain tree of life is linked to a new root for the Archaea. *Proc. Natl. Acad. Sci. U.S.A.* **112**, 6670–6675 (2015).

35. Z. Zhou, C. Zhang, P. Liu, L. Fu, R. Laso-Pérez, L. Yang, L. Bai, J. Li, M. Yang, J.-Z. Lin, W.-D. Wang, G. Wegener, M. Li, L. Cheng, Non-syntrophic methanogenic hydrocarbon degradation by an archaeal species. *Nature* **601**, 257–262 (2022).

36. P. S. Adam, G. Borrel, S. Gribaldo, Evolutionary history of carbon monoxide dehydrogenase/acetyl-CoA synthase, one of the oldest enzymatic complexes. *Proc. Natl. Acad. Sci. U.S.A.* **115**, E1166–E1173 (2018).

37. Y.-L. Qi, P. N. Evans, Y.-X. Li, Y.-Z. Rao, Y.-N. Qu, S. Tan, J.-Y. Jiao, Y.-T. Chen, B. P. Hedlund, W.-S. Shu, Z.-S. Hua, W.-J. Li, Comparative genomics reveals thermal adaptation and a high metabolic diversity in "*Candidatus* Bathyarchaeia". *mSystems* **6**, e0025221 (2021).

38. Y. He, M. Li, V. Perumal, X. Feng, J. Fang, J. Xie, S. M. Sievert, F. Wang, Genomic and enzymatic evidence for acetogenesis among multiple lineages of the archaeal phylum Bathyarchaeota widespread in marine sediments. *Nat. Microbiol.* **1**, 16035 (2016).

39. O. N. Lemaire, T. Wagner, A structural view of alkyl-coenzyme M reductases, the first step of alkane anaerobic oxidation catalyzed by archaea. *Biochemistry* **61**, 805–821 (2022).

40. Y. Liu, Z. Zhou, J. Pan, B. J. Baker, J.-D. Gu, M. Li, Comparative genomic inference suggests mixotrophic lifestyle for Thorarchaeota. *ISME J.* **12**, 1021–1031 (2018).

41. D. K. Sydykova, C. O. Wilke, Calculating site-specific evolutionary rates at the amino-acid or codon level yields similar rate estimates. *PeerJ* **5**, e3391 (2017).

42. J. Echave, S. J. Spielman, C. O. Wilke, Causes of evolutionary rate variation among protein sites. *Nat. Rev. Genet.* **17**, 109–121 (2016).

43. G. Gottschalk, R. K. Thauer, The Na^+-translocating methyltransferase complex from methanogenic archaea. *Biochim. Biophys. Acta Bioenerg.* **1505**, 28–36 (2001).

44. I. Mayrose, A. Mitchell, T. Pupko, Site-specific evolutionary rate inference: Taking phylogenetic uncertainty into account. *J. Mol. Evol.* **60**, 345–353 (2005).

45. S. P. Jungbluth, J. P. Amend, M. S. Rappé, Metagenome sequencing and 98 microbial genomes from Juan de Fuca Ridge flank subsurface fluids. *Sci. Data* **4**, 170037 (2017).

46. S. F. Altschul, W. Gish, W. Miller, E. W. Myers, D. J. Lipman, Basic local alignment search tool. *J.*

Mol. Biol. **215**, 403–410 (1990).

47. Y.-F. Liu, J. Chen, Z.-L. Liu, L.-B. Shou, D.-D. Lin, L. Zhou, S.-Z. Yang, J.-F. Liu, W. Li, J.-D. Gu, B.-Z. Mu, Anaerobic degradation of paraffins by thermophilic Actinobacteria under methanogenic conditions. *Environ. Sci. Technol.* **54**, 10610–10620 (2020).

48. R. M. Bowers, N. C. Kyrpides, R. Stepanauskas, M. Harmon-Smith, D. Doud, T. B. K. Reddy, F. Schulz, J. Jarett, A. R. Rivers, E. A. Eloe-Fadrosh, S. G. Tringe, N. N. Ivanova, A. Copeland, A. Clum, E. D. Becraft, R. R. Malmstrom, B. Birren, M. Podar, P. Bork, G. M. Weinstock, G. M. Garrity, J. A. Dodsworth, S. Yooseph, G. Sutton, F. O. Glöckner, J. A. Gilbert, W. C. Nelson, S. J. Hallam, S. P. Jungbluth, T. J. G. Ettema, S. Tighe, K. T. Konstantinidis, W.-T. Liu, B. J. Baker, T. Rattei, J. A. Eisen, B. Hedlund, K. D. McMahon, N. Fierer, R. Knight, R. Finn, G. Cochrane, I. Karsch-Mizrachi, G. W. Tyson, C. Rinke, G. S. Consortium, A. Lapidus, F. Meyer, P. Yilmaz, D. H. Parks, A. M. Eren, L. Schriml, J. F. Banfield, P. Hugenholtz, T. Woyke, Minimum information about a single amplified genome (MISAG) and a metagenome-assembled genome (MIMAG) of bacteria and archaea. *Nat. Biotechnol.* **35**, 725–731 (2017).

49. M. Chuvochina, C. Rinke, D. H. Parks, M. S. Rappé, G. W. Tyson, P. Yilmaz, W. B. Whitman, P. Hugenholtz, The importance of designating type material for uncultured taxa. *Syst. Appl. Microbiol.* **42**, 15–21 (2019).

50. A. E. Murray, J. Freudenstein, S. Gribaldo, R. Hatzenpichler, P. Hugenholtz, P. Kämpfer, K. T. Konstantinidis, C. E. Lane, R. T. Papke, D. H. Parks, R. Rossello-Mora, M. B. Stott, I. C. Sutcliffe, J. C. Thrash, S. N. Venter, W. B. Whitman, S. G. Acinas, R. I. Amann, K. Anantharaman, J. Armengaud, B. J. Baker, R. A. Barco, H. B. Bode, E. S. Boyd, C. L. Brady, P. Carini, P. S. G. Chain, D. R. Colman, K. M. De Angelis, M. A. de Los Rios, P. Estrada-de Los Santos, C. A. Dunlap, J. A. Eisen, D. Emerson, T. J. G. Ettema, D. Eveillard, P. R. Girguis, U. Hentschel, J. T. Hollibaugh, L. A. Hug, W. P. Inskeep, E. P. Ivanova, H.-P. Klenk, W.-J. Li, K. G. Lloyd, F. E. Löffler, T. P. Makhalanyane, D. P. Moser, T. Nunoura, M. Palmer, V. Parro, C. Pedrós-Alió, A. J. Probst, T. H. M. Smits, A. D. Steen, E. T. Steenkamp, A. Spang, F. J. Stewart, J. M. Tiedje, P. Vandamme, M. Wagner, F.-P. Wang, P. Yarza, B. P. Hedlund, A.-L. Reysenbach, Roadmap for naming uncultivated Archaea and Bacteria. *Nat. Microbiol.* **5**, 987–994 (2020).

51. J.-W. Zhang, H.-P. Dong, L.-J. Hou, Y. Liu, Y.-F. Ou, Y.-L. Zheng, P. Han, X. Liang, G.-Y. Yin, D.-M. Wu, M. Liu, M. Li, Newly discovered Asgard archaea Hermodarchaeota potentially degrade alkanes and aromatics via alkyl/benzyl-succinate synthase and benzoyl-CoA pathway. *ISME J.* **15**, 1826–1843 (2021).

52. I. F. Farag, J. F. Biddle, R. Zhao, A. J. Martino, C. H. House, R. I. León-Zayas, Metabolic potentials of archaeal lineages resolved from metagenomes of deep Costa Rica sediments. *ISME J.* **14**, 1345–1358 (2020).

53. R. D. Finn, J. Clements, S. R. Eddy, HMMER web server: Interactive sequence similarity searching. *Nucleic Acids Res.* **39**, W29–W37 (2011).

54. S. El-Gebali, J. Mistry, A. Bateman, S. R. Eddy, A. Luciani, S. C. Potter, M. Qureshi, L. J. Richardson, G. A. Salazar, A. Smart, E. L. L. Sonnhammer, L. Hirsh, L. Paladin, D. Piovesan, S. C. E. Tosatto, R. D. Finn, The Pfam protein families database in 2019. *Nucleic Acids Res.* **47**, D427–D432 (2018).

55. J. Huerta-Cepas, D. Szklarczyk, D. Heller, A. Hernández-Plaza, S. K. Forslund, H. Cook, D. R. Mende, I. Letunic, T. Rattei, L. J. Jensen, C. von Mering, P. Bork, eggNOG 5.0: A hierarchical, functionally and phylogenetically annotated orthology resource based on 5090 organisms and 2502 viruses. *Nucleic Acids Res.* **47**, D309–D314 (2019).

56. B. Buchfink, C. Xie, D. H. Huson, Fast and sensitive protein alignment using DIAMOND. *Nat. Methods* **12**, 59–60 (2015).

57. R. C. Edgar, MUSCLE: Multiple sequence alignment with high accuracy and high throughput. *Nucleic Acids Res.* **32**, 1792–1797 (2004).

58. A. Criscuolo, S. Gribaldo, BMGE (Block Mapping and Gathering with Entropy): A new software for selection of phylogenetic informative regions from multiple sequence alignments. *BMC Evol. Biol.* **10**, 210 (2010).

59. B. Q. Minh, H. A. Schmidt, O. Chernomor, D. Schrempf, M. D. Woodhams, A. von Haeseler, R. Lanfear, IQ-TREE 2: New models and efficient methods for phylogenetic inference in the genomic era. *Mol. Biol. Evol.* **37**, 1530–1534 (2020).

60. S. Kalyaanamoorthy, B. Q. Minh, T. K. F. Wong, A. von Haeseler, L. S. Jermiin, ModelFinder: Fast model selection for accurate phylogenetic estimates. *Nat. Methods* **14**, 587–589 (2017).

61. D. T. Hoang, O. Chernomor, A. von Haeseler, B. Q. Minh, L. S. Vinh, UFBoot2: Improving the ultrafast bootstrap approximation. *Mol. Biol. Evol.* **35**, 518–522 (2017).

62. S. Guindon, J.-F. Dufayard, V. Lefort, M. Anisimova, W. Hordijk, O. Gascuel, New algorithms and methods to estimate maximum-likelihood phylogenies: Assessing the performance of PhyML 3.0. *Syst. Biol.* **59**, 307–321 (2010).

63. M. Anisimova, M. Gil, J.-F. Dufayard, C. Dessimoz, O. Gascuel, Survey of branch support methods demonstrates accuracy, power, and robustness of fast likelihood-based approximation schemes. *Syst. Biol.* **60**, 685–699 (2011).

64. I. Letunic, P. Bork, Interactive Tree Of Life (iTOL) v5: An online tool for phylogenetic tree display and annotation. *Nucleic Acids Res.* **49**, W293–W296 (2021).

65. K. Kobert, L. Salichos, A. Rokas, A. Stamatakis, Computing the internode certainty and related measures from partial gene trees. *Mol. Biol. Evol.* **33**, 1606–1617 (2016).

66. A. Stamatakis, RAxML version 8: A tool for phylogenetic analysis and post-analysis of large phylogenies. *Bioinformatics* **30**, 1312–1313 (2014).

67. H.-C. Wang, B. Q. Minh, E. Susko, A. J. Roger, Modeling site heterogeneity with posterior mean site frequency profiles accelerates accurate phylogenomic estimation. *Syst. Biol.* **67**, 216–235 (2018).

68. P. S. Garcia, F. Jauffrit, C. Grangeasse, C. Brochier-Armanet, GeneSpy, a user-friendly and flexible genomic context visualizer. *Bioinformatics* **35**, 329–331 (2019).

69. F. D. K. Tria, G. Landan, T. Dagan, Phylogenetic rooting using minimal ancestor deviation. *Nat. Ecol. Evol.* **1**, 193 (2017).

70. U. Mai, E. Sayyari, S. Mirarab, Minimum variance rooting of phylogenetic trees and implications for species tree reconstruction. *PLOS ONE* **12**, e0182238 (2017).

71. S. Naser-Khdour, B. Q. Minh, R. Lanfear, Assessing confidence in root placement on phylogenies: An empirical study using nonreversible models for mammals. *Syst. Biol.* **71**, 959–972 (2022).

72. B. Q. Minh, M. W. Hahn, R. Lanfear, New methods to calculate concordance factors for phylogenomic datasets. *Mol. Biol. Evol.* **37**, 2727–2733 (2020).

73. J. L. Steenwyk, T. J. Buida III, Y. Li, X.-X. Shen, A. Rokas, ClipKIT: A multiple sequence alignment trimming software for accurate phylogenomic inference. *PLOS Biol.* **18**, e3001007 (2020).

74. A. Krogh, B. Larsson, G. Von Heijne, E. L. L. Sonnhammer, Predicting transmembrane protein topology with a hidden Markov model: Application to complete genomes. *J. Mol. Biol.* **305**, 567–580 (2001).

75. L. Käll, A. Krogh, E. L. L. Sonnhammer, Advantages of combined transmembrane topology and signal peptide prediction—The Phobius web server. *Nucleic Acids Res.* **35**, W429–W432 (2007).

76. K. D. Tsirigos, C. Peters, N. Shu, L. Käll, A. Elofsson, The TOPCONS web server for consensus prediction of membrane protein topology and signal peptides. *Nucleic Acids Res.* **43**, W401–W407 (2015).

77. M. Kanehisa, Y. Sato, K. Morishima, BlastKOALA and GhostKOALA: KEGG tools for functional characterization of genome and metagenome sequences. *J. Mol. Biol.* **428**, 726–731 (2016).

78. D. Søndergaard, C. N. S. Pedersen, C. Greening, HydDB: A web tool for hydrogenase classification and analysis. *Sci. Rep.* **6**, 34212 (2016).

79. H. Zhang, T. Yohe, L. Huang, S. Entwistle, P. Wu, Z. Yang, P. K. Busk, Y. Xu, Y. Yin, dbCAN2: A meta server for automated carbohydrate-active enzyme annotation. *Nucleic Acids Res.* **46**, W95–W101 (2018).

80. N. D. Rawlings, A. J. Barrett, P. D. Thomas, X. Huang, A. Bateman, R. D. Finn, The MEROPS database of proteolytic enzymes, their substrates and inhibitors in 2017 and a comparison with peptidases in the PANTHER database. *Nucleic Acids Res.* **46**, D624–D632 (2018).

81. C. M. K. Sieber, A. J. Probst, A. Sharrar, B. C. Thomas, M. Hess, S. G. Tringe, J. F. Banfield, Recovery of genomes from metagenomes via a dereplication, aggregation and scoring strategy. *Nat. Microbiol.* **3**, 836–843 (2018).

82. L. Fu, B. Niu, Z. Zhu, S. Wu, W. Li, CD-HIT: Accelerated for clustering the next-generation sequencing data. *Bioinformatics* **28**, 3150–3152 (2012).

83. M. R. Olm, C. T. Brown, B. Brooks, J. F. Banfield, dRep: A tool for fast and accurate genomic comparisons that enables improved genome recovery from metagenomes through de-replication. *ISME J.* **11**, 2864–2868 (2017).

84. M. Gouy, S. Guindon, O. Gascuel, SeaView version 4: A multiplatform graphical user interface for sequence alignment and phylogenetic tree building. *Mol. Biol. Evol.* **27**, 221–224 (2009).

85. O. Gascuel, BIONJ: An improved version of the NJ algorithm based on a simple model of sequence data. *Mol. Biol. Evol.* **14**, 685–695 (1997).

86. L. A. Kelley, S. Mezulis, C. M. Yates, M. N. Wass, M. J. E. Sternberg, The Phyre2 web portal for protein modeling, prediction and analysis. *Nat. Protoc.* **10**, 845–858 (2015).

87. W. L. DeLano, Pymol: An open-source molecular graphics tool. *CCP4 Newsl. Protein Crystallogr.* **40**, 82–92 (2002).

88. S. J. Spielman, S. L. Kosakovsky Pond, Relative evolutionary rates in proteins are largely insensitive to the substitution model. *Mol. Biol. Evol.* **35**, 2307–2317 (2018).

89. D. K. Sydykova, C. O. Wilke, Theory of measurement for site-specific evolutionary rates in amino-acid sequences. bioRxiv 411025 [Preprint]. 7 September 2018. https://doi.org/10.1101/411025.

90. N. A. Joshi, J. Fass, Sickle: A sliding-window, adaptive, quality-based trimming tool for FastQ files (version 1.33) [Software] (2011).

91. S. Nurk, D. Meleshko, A. Korobeynikov, P. A. Pevzner, metaSPAdes: A new versatile metagenomic assembler. *Genome Res.* **27**, 824–834 (2017).

92. T. L. V. Bornemann, S. P. Esser, T. L. Stach, T. Burg, A. J. Probst, uBin—A manual refining tool for metagenomic bins designed for educational purposes. bioRxiv 2020.07.15.204776 [Preprint]. 24 July 2020. https://doi.org/10.1101/2020.07.15.204776.

93. C. T. Brown, M. R. Olm, B. C. Thomas, J. F. Banfield, Measurement of bacterial replication rates in microbial communities. *Nat. Biotechnol.* **34**, 1256–1263 (2016).

94. Y.-W. Wu, B. A. Simmons, S. W. Singer, MaxBin 2.0: An automated binning algorithm to recover genomes from multiple metagenomic datasets. *Bioinformatics* **32**, 605–607 (2016).

95. A. J. Probst, C. J. Castelle, A. Singh, C. T. Brown, K. Anantharaman, I. Sharon, L. A. Hug, D. Burstein, J. B. Emerson, B. C. Thomas, J. F. Banfield, Genomic resolution of a cold subsurface aquifer community provides metabolic insights for novel microbes adapted to high CO_2 concentrations. *Environ. Microbiol.* **19**, 459–474 (2017).

96. D. H. Parks, M. Imelfort, C. T. Skennerton, P. Hugenholtz, G. W. Tyson, CheckM: Assessing the quality of microbial genomes recovered from isolates, single cells, and metagenomes. *Genome Res.* **25**, 1043–1055 (2015).

97. T. Seemann, Prokka: Rapid prokaryotic genome annotation. *Bioinformatics* **30**, 2068–2069 (2014).

98. A. E. Darling, G. Jospin, E. Lowe, F. A. Matsen IV, H. M. Bik, J. A. Eisen, PhyloSift: Phylogenetic analysis of genomes and metagenomes. *PeerJ* **2**, e243 (2014).

99. V. De Anda, L.-X. Chen, N. Dombrowski, Z.-S. Hua, H.-C. Jiang, J. F. Banfield, W.-J. Li, B. J. Baker, Brockarchaeota, a novel archaeal phylum with unique and versatile carbon cycling pathways. *Nat. Commun.* **12**, 2404 (2021).

100. S. M. Crotty, B. Q. Minh, N. G. Bean, B. R. Holland, J. Tuke, L. S. Jermiin, A. Von Haeseler, GHOST: Recovering historical signal from heterotachously evolved sequence alignments. *Syst. Biol.* **69**, 249–264 (2020).

101. E. Susko, A. J. Roger, On reduced amino acid alphabets for phylogenetic inference. *Mol. Biol. Evol.* **24**, 2139–2150 (2007).

102. T. Pupko, R. E. Bell, I. Mayrose, F. Glaser, N. Ben-Tal, Rate4Site: An algorithmic tool for the identification of functional regions in proteins by surface mapping of evolutionary determinants within their homologues. *Bioinformatics* **18**, S71–S77 (2002).

103. I. Lee, Y. O. Kim, S.-C. Park, J. Chun, OrthoANI: An improved algorithm and software for calculating average nucleotide identity. *Int. J. Syst. Evol. Microbiol.* **66**, 1100–1103 (2016).

104. K. Katoh, D. M. Standley, MAFFT multiple sequence alignment software version 7: Improvements in performance and usability. *Mol. Biol. Evol.* **30**, 772–780 (2013).

105. R Core Team, *R: A Language and Environment for Statistical Computing* (R Foundation for Statistical Computing, 2020); https://r-project.org/.

106. A. Dinno, dunn. test: Dunn's test of multiple comparisons using rank sums. R package version 1.3. 5 (2017).

107. W. Hadley, *Ggplot2: Elegrant Graphics for Data Analysis* (Springer, 2016).

108. P.-A. Chaumeil, A. J. Mussig, P. Hugenholtz, D. H. Parks, GTDB-Tk: A toolkit to classify genomes with the Genome Taxonomy Database (2020).

109. S. Kato, M. Ohnishi, M. Nagamori, M. Yuki, T. Takashina, M. Ohkuma, T. Itoh, *Conexivisphaera calida* gen. nov., sp. nov., a thermophilic sulfur-and iron-reducing archaeon, and proposal of *Conexivisphaeraceae* fam. nov., *Conexivisphaerales* ord. nov., and *Conexivisphaeria* class. nov. in the phylum *Thaumarchaeota*. *Int. J. Syst. Evol. Microbiol.* **71**, 4595 (2021).

110. J. Li, L. Qi, Y. Guo, L. Yue, Y. Li, W. Ge, J. Wu, W. Shi, X. Dong, Global mapping transcriptional start sites revealed both transcriptional and post-transcriptional regulation of cold adaptation in the methanogenic archaeon *Methanolobus psychrophilus*. *Sci. Rep.* **5**, 9209 (2015).

111. D. Prakash, Y. Wu, S.-J. Suh, E. C. Duin, Elucidating the process of activation of methyl-coenzyme M reductase. *J. Bacteriol.* **196**, 2491–2498 (2014).

112. M. Ren, X. Feng, Y. Huang, H. Wang, Z. Hu, S. Clingenpeel, B. K. Swan, M. M. Fonseca, D.

Posada, R. Stepanauskas, J. T. Hollibaugh, P. G. Foster, T. Woyke, H. Lou, Phylogenomics suggests oxygen availability as a driving force in Thaumarchaeota evolution. *ISME J.* **13**, 2150–2161 (2019).

113. C. H. Kuhner, B. D. Lindenbach, R. S. Wolfe, Component A2 of methylcoenzyme M reductase system from Methanobacterium thermoautotrophicum delta H: Nucleotide sequence and functional expression by Escherichia coli. *J. Bacteriol.* **175**, 3195–3203 (1993).

114. K. Zheng, P. D. Ngo, V. L. Owens, X. Yang, S. O. Mansoorabadi, The biosynthetic pathway of coenzyme F430 in methanogenic and methanotrophic archaea. *Science* **354**, 339–342 (2016).

115. S. Mayr, C. Latkoczy, M. Krüger, D. Günther, S. Shima, R. K. Thauer, F. Widdel, B. Jaun, Structure of an F430 variant from archaea associated with anaerobic oxidation of methane. *J. Am. Chem. Soc.* **130**, 10758–10767 (2008).

116. S. Shima, M. Krueger, T. Weinert, U. Demmer, J. Kahnt, R. K. Thauer, U. Ermler, Structure of a methyl-coenzyme M reductase from Black Sea mats that oxidize methane anaerobically. *Nature* **481**, 98–101 (2012).

117. U. Harms, R. K. Thauer, Identification of the active site histidine in the corrinoid protein MtrA of the energy-conserving methyltransferase complex from methanobacterium thermoautotrophicum. *Eur. J. Biochem.* **250**, 783–788 (1997).

118. D. H. Shin, Preliminary structural studies on the MtxX protein from Methanococcus jannaschii. *Acta Crystallogr. Sect. F Struct. Biol. Cryst. Commun.* **64**, 300–303 (2008).

119. Y. Ikeuchi, S. Kimura, T. Numata, D. Nakamura, T. Yokogawa, T. Ogata, T. Wada, T. Suzuki, T. Suzuki, Agmatine-conjugated cytidine in a tRNA anticodon is essential for AUA decoding in archaea. *Nat. Chem. Biol.* **6**, 277–282 (2010).

120. D. D. Nayak, N. Mahanta, D. A. Mitchell, W. W. Metcalf, Post-translational thioamidation of methyl-coenzyme M reductase, a key enzyme in methanogenic and methanotrophic Archaea. *eLife* **6**, e29218 (2017).

121. D. Deobald, L. Adrian, C. Schöne, M. Rother, G. Layer, Identification of a unique radical SAM

methyltransferase required for the sp^3-C-methylation of an arginine residue of methyl-coenzyme M reductase. *Sci. Rep.* **8**, 7404 (2018).

122. M. I. Radle, D. V. Miller, T. N. Laremore, S. J. Booker, Methanogenesis marker protein 10 (Mmp10) from *Methanosarcina acetivorans* is a radical *S*-adenosylmethionine methylase that unexpectedly requires cobalamin. *J. Biol. Chem.* **294**, 11712–11725 (2019).

123. C. D. Fyfe, N. Bernardo-García, L. Fradale, S. Grimaldi, A. Guillot, C. Brewee, L. M. G. Chavas, P. Legrand, A. Benjdia, O. Berteau, Crystallographic snapshots of a B12-dependent radical SAM methyltransferase. *Nature* **602**, 336–342 (2022).

124. D. D. Nayak, A. Liu, N. Agrawal, R. Rodriguez-Carerro, S.-H. Dong, D. A. Mitchell, S. K. Nair, W. W. Metcalf, Functional interactions between posttranslationally modified amino acids of methyl-coenzyme M reductase in Methanosarcina acetivorans. *PLOS Biol.* **18**, e3000507 (2020).

125. J. M. Kurth, M.-C. Müller, C. U. Welte, T. Wagner, Structural insights into the methane-generating enzyme from a methoxydotrophic methanogen reveal a restrained gallery of post-translational modifications. *Microorganisms* **9**, 837 (2021).

126. T. J. Lie, K. C. Costa, D. Pak, V. Sakesan, J. A. Leigh, Phenotypic evidence that the function of the [Fe]-hydrogenase Hmd in Methanococcus maripaludis requires seven hcg (hmd co-occurring genes) but not hmdII. *FEMS Microbiol. Lett.* **343**, 156–160 (2013).

127. C. Brochier-Armanet, P. Forterre, S. Gribaldo, Phylogeny and evolution of the Archaea: One hundred genomes later. *Curr. Opin. Microbiol.* **14**, 274–281 (2011).

128. C. Petitjean, P. Deschamps, P. López-García, D. Moreira, C. Brochier-Armanet, Extending the conserved phylogenetic core of archaea disentangles the evolution of the third domain of life. *Mol. Biol. Evol.* **32**, 1242–1254 (2015).

129. A. Tersteegen, R. Hedderich, Methanobacterium thermoautotrophicum encodes two multisubunit membrane-bound [NiFe] hydrogenases: Transcription of the operons and sequence analysis of the deduced proteins. *Eur. J. Biochem.* **264**, 930–943 (1999).

130. N. Dombrowski, A. P. Teske, B. J. Baker, Expansive microbial metabolic versatility and biodiversity in dynamic Guaymas Basin hydrothermal sediments. *Nat. Commun.* **9**, 4999 (2018).

131. S. P. Jungbluth, R. M. Bowers, H.-T. Lin, J. P. Cowen, M. S. Rappé, Novel microbial assemblages inhabiting crustal fluids within mid-ocean ridge flank subsurface basalt. *ISME J.* **10**, 2033–2047 (2016).

132. H. Q. Loh, V. Hervé, A. Brune, Metabolic potential for reductive acetogenesis and a novel energy-converting [NiFe] hydrogenase in *Bathyarchaeia* from termite guts–a genome-centric analysis. *Front. Microbiol.* **11**, 3644 (2021).

133. S. Watanabe, Y. Murase, Y. Watanabe, Y. Sakurai, K. Tajima, Crystal structures of aconitase X enzymes from bacteria and archaea provide insights into the molecular evolution of the aconitase superfamily. *Commun. Biol.* **4**, 687 (2021).

134. J. M. Tor, D. R. Lovley, Anaerobic degradation of aromatic compounds coupled to Fe (III) reduction by Ferroglobus placidus. *Environ. Microbiol.* **3**, 281–287 (2001).

135. C. K. Y. Lau, K. D. Krewulak, H. J. Vogel, Bacterial ferrous iron transport: The Feo system. *FEMS Microbiol. Rev.* **40**, 273–298 (2016).

136. G. B. Slobodkina, A. V. Mardanov, N. V. Ravin, A. A. Frolova, N. A. Chernyh, E. A. Bonch-Osmolovskaya, A. I. Slobodkin, Respiratory ammonification of nitrate coupled to anaerobic oxidation of elemental sulfur in deep-sea autotrophic thermophilic bacteria. *Front. Microbiol.* **8**, 87 (2017).

137. Z. F. Islam, C. Welsh, K. Bayly, R. Grinter, G. Southam, E. J. Gagen, C. Greening, A widely distributed hydrogenase oxidises atmospheric H2 during bacterial growth. *ISME J.* **14**, 2649–2658 (2020).

138. S. A. Carr, S. P. Jungbluth, E. A. Eloe-Fadrosh, R. Stepanauskas, T. Woyke, M. S. Rappé, B. N. Orcutt, Carboxydotrophy potential of uncultivated Hydrothermarchaeota from the subseafloor crustal biosphere. *ISME J.* **13**, 1457–1468 (2019).

139. Z. Zhou, Y. Liu, W. Xu, J. Pan, Z.-H. Luo, M. Li, Genome- and community-level interaction insights into carbon utilization and element cycling functions of *Hydrothermarchaeota* in hydrothermal

sediment. *Msystems* **5**, e00795-19 (2020).

140. T. M. Chicano, L. Dietrich, N. M. de Almeida, M. Akram, E. Hartmann, F. Leidreiter, D. Leopoldus, M. Mueller, R. Sánchez, G. H. L. Nuijten, J. Reimann, K.-A. Seifert, I. Schlichting, L. van Niftrik, M. S. M. Jetten, A. Dietl, B. Kartal, K. Parey, T. R. M. Barends, Structural and functional characterization of the intracellular filament-forming nitrite oxidoreductase multiprotein complex. *Nat. Microbiol.* **6**, 1129–1139 (2021).

141. J. Oberto, SyntTax: A web server linking synteny to prokaryotic taxonomy. *BMC Bioinformatics* **14**, 4 (2013).
